# Impact of animal strain on gene expression in a rat model of acute cardiac rejection

**DOI:** 10.1186/1471-2164-10-280

**Published:** 2009-06-24

**Authors:** Katherine J Deans, Peter C Minneci, Hao Chen, Steven J Kern, Carolea Logun, Sara Alsaaty, Kelly J Norsworthy, Stephanie M Theel, Joel D Sennesh, Jennifer J Barb, Peter J Munson, Robert L Danner, Michael A Solomon

**Affiliations:** 1 Critical Care Medicine Department, Clinical Center, NIH, Bethesda, MD, USA; 2 Mathematical and Statistical Computing Laboratory, Division of Computational Bioscience, Center for Information Technology, NIH, Bethesda, MD, USA; 3 Department of Pathology, Inova Fairfax Hospital, Fairfax, VA, USA; 4 Department of Surgery, The Children's Institute for Surgical Science, The Children's Hospital of Philadelphia, Philadelphia, PA, USA; 5 Department of Surgery, University of Pennsylvania School of Medicine, Philadelphia, PA, USA; 6 Cardiovascular Branch, NHLBI, NIH, Bethesda, MD, USA

## Abstract

**Background:**

The expression levels of many genes show wide natural variation among strains or populations. This study investigated the potential for animal strain-related genotypic differences to confound gene expression profiles in acute cellular rejection (ACR). Using a rat heart transplant model and 2 different rat strains (Dark Agouti, and Brown Norway), microarrays were performed on native hearts, transplanted hearts, and peripheral blood mononuclear cells (PBMC).

**Results:**

In heart tissue, strain alone affected the expression of only 33 probesets while rejection affected the expression of 1368 probesets (FDR 10% and FC ≥ 3). Only 13 genes were affected by both strain and rejection, which was < 1% (13/1368) of all probesets differentially expressed in ACR. However, for PBMC, strain alone affected 265 probesets (FDR 10% and FC ≥ 3) and the addition of ACR had little further effect. Pathway analysis of these differentially expressed strain effect genes connected them with immune response, cell motility and cell death, functional themes that overlap with those related to ACR. After accounting for animal strain, additional analysis identified 30 PBMC candidate genes potentially associated with ACR.

**Conclusion:**

In ACR, genetic background has a large impact on the transcriptome of immune cells, but not heart tissue. Gene expression studies of ACR should avoid study designs that require cross strain comparisons between leukocytes.

## Background

Acute cellular rejection (ACR) is a major cause of morbidity and mortality among cardiac transplant patients [1-3]. Prompt diagnosis with early intervention by appropriate adjustment of immunosuppressive medications can reverse ACR, while delayed treatment of ACR can lead to graft injury or loss. Conversely, unnecessary escalation of immunosuppression exposes patients to an increased risk of infections that can also be life-threatening [4]. Unfortunately, symptoms and signs of ACR are often nonspecific. Diagnosis relies on serial cardiac biopsies, an invasive and costly procedure. In addition, ACR in its early stages can be a patchy process such that histopathologic examination of heart tissue can both under- and over-diagnose its presence [5,6]. Noninvasive, sensitive, and specific tests that reliably detect ACR in its earliest stages would greatly simplify the management of cardiac transplant patients, increase graft survival, and improve clinical outcomes. These issues combined with the advent of high-throughput functional genomic and proteomic methodologies have fueled a search for ACR biomarkers, as well as new therapeutic targets.

To date, clinical studies have not convincingly identified ACR biomarkers that appear suitable for diagnostic testing across diverse patient populations [7]. Observational gene discovery studies have been performed in ACR [8]. However, proposed panels based on gene expression changes in blood lack biological plausibility and independent replication [7]. Background noise from genotypic heterogeneity may have hampered these investigations. Proof of principle experiments using animal models of ACR that impose uniformity not achievable in clinical studies have also attempted to find candidate biomarkers. However, many of these studies have directly compared cells and tissues that originated from different animal strains [9-14]. Underlying genotypic differences have the potential to confound these experiments and lead to erroneous conclusions. Furthermore, this source of error is compounded and magnified in high-dimension, discovery-driven platforms such as microarrays that measure thousands of endpoints.

Natural variation in gene expression is known to be extensive across human populations [15-18] and animal strains [19-22]. Depending on the tissue and mouse strains examined, genotypic background appears to significantly affect the expression of 1 to 2% of the entire transcriptome [20-22]. These studies raise legitimate concerns about our ability to distinguish signal (phenotype of interest) from noise (heterogeneity or strain effects) in biomarker discovery studies. While genetic background can potentially influence the results of any study, animal investigations that require the use of more than one strain are at particular risk. Strain differences in animals and heterogeneity across human populations may significantly influence the transcriptomes of individuals to the extent that phenotypic differences of interest such as non-rejecting versus rejecting may be difficult or impossible to detect.

To date, the impact of strain differences or in essence genotypic heterogeneity on transcriptomic profiling has not been investigated in animal models of organ transplantation. Moreover, strain effects have not been quantified in tissues of interest nor have differences been thematically analyzed to determine whether study interpretation might be jeopardized. Here, the potential confounding effects of animal strain differences on expression profiling was examined in a heterotopic rat heart transplant model. RNA from native hearts, transplanted hearts, and peripheral blood mononuclear cells (PBMC) from normal and transplanted animals were interrogated using high-density oligonucleotide microarrays and analyzed for effects attributable to animal strain as well as rejection. Understanding the impact of genotypic heterogeneity on transcriptomic profiles is likely to improve experimental designs, increasing scientific accuracy for identifying promising biomarkers.

## Methods

### Animal Care

The protocol described in the current study was approved by the Animal Care and Use Committee (ACUC) of the Clinical Center of the National Institutes of Health (NIH). Animal care followed the criteria of the ACUC of the Clinical Center of the NIH.

### Histology

All specimens were processed for histopathology using hematoxylin and eosin staining. Histological changes were blindly assessed by a pathologist, using the International Society for Heart and Lung Transplantation (ISHLT) grading system for rejection [23,24].

### Heterotopic Cardiac Transplantation

Fourteen cardiac transplantations (9 isogeneic and 5 allogeneic) were performed using a modified version of the heterotopic cardiac transplantation model reported by Yokoyama *et al *[25]. Briefly, after heparinization, donor hearts were procured from Dark Agouti (DA) animals, flushed with Lactated Ringers, and prepared for transplantation with ligation of pulmonary vessels, creation of an atrial septal defect and disruption of the tricuspid valve leaflets via a right atriotomy. In isogeneic transplants, the recipient animal strain was Dark Agouti (DA to DA), and in allogeneic transplants the recipient animal strain was Brown Norway (DA to BN). In this model, the animal strains have major antigen mismatches and allografts lose pulsatility on post-transplant day 6 with histologic ISHLT grade 3R rejection. Heterotopic transplantation was performed by anastomosis of the donor ascending aorta to the recipient abdominal aorta and the donor right atrium to the recipient inferior vena cava using microsurgical techniques. Upon re-establishment of blood flow, all transplanted hearts resumed spontaneous contractions, had coordinated atrioventricular activity, and were free of gross surgical injury at the time of closure.

### Specimen Procurement

**Figure 1:**
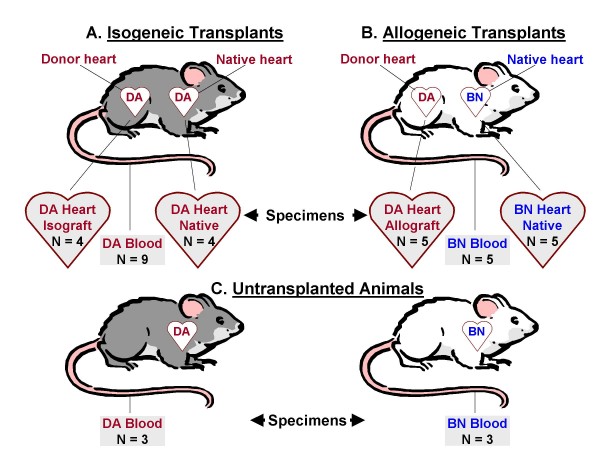
**Experimental groups and specimen procurement**. **(A) **Isogeneic transplants consisted of a strain DA donor heart placed into the abdomen of a strain DA recipient rat. Specimens procured from isogeneic transplants consisted of strain DA isograft and native heart, and strain DA blood.**(B) **Allogeneic transplants consisted of a strain DA donor heart placed into the abdomen of a strain BN recipient rat. Specimens procured from allogeneic transplants consisted of strain DA allograft and strain BN native heart, and strain BN blood. **(C) **Untransplanted animals were strain DA and BN rats that did not receive an isograft or an allograft heart. Specimens procured from untransplanted animals consisted of strain DA and strain BN blood.

On post transplant day 6, specimens procured from isogeneic transplants (Figure 1A) consisted of DA isograft hearts (n = 4), DA native hearts (n = 4), and DA PBMC (n = 9). On post transplant day 6, specimens procured from allogeneic transplants (Figure 1B) consisted of DA allograft hearts (n = 5), BN native hearts (n = 5), and BN PBMC (n = 5). Specimens procured from untransplanted animals (Figure 1C) consisted of DA PBMC (n = 3) and BN PBMC (n = 3). Cross-sections of both ventricles and the interventricular septum were preserved in RNAlater (Ambion Inc., Austin, TX) for oligonucleotide array analysis and frozen at -70°C until processing.

### Microarrays

PBMC were isolated using the Nycoprep density gradient (Axis-Shield, Oslo, Norway). A cell count and differential were performed on all samples. Total RNA was prepared from PBMC using the RNeasy Mini kit with DNase treatment (Qiagen Inc., Valencia, CA). For PBMC, messageAmp II aRNA kit (Ambion Inc.) was used to process total RNA to cDNA and cRNA. Total RNA from heart tissue was prepared using RNeasy Mini kits with DNAase and Proteinase K treatment (Qiagen Inc). For heart tissue, total RNA (10 μg) was reverse transcribed using the SuperScript II Custom kit (Invitrogen, Carlsbad, CA). cDNA cleanup was performed using cDNA Sample Cleanup modules (Affymetrix, Santa Clara, CA). cDNA (1 μg) was used as a template for *in vitro *transcription and biotin labeling reaction using a BioArray High Yield kit (Enzo Life Sciences, Farmingdale, NY). cRNA cleanup was performed using cRNA Sample Cleanup modules (Affymetrix).

Fragmentation and hybridization was performed according to Affymetrix standard methodology. The Affymetrix RAE230A and RAE230 2.0 microarray chips were used for heart tissue samples and PBMC respectively. Microarrays were washed and stained using the standard format for the Affymetrix Fluidics Station (Affymetrix). The probe arrays were scanned using the Affymetrix Scanner G-3000. GeneChip Operating System (GSOS; Affymetrix) was used to quantify gene expression. Result quality was assessed by comparison to historical values for this laboratory.

### Quantitative PCR

TaqMan^® ^(ABI, Rockville, MD) quantitative real time – polymerase chain reaction (qRT-PCR) was utilized to quantify mRNA levels. Sufficient total RNA was available from the PBMC preparations of 3 isogeneic transplants, 3 allogeneic transplants and 5 untransplanted rats. Gene specific probes and PCR primers for glyceraldehyde 3-phosphate dehydrogenase (GAPDH), chemokine (c-c motif) ligand 9 (Ccl9), integrin alpha L (Itgal), s100 calcium binding protein A9 (S100a9), granzyme B (Gzmb), pancreatic trypsin 1 (Prss1), and lectin galactose binding soluble 5 (Lgals5) were purchased from ABI (Foster City, CA). The High-capacity cDNA Archive kit (ABI, Foster City, CA) was used to prepare cDNA from 2 μg of total RNA. Resulting cDNA was used for qRT-PCR in triplicate according to the standard ABI protocol. The target mRNA of Ccl9, Itgal, S100a9, Gzmb, Prss1 and Lgals5 were normalized to GAPDH. Relative mRNA amounts were calculated as previously described [26]. Final results were expressed as fold change.

### Oligonucleotide Microarray Data Analysis

Output from Affymetrix GCOS (Gene Chip Operating Software, Affymetrix, Inc. Santa Clara, CA) was stored in the NIHGCOS database. Affymetrix signal intensities were retrieved and assembled for further statistical analysis using MSCL Analysts Toolbox [27], a microarray analysis package that uses custom written scripts for JMP (SAS Institute, Cary, NC). Signal intensities were normalized to median values and log transformed. The data discussed in this publication have been deposited in the National Center for Biotechnology Information's (NCBI) Gene Expression Omnibus (GEO) [28] and are accessible through GEO series accession number GSE6342 [29].

Principal component analysis (PCA), a tool for visualizing a multivariate response, was performed on the entire gene chip and is used to illustrate differences between various groups. The significance of individual principal components (PC) was calculated by numerical simulation using a program written with Matlab (Mathworks, Inc., Natick, MA). The program calculates a PCA for simulated random data 1000 times, retaining the percentage of variance explained by the first PC. If the percentage variance explained by the first PC of the actual data is larger than 95% of the values obtained for random data, this PC is considered significantly large at the p ≤ 0.05 level. The second PC is tested similarly, by considering the percentage of remaining variance explained, and comparing it to the comparable value for random data. The first two principal components (PC) were visualized in two-dimensional plots (PC1 vs. PC2).

Differences in heart tissue gene expression and differences in PBMC gene expression were assessed by ANOVA. A false discovery rate (FDR) of 10% [30], a present call (Pcall) of at least 50% in either of the two groups being compared, and a fold change (FC) of at least 3 was required to declare a probeset as differentially expressed. In heart tissue, strain effect was defined in native hearts as the log-ratio of expression levels of native hearts (BN) from allogeneic transplants to that of native hearts (DA) from isogeneic transplants. Rejection effect was defined in transplanted hearts as the log-ratio of allografts (DA) to isografts (DA) expression levels. Probesets manifesting differential expression attributable to strain, rejection, or both were then identified for heart tissue [Additional file 1]. For example, if all 3 criteria (FDR ≤ 10%, FC ≥ 3, Pcall ≥ 50% in either group being compared) were met for the native heart log-ratio, but not for the transplanted heart log-ratio then the differential expression for that probeset would be considered attributable to strain. In PBMC, the strain effect was defined by the log-ratio of expression levels for untransplanted BN rats over untransplanted DA rats. Due to the requirements of the experimental design (transplanted hearts were always of strain DA) the rejection effect in PBMC could not be separately measured (isogeneic transplants were hosted by DA rats and allogeneic transplants were hosted by BN rats). Therefore, in transplanted animals, the combined strain plus rejection effects were defined in PBMC as the log-ratio of expression levels for allograft recipients (BN) over isograft recipients (DA). Thus, probesets manifesting differential expression attributable to strain in untransplanted animals, strain plus rejection in transplanted animals or both were identified for PBMC [Additional file 2]. For example, in PBMC, if all 3 criteria (FDR ≤ 10%, FC ≥ 3, Pcall ≥ 50% in either group being compared) were met for the untransplanted log-ratio, but not for the transplanted log-ratio then the differential expression for that probeset would be considered attributable to strain.

In PBMC, to arrive at a list of genes which demonstrate a rejection effect independent of animal strain, we further analyzed the "strain plus rejection effect" list. PBMC genes whose "strain plus rejection" effect was 3-fold larger (up or down) than the strain effect alone and met a post-hoc unadjusted P ≤ 0.01 criterion were considered to demonstrate a rejection effect independent of animal strain (Table 1 and 2). These genes nominally associated with rejection in rat PBMC were then compared to previously identified rejection-related genes from studies of human PBMC after cardiac transplant [8,31,32]. To accomplish this, rat gene probesets were mapped to Entrez gene identifiers using standard Affymetrix annotations [33]. These were then mapped to Homologene identifiers [34] which were then mapped to the homologous human gene identifiers.

**Table 1:** Candidate genes induced by acute cellular rejection relative to the strain effect in peripheral blood mononuclear cells

**Gene Title**	**Gene Symbol**	**Entrez Gene**	**Strain+Rejection Effect FC**	**Strain Effect FC**	**Strain+Rejection /Strain FC**	**p-value**
ring finger protein (C3H2C3 type) 6 (predicted)	Rnf6_predicted	304271	0.40	0.01	40.54	1.44E-11
membrane-spanning 4-domains, subfamily A, member 7 (predicted)	Ms4a7_predicted	293744	0.91	0.03	31.66	2.07E-03
S100 calcium binding protein A9 (calgranulin B)	S100a9	94195	3.55	0.32	11.05	2.63E-03
complement component 1, q subcomponent, gamma polypeptide	C1qg	362634	4.07	0.46	8.84	7.39E-03
distrobrevin binding protein 1	Dtnbp1	641528	0.75	0.10	7.61	1.36E-04
matrix metallopeptidase 14 (membrane-inserted)	Mmp14	81707	4.22	0.59	7.19	3.83E-03
Thyroid hormone receptor associated protein 6 (predicted)	Thrap6_predicted	299905	0.27	0.04	7.09	1.25E-03
myxovirus (influenza virus) resistance 2	Mx2	286918	0.09	0.02	5.88	3.09E-05
prolylcarboxypeptidase (angiotensinase C) (predicted)	Prcp_predicted	293118	1.02	0.21	4.89	1.53E-04
2',5'-oligoadenylate synthetase 1, 40/46 kDa	Oas1	192281	1.33	0.28	4.69	1.33E-06
high mobility group box 1	Hmgb1	25459	0.68	0.17	3.92	1.21E-07
COX15 homolog, cytochrome c oxidase assembly protein (yeast)	Cox15	309391	0.87	0.23	3.87	1.92E-05
RT1 class II, locus Ba	RT1-Ba	309621	0.60	0.17	3.60	3.23E-04
complement factor B	Cfb	294257	0.91	0.27	3.40	4.27E-04
interferon induced transmembrane protein 3	Ifitm3	361673	0.89	0.27	3.22	1.86E-04
interferon-induced protein with tetratricopeptide repeats 3	Ifit3	309526	0.35	0.11	3.11	4.13E-03
granzyme B	Gzmb	171528	0.64	0.21	3.02	1.87E-04
granzyme A	Gzma	266708	0.70	0.23	3.00	4.25E-04

The p-value column is derived from a *post hoc *test that the Strain+Rejection FC/Strain FC is not equal to 1.

**Table 2:** Candidate genes suppressed by acute cellular rejection relative to the strain effect in peripheral blood mononuclear cells

**Gene Title**	**Gene Symbol**	**Entrez Gene**	**Strain+Rejection Effect FC**	**Strain Effect FC**	**Strain+Rejection /Strain FC**	**p-value**
similar to Glycophorin	LOC688972	688972	0.55	10.07	0.05	8.26E-04
pancreatic trypsin 1	Prss1	24691	0.08	0.91	0.09	3.49E-04
similar to RIKEN cDNA 1110063G11 (predicted)	RGD1311960_predicted	305095	0.09	0.97	0.10	1.09E-04
transcription factor Dp 2 (predicted)	Tcfdp2_predicted	300947	0.22	1.94	0.11	2.53E-03
Translocase of inner mitochondrial membrane 9 homolog (yeast)	Timm9	171139	2.20	17.91	0.12	1.19E-03
lectin, galactose binding, soluble 5	Lgals5	25475	0.18	1.36	0.13	5.55E-04
immunoglobulin heavy chain 1a (serum IgG2a)	Igh-1a	299352	0.46	3.21	0.14	2.19E-03
selenium binding protein 2	Selenbp1	140927	0.20	1.24	0.16	7.24E-03
SNF1-like kinase	Snf1lk	59329	3.31	18.46	0.18	7.67E-03
pre-eosinophil-associated ribonuclease-2	LOC474169	474169	1.15	4.82	0.24	5.67E-05
2,3-bisphosphoglycerate mutase	Bpgm	296973	0.27	1.04	0.26	9.24E-03
unc-5 homolog C (C. elegans)	Unc5c	362049	1.11	3.42	0.32	1.01E-03

The p-value column is derived from a *post hoc *test that the Strain+Rejection FC/Strain FC is not equal to 1.

Gene lists were analyzed with Ingenuity^® ^Systems Pathway Analysis (Ingenuity^® ^Systems, Redwood City, California) [35] and with the Database for Annotation, Visualization and Integrated Discovery (DAVID; NIAID, NIH) [36]. Comparative analyses for enrichment of canonical pathways, Gene Ontology terms and Kyoto Encyclopedia of Genes and Genomes (KEGG) pathways were performed between each pair of lists. Each test of enrichment is a two-sided Fisher's exact test.

## Results

### Histological Evaluation

Hematoxylin and eosin stains were performed on transplanted hearts procured on post-transplant day 6. Allografts were characterized by diffuse inflammation and necrosis consistent with ISHLT grade 3 R. Isografts exhibited minimal histological changes consistent with ISHLT grade 0 R.

### A Principal Component Analysis of Microarray Results

**Figure 2:**
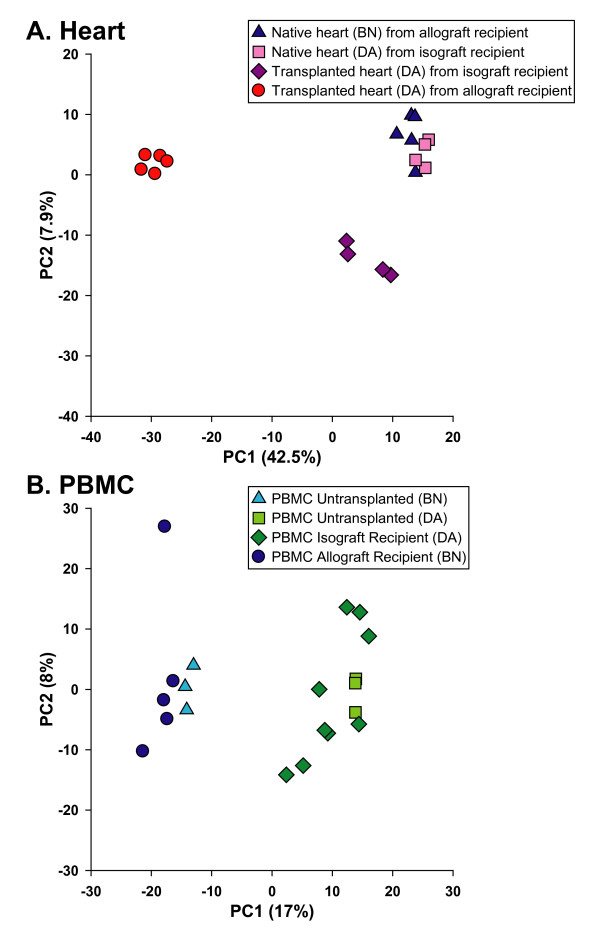
**Principal component (PC) analysis**. **(A) **Depicts variability in gene expression of native hearts from allograft (BN) and isograft recipients (DA), and of transplanted hearts from allograft (DA) and isograft recipients (DA). Principal component 1 (PC1) on the x-axis, and PC2 on the y-axis, accounted for 42.5% (p < 0.05), and 7.9% of total variability in gene expression, respectively. The samples visually separate into 3 main groups based on their immunological status: (1) rejecting transplanted hearts from allograft recipient (DA allograft); (2) non-rejecting transplanted hearts from isograft recipient (DA isograft); (3) and native hearts (DA and BN). **(B) **Depicts variability in gene expression of peripheral blood mononuclear cells (PBMC) from transplanted and untransplanted animals. Transplanted and untransplanted animal samples were processed in two batches, and the results as shown were batch-corrected for this nuisance factor. PC1 (x-axis) and PC2 (y-axis) accounted for 17% (p < 0.05) and 8% of the total variability in gene expression, respectively. Samples visually separate into 2 main groups: (1) PBMC from untransplanted DA animals and isograft recipients (DA); and (2) PBMC from untransplanted BN animals and allograft recipients (BN). The separation of animals primarily into two groups based on strain, and not ACR, indicates that most of the gene expression variability in PBMC during rejection was due to strain.

A PCA in essence creates metagene projections that define major patterns in a data set. The first component explains the largest amount of variability followed in order by the 2^nd^, 3^rd^, and 4^th ^components, *etc*. Applying a PCA to the microarray results from native and transplanted hearts provided a visual representation of the sources of variability in this experiment. PC1 encompassed a significantly large proportion (42.5%, p < 0.05) of the experimental variability and largely depicted the effect of cardiac rejection (Figure 2A). Allograft hearts separate widely from isograft hearts primarily along the PC1 axis. PC2 encompassed the next largest source of the experimental variability (7.9%), predominantly attributable to surgical effect of transplantation. Isografts separated from *in situ *native hearts along PC2. The native heart group consists of hearts from 2 different rat strains (BN and DA) which were histologically normal whether or not the animal was an allograft or isograft recipient. Native hearts remained closely grouped along both PC1 and PC2. This suggests that animal strain has little or no effect on gene expression in heart tissue.

In contrast to heart tissue, PBMC, which are mostly T-lymphocytes, displayed a gene expression pattern dominated by strain effects that obscured any contribution from cardiac rejection. An initial analysis comparing animals with rejection (allograft recipients; BN rats) to those without rejection (isograft recipients; DA rats) indicated broad differences in gene expression and the possibility of multiple, strong biomarkers that could distinguish these phenotypes (Figure 2B). Allograft and isograft recipients separated clearly from each other along the PC1 axis. However, the addition of untransplanted animals to this analysis revealed that these transcriptomic differences were almost entirely attributable to rat strain rather than cardiac rejection. Note that expression profiles from untransplanted BN and DA rats similarly separate from each other along the PC1 axis (17% of the experimental variability; p < 0.05). These untransplanted rats grouped closely with their respective strain of transplanted rats whether or not the animals had ACR (Figure 2B). PC2 was not significant and did not further resolve these groups.

### Strain Effects on the Expression Profiles of Heart Tissue and PBMC in ACR

**Figure 3:**
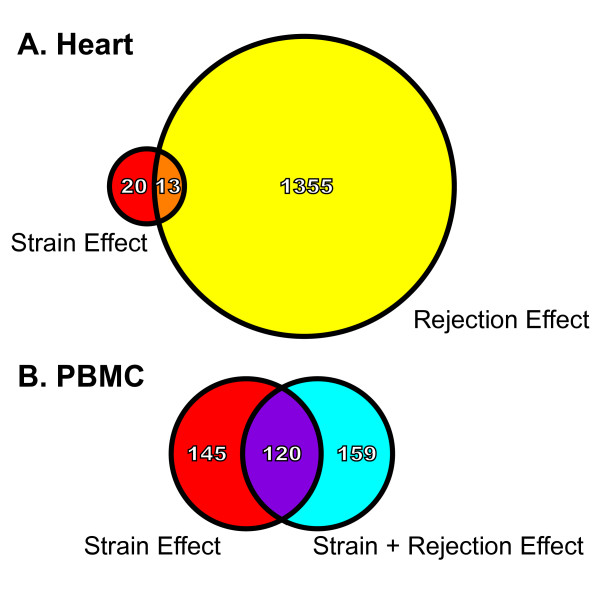
**Venn diagram representation of the union of probeset lists**. **(A) **Depicts the union of the probeset list for strain effect (native hearts) and the probeset list for rejection effect (transplanted hearts) in heart tissue. Of the 13 overlapping probesets (13 unique genes), the direction of gene expression change was concordant for 11. **(B) **Depicts the union of the probeset list for strain effect (untransplanted animals) and the probeset list for strain + rejection effect (transplanted animals) in PBMC. Of 120 overlapping probesets, the direction of gene expression change was concordant for 119. Figures 5 and 7 are color coded as defined by these Venn diagrams.

**Figure 4:**
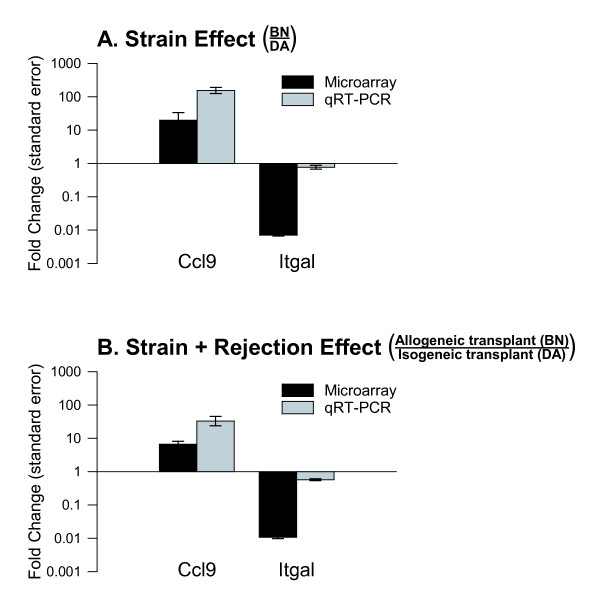
**Validation of microarray results for genes similarly affected by strain irrespective of acute cardiac rejection**. Two inflammatory response genes, Ccl9 and Itgal, were selected from the peripheral blood mononuclear cell (PBMC) overlap category shown in Figure 3B. Microarray and qRT-PCR results are from the same animals. **(A) **Strain effect in PBMC shown as the fold change in gene expression comparing untransplanted BN and DA rats. **(B) **Strain plus rejection effects in PBMC shown as the fold change in gene expression comparing allogeneic transplant recipients (BN) and isogeneic transplant recipients (DA). Ccl9 expression was higher and Itgal was lower in BN compared to DA animals irrespective of transplant status across both platforms.

Next, differentially expressed probesets between experimental groups were identified using standardized selection criteria (see Methods) to assess the relative impact of rat strain and ACR at the transcript level. In heart tissue, the number of strain-related differentially expressed probesets (33; BN vs. DA native hearts) was small compared to the much larger number of differentially expressed rejection-related probesets (1368; DA allograft vs. DA isograft). Furthermore, of the 33 strain-related probesets in heart tissue, only 13 (representing 13 uniquely named genes) were also associated with rejection; a very small fraction (<1%) of all rejection-related differentially expressed probesets in heart tissue (Figure 3A) [Additional file 1]. In contrast, for PBMC the number of differentially expressed probesets was very similar among untransplanted (265; BN vs. DA) and transplanted animals (279; BN strain, allograft recipient vs. DA strain, isograft recipient). Importantly, of the 265 strain-related differentially expressed probesets in untransplanted animals, 120 (45%) also met criteria for differential expression in transplanted animals (Figure 3B) [Additional file 2], a substantial overlap. Two inflammatory response genes (Ccl9, Itgal) were selected from the PBMC overlap category for qRT-PCR. Ccl9 expression was much higher while Itgal was lower in BN compared to DA animals, irrespective of transplant status, across both platforms (Figure 4A – B).

**Figure 5:**
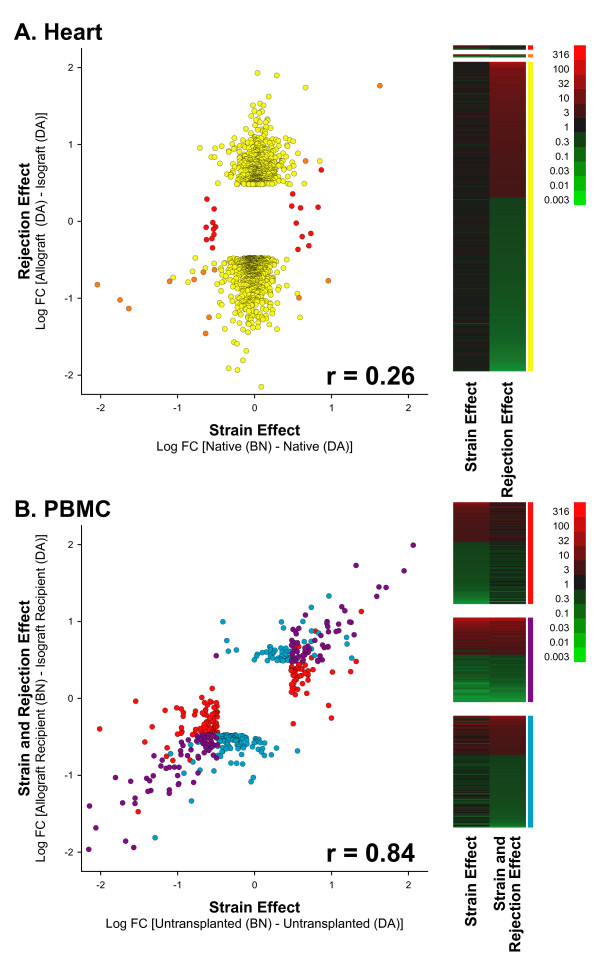
**Bivariate plots and heat maps of differentially expressed probesets**. **(A) **Relative gene expression in native (strain effect) and transplanted hearts (rejection effect) are plotted on the x and y-axes, respectively in a base 10 log scale. Each circle represents one probeset. Probesets are colored to depict group membership as defined by the Venn diagram shown in Figure 3A. Strain and rejection effects are not highly correlated (R = 0.26). Differences in gene expression due to rejection are minimally confounded by differences in gene expression due to strain in heart tissue. In the heat map, red indicates over-expression and green indicates under-expression. From the heat map, it is visually evident that strain and rejection have dissimilar gene expression patterns. **(B) **Relative gene expression in the PBMC of untransplanted (strain effect) and transplanted (strain + rejection effect) animals are plotted on the x and y-axes, respectively, in a base 10 log scale. Each circle represents one probeset. Probesets are colored to depict group membership as defined by the Venn diagram shown in Figure 3B. Strain effects in both the absence and presence of rejection are highly correlated (R = 0.84). Differences in gene expression during rejection are unapparent compared to the large background differences attributable to animal strain. In the heat map, red indicates over-expression and green indicates under-expression. From the heat map it is visually evident that strain in the absence or presence of rejection is the predominant gene expression pattern. For both (A) and (B), certain probesets appear near each other but are placed in different categories as indicated by color coding. The categorization of probesets was based on the contribution of additional variables not apparent in these scatter plots (see Methods).

To better visualize the extent of discordance or concordance in expression profiles within each tissue, bivariate plots and heat maps were generated using the union of all probesets differentially expressed in heart tissue or the union of all probesets differentially expressed in PBMC (Figure 5). This figure is color coded to track the various probeset groups as defined by the Venn diagrams shown in Figure 3. Strain and rejection effects in heart tissue were found to be highly discordant at the level of individual probesets (Figure 5A, **scatter plot**; r = 0.26), again indicating that any confounding effects of strain in heart tissue are likely to be small. As clearly seen in the adjacent heat map (Figure 5A), most probesets had very different expression patterns comparing the strain effect to the rejection effect.

**Figure 6:**
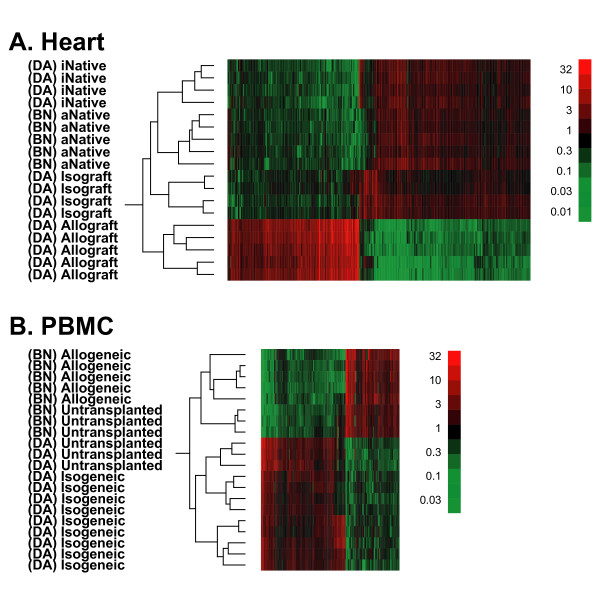
**Hierarchical clustering of the union of the probeset lists**. Two-way hierarchical clustering of samples and differentially expressed probesets within **(A) **heart tissue from native and transplanted hearts from isogeneic (i) and allogeneic (a) transplants; and **(B) **peripheral blood mononuclear cells (PBMC) from untransplanted animals and isogeneic and allogeneic transplants. Rows represent microarray chips and columns represent probesets. Red indicates over-expression and green indicates under-expression. The dendrogram illustrates how the samples segregate into groups. For heart tissue, samples segregate mainly based on the presence and absence of rejection. For PBMC, samples segregate mainly based on strain irrespective of rejection or transplant status.

In contrast to heart tissue, comparison at the probeset level of strain effect to the strain plus rejection effect in PBMC showed a high concordance (Figure 5B, **scatter plot**; r = 0.84). Both overlapping and non-overlapping probesets (Figure 3B), display very comparable expression patterns (Figure 5B, heat map). These results demonstrate that rat strain has a large effect on the transcriptomic profile of leukocytes and has the potential to overwhelm the more subtle signature of ACR. Next, the heart tissue and PBMC probeset lists were used to perform an unsupervised cluster analysis of their respective microarrays (Figure 6). For heart tissue (Figure 6A), rejecting hearts (allografts) formed a distinct group as did nonrejecting hearts (isografts and native hearts). The latter then further divided into isografts and native hearts, followed by the sorting of native hearts into those from BN rats and DA rats (allogeneic and isogeneic transplants, respectively). In contrast, PBMC microarrays primarily divided by strain effect (Figure 6B).

### Strain Effects on Pathways and Networks Potentially Relevant to ACR Pathogenesis

**Figure 7:**
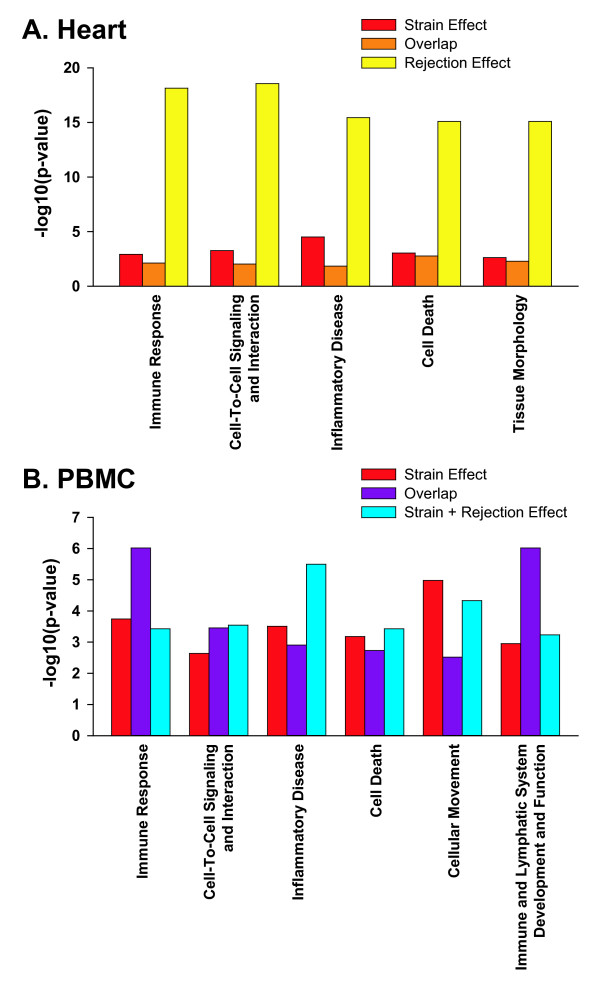
**Thematic analysis of differentially expressed genes**. **(A) **Pathway analysis in heart tissue of differentially expressed genes associated with strain effect, or rejection effect or overlap region. **(B) **Pathway analysis of differentially expressed genes in peripheral blood mononuclear cells (PBMC) associated with strain alone or strain plus rejection or both (overlap region). Genes tested are grouped and color coded as defined by the Venn diagrams shown in Figure 3. For each graph, the threshold for significance is 1.3 representing – log 0.05 (unadjusted p = 0.05). In heart tissue, the over-represented functional categories such as immune response and cell death were more strongly associated with rejection than with the strain effect. Conversely in PBMC, strain effect was strongly linked to pathways indicative of immune response and cell death both in the presence and absence of rejection. Results based on an Ingenuity^® ^Systems Pathway Analysis (see Methods).

Thematic analysis is routinely applied to link microarray results with pathways, networks, and various biological functions. This approach is considered robust in that results are relatively resistant to arbitrary changes in the methods and rules used to generate lists of differentially expressed genes [37]. As such, thematic analyses often play a large role in determining the final interpretation of expression profiles. Therefore, strain effects were examined using Ingenuity^® ^Systems Pathway Analysis (Ingenuity^® ^Systems, Redwood City, California) [35] for the presence of thematic signatures that might be misconstrued as the consequences of ACR. The rejection effect in heart tissue was strongly associated with many expected themes including immune response, cell death, and tissue morphology (Figure 7A). In contrast, the strain effect in heart tissue was only weakly associated with these gene signatures (unadjusted significance threshold p < 0.05). For PBMC, however, strain effects alone produced very significant "hits" in several biologically plausible ACR pathways including immune response, cell death, and cellular movement. Importantly, the magnitudes of the strain-only effects on these pathways were similar to those seen in animals with rejection (Figure 7B). Likewise, another analysis using DAVID (NIAID, NIH) [36] also strongly associated the strain effect with immune defense, a thematic signature consistent with ACR.

### Genes Associated with ACR in PBMC

**Figure 8:**
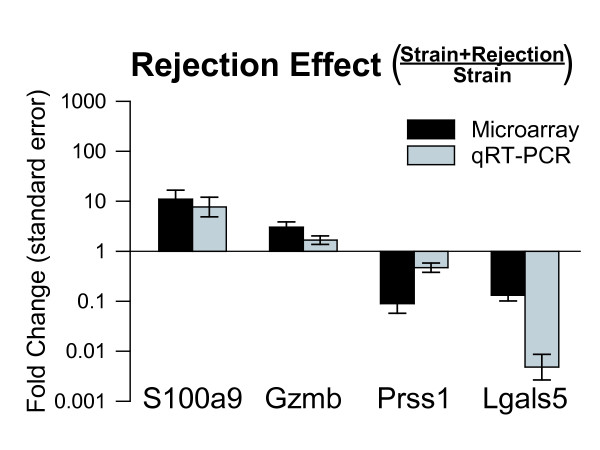
**Validation of microarray results for genes showing a rejection effect independent of animal strain**. Two genes, S100a9 and Gzmb, induced by rejection (Table 1), and 2 genes, Prss1 and Lgals5, suppressed by rejection (Table 2) in peripheral blood mononuclear cells (PBMC) were selected for qRT-PCR. Microarray and qRT-PCR are from the same animals. The rejection effect in PBMC was defined as the strain plus rejection effect divided by the strain effect. Rejection effects on these 4 genes as measured by microarray and qRT-PCR were concordant.

Even though the strain effect was larger than the ACR effect in PBMC, attempting to account for the strain effect identified 30 candidate genes potentially associated with ACR. Of these 30 candidates, 18 were induced (Table 1) and 12 were suppressed (Table 2) in rejection relative to the strain alone effect. Four of these genes, two induced (S100a9, Gzmb) and two suppressed (Prss1, Lgals5) by rejection were selected for qRT-PCR. The qRT-PCR and microarray fold change ratios (Strain + Rejection Effect/Strain Effect) for these 4 genes were all directionally concordant (Figure 8).

**Figure 9:**
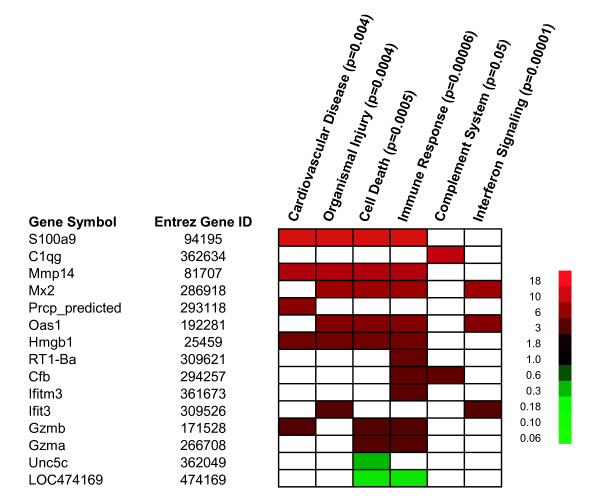
**Thematic analysis of candidate genes associated with rejection in peripheral blood mononuclear cells (PBMC)**. Pathway analysis of genes differentially expressed in PBMC in response to acute cardiac rejection (**Table 1 and 2**) after accounting for strain effects. Red indicates over-expression and green indicates under-expression. Results based on an Ingenuity^® ^Systems Pathway Analysis.

A thematic analysis (Figure 9) of these 30 genes (Ingenuity^® ^Systems, Redwood City, California) [35] connect them to organismal injury, cell death, immune response, the complement system, and interferon signaling, pathways previously associated with allograft rejection. Similarly, a high stringency analysis using DAVID (NIAID, NIH) [36] also found these genes to be strongly associated with the immune response and complement activation.

Of the 30 candidate genes, 22 could be mapped to homologous human genes. These 22 homologues were compared against a list of 97 uniquely named genes compiled from 3 human studies investigating differential PBMC gene expression in cardiac ACR [8,31,32]. Two genes, calgranulin B (S100a9; S100 calcium binding protein A9) and granzyme B (Gzmb) were present on both lists. In rats with ACR, S100a9 was induced 11 fold and Gzmb was induced 3 fold relative to the strain alone effect.

## Discussion

Relatively large expression differences were detected in PBMC between two strains of rats commonly used in models of organ transplantation. Conversely, only a small number of differentially expressed genes in heart tissue were related to rat strain. The larger effect of strain on gene expression in PBMC compared to heart tissue may in part reflect the abundance of strain-specific, self-recognition molecules that are expressed by lymphocytes. As such, strain specifically affected genes involved with the immune response, cell motility, and cell death, functional categories that might be misconstrued as ACR-related. Moreover, the magnitude of the strain effect in PBMC (FC ≥ 3 for 265 probesets) substantially obscured the gene expression signature of rejection. Potential markers specific to ACR could only be identified after measuring and accounting for the strain effect. Similar variability attributable to genotypic heterogeneity has also been documented in human populations [16,17,38] and therefore has major implications for experimental design and power calculations in patient biomarker studies.

Approximately 85 to 95% of human genetic variation is attributable to individual heterogeneity within a population while the remaining 5 to 15% can be ascribed to differences between populations [16,18]. In addition to external influences and epigenetic factors, this background genetic variation contributes to differences in gene expression [15-17] that may add unwanted noise to results from high – throughput methodologies such as microarrays. Genotypic effects in human PBMC were found to significantly alter the expression of more than 300 transcripts [38]. Major histocompatibility complex-associated and interferon-regulated genes were among those most affected by genotype in human PBMC preparations. Investigations performed in inbred animals reduce this source of variability and thereby serve as a "best case" scenario or "proof of principle." However, genetic background may still negatively impact the results and interpretation of animal experiments, a well recognized concern in gene targeting studies [39]. At particular risk are investigations that require hybrid animals or multiple animal strains.

Natural variation in gene expression has been well documented among laboratory strains of fruit flies [19] and mice [20-22,40]. Expression profiling of brain [20,21], spleen [21], and liver [22] have determined that 1 to 3% of mouse transcripts are significantly affected by animal strain. In addition to these baseline effects, the gene expression response to seizure was shown to be significantly different comparing the brains of two inbred strains of mice [20]. Even more relevant to immunity and transplant medicine, bone marrow derived macrophages from 5 mouse strains displayed unique transcriptional phenotypes in response to lipopolysaccharide challenges [40]. Allogeneic animal transplant models typically employ two immunologically distinct strains to serve as donor and recipient. Therefore background genotypic effects on transcript abundance have a real potential to confound the search for biomarkers and new therapeutic targets. In the current study, the impact of rat strain on PBMC gene expression was unexpectedly large and primarily affected transcripts associated with the immune response, effects that could be misinterpreted as ACR-related. These results underscore the importance of experimental designs and analytical approaches in expression profiling studies that control for strain effects in animal models and genotypic heterogeneity in patient populations.

Measuring and then adjusting *post hoc *for large strain effects on gene expression identified 30 genes with the potential to be differentially regulated during rejection in circulating PBMC. A thematic analysis of this list suggested biological plausibility. However, homologues of only two of these genes, S100 calcium binding protein A9 and granzyme B have been previously linked to cardiac rejection in humans [8,32]. Two S100 like binding proteins, myeloid related protein 8 (MRP8) and MRP14, have been shown to be increased in the serum of patients relatively early in acute renal allograft rejection [41]. Granzyme B has also been found to be over-expressed in the peripheral blood of patients with acute renal allograft rejection [42]. Another potentially rejection-related gene from the current study, high mobility group box 1 (Hmgb1) has been investigated in a previous animal study of acute rejection [43]. In a murine cardiac transplant model, Hmgb1 expression was significantly increased in allogeneic compared to isogeneic transplants. Increased expression of Hmgb1 in allografts was associated with active secretion of Hmgb1 by infiltrating immune cells. Blockade of extracellular Hmgb1 significantly delayed acute allograft rejection [43].

This study has several limitations. PBMC from transplanted animals differed by both transplant type (isogeneic vs. allogeneic) and animal strain. If strain and ACR interacted in a non-additive manner then the selection of rejection-related genes might have been unreliable. These genes require confirmation using an experimental design that avoids the need for cross strain comparisons. Likewise, it would be prudent to validate any results in more than one rat strain. Robust genes that identify rejection within multiple rat strains might be more likely to serve as reliable biomarkers in patients with their inherent heterogeneity. Another potential limitation of our analysis was the assumption that systemic effects of ACR on native hearts were minimal and that expression differences in native hearts were almost entirely due to strain. Nonetheless, the heart tissue strain effect, examined only in native hearts, was relatively small. Finally, in our study and others, some gene expression differences attributed to ACR in heart tissue may reflect the detection of strain-associated gene expression attributable to recipient lymphocytes that have infiltrated the donor allograft.

Despite these limitations, our results have implications for recent efforts to identify biomarkers of ACR in peripheral blood [8,31,32,44-46]. Human genetic variation has a substantial impact on gene expression independent of exogenous factors and conditions [15-17,38]. Notably, the influence of genetic variation on transcript abundance appears to be particularly strong in cells of the immune system [17,38]. Baseline genetic differences between human transplant recipients may make it difficult to identify a universally applicable set of biomarkers for noninvasively detecting acute cellular rejection. Complex interactions between genetic background (polymorphisms), comorbidities, and immunosuppressive regimens may further degrade the performance characteristics of biomarkers in this heterogeneous patient population.

## Conclusion

Tissue specific differences in strain between donor and recipient animals can confound gene expression profiles in animal models of ACR. In heart tissue, there is a very modest strain effect which shares little in common with the effects of rejection in transplanted hearts. In PBMC, there is a substantial strain effect in untransplanted animals which shares a great deal in common with the combined effects of strain and rejection in transplanted animals. When performing animal gene expression studies, animal strain effects should be considered and accounted for in the design and analysis of the study. Given the magnitude of strain-related effects in PBMC preparations, the most prudent approach would be to avoid cross-strain comparisons in leukocyte studies of transplant rejection. These findings may also have implications for gene expression studies in diverse, genetically heterogeneous patient populations undergoing transplantation.

## Authors' contributions

KJD and PCM participated in study design, implementation, and data analysis. HC participated in study implementation. SJK, KJN, JJB, and PJM performed the bioinformatics and statistical analysis. CL and SMT processed samples for microarrays. SA performed the quantitative PCR. JDS assessed and graded histopathology of all tissue samples. RLD participated in study design, and data analysis. MAS conceived of the study, and participated in its design, coordination and analysis. All authors contributed towards the drafting of the manuscript and read and approved the final manuscript.

## Supplementary Material

Additional file 1 **Heart Tissue: Alterations in Gene Expression due to Strain Effect and Rejection Effect (color coded based on Venn diagram, Figure **3A**)**. This table lists probesets manifesting differential expression attributable to strain, rejection, or both for heart tissue. Differences in heart tissue gene expression were assessed by ANOVA. A false discovery rate of 10%, a present call of at least 50% in either of the two groups being compared, and a fold change of at least 3 was required to declare a probeset as differentially expressed. Click here for file

Additional file 2 **Peripheral Blood Mononuclear Cells: Alterations in Gene Expression due to Strain Effect and Strain + Rejection Effect (color coded based on Venn diagram, Figure **3B**)**. This table lists probesets manifesting differential expression attributable to strain in untransplanted animals, strain plus rejection in transplanted animals or both for peripheral blood mononuclear cells (PBMC). Differences in PBMC gene expression were assessed by ANOVA. A false discovery rate of 10%, a present call of at least 50% in either of the two groups being compared, and a fold change of at least 3 was required to declare a probeset as differentially expressed. Click here for file
