# Supplementary material for: Impact of animal strain on gene expression in a rat model of acute cardiac rejection
Source: BMC Genomics. 2009 Jun 24;10:280. doi: 10.1186/1471-2164-10-280 (PMC2711118; doi:10.1186/1471-2164-10-280)
Supplement: Additional file 1 — Heart Tissue: Alterations in Gene Expression due to Strain Effect and Rejection Effect (color coded based on Venn diagram, Figure 3A). This table lists probesets manifesting differential expression attributable to strain, rejection, or both for heart tissue. Differences in heart tissue gene expression were assessed by ANOVA. A false discovery rate of 10%, a present call of at least 50% in either of the two groups being compared, and a fold change of at least 3 was required to declare a probeset as differentially expressed. [file 1471-2164-10-280-S1.pdf]

Additional File 1. Heart Tissue: Alterations in Gene Expression due to Strain Effect and Rejection Effect (color coded based on Venn diagram, Figure 3A)

				Fold Change		False Discovery Rate		Average Present Call			
				Strain Effect	Rejection Effect						
Probe Set ID	Gene Title	Gene Symbol	Entrez Gene ID	Native BN / DA	Allograft / Isograft	Native BN / DA	Allograft / Isograft	Native BN	Native DA	Allograft	Isograft
**A. Strain Effect**											
1387011_at	lipocalin 2	Lcn2	170496	7.44	4.62	1.0E-01	7.9E-03	0.60	0.00	0.40	0.00
1388018_at	selectin, endothelial cell	Sele	25544	6.77	1.51	9.1E-02	1.9E-01	0.50	0.00	0.80	0.13
1369928_at	actin, alpha 1, skeletal muscle	Acta1	29437	5.45	0.69	2.0E-03	3.0E-01	1.00	1.00	1.00	1.00
1389673_at	Transcribed locus			5.12	0.48	1.2E-02	7.6E-03	1.00	1.00	0.80	1.00
1369152_at	protein phosphatase 3, regulatory subunit B, alpha isoform (calcineurin B, type I)	Ppp3r1	29748	4.20	0.63	2.3E-02	2.0E-01	1.00	0.00	0.00	0.00
1372060_at				4.00	1.48	5.0E-03	3.5E-01	1.00	0.00	0.40	0.00
1389185_at	Adenylate kinase 1	Ak1	24183	3.70	0.43	3.3E-03	1.7E-03	1.00	1.00	1.00	1.00
1389234_at	von Willebrand factor	Vwf	116669	3.51	0.94	3.8E-02	7.8E-01	1.00	1.00	1.00	1.00
1368128_at	phospholipase A2, group IIA (platelets, synovial fluid)	Pla2g2a	29692	3.16	2.25	4.9E-02	1.1E-01	1.00	1.00	1.00	0.63
1386881_at	insulin-like growth factor binding protein 3	Igfbp3	24484	3.07	1.56	3.3E-03	1.7E-01	1.00	1.00	1.00	1.00
1368042_a_at	high mobility group box 1	Hmgb1	25459	0.31	0.84	4.8E-02	4.8E-02	1.00	1.00	1.00	1.00
1372577_at	actin related protein 2/3 complex, subunit 4 (predicted)	Arpc4_predicted	297518	0.30	1.43	4.8E-02	3.3E-02	1.00	1.00	1.00	1.00
1369716_s_at	lectin, galactose binding, soluble 5	Lgals5	25475	0.30	0.67	5.3E-02	2.0E-01	0.00	1.00	1.00	1.00
1374429_at	Transcribed locus			0.30	0.78	3.7E-02	1.9E-01	1.00	1.00	1.00	1.00
1386922_at	carbonic anhydrase 2	Ca2	54231	0.29	0.96	2.3E-02	8.2E-01	1.00	1.00	1.00	1.00
1375343_at	Transcribed locus			0.29	0.45	1.2E-02	8.6E-03	1.00	1.00	1.00	1.00
1370886_a_at	kinesin 2	Kns2	171041	0.28	0.59	3.3E-03	8.6E-02	1.00	1.00	1.00	1.00
1382778_at	Dual specificity phosphatase 6	Dusp6	116663	0.24	1.93	7.1E-02	1.5E-02	0.00	0.88	1.00	1.00
1377114_at				0.24	0.57	3.1E-03	7.2E-02	1.00	1.00	1.00	1.00
1375519_at	globin, alpha	LOC287167	287167	0.24	0.83	5.9E-02	7.1E-01	1.00	1.00	1.00	1.00
**B. Rejection Effect**											
1368266_at	arginase 1	Arg1	29221	1.10	84.50	9.5E-01	1.7E-05	0.20	0.00	1.00	0.00
1368521_at	napsin A aspartic peptidase	Napsa	365668	1.96	78.18	2.2E-01	4.4E-05	0.00	0.00	1.00	0.00
1370493_a_at	leukocyte immunoglobulin-like receptor, subfamily B (with TM and ITIM domains), member 3	Lilrb3	361493	4.65	54.39	3.9E-02	1.1E-03	0.20	0.00	1.00	0.00
1369191_at	interleukin 6	Il6	24498	1.54	41.53	6.8E-01	1.9E-03	0.00	0.00	1.00	0.00
1375917_at	glycoprotein 49b	Gp49b	292594	1.19	33.73	9.0E-01	2.9E-03	0.00	0.00	1.00	0.25
1369877_at	CD8 antigen, alpha chain	Cd8a	24930	0.96	32.01	9.8E-01	9.8E-04	0.00	0.00	1.00	0.00
1373523_at	Fc fragment of IgG, low affinity IIIa, receptor	Fcgr3a	304966	1.33	31.88	2.2E-01	1.3E-04	0.00	0.00	1.00	0.00
1387902_a_at	similar to IG KAPPA CHAIN V-V REGION K2 PRECURSOR	LOC500180	500180	1.01	26.98	9.9E-01	2.0E-04	0.00	0.00	1.00	0.00
1369290_at	chemokine (C-C motif) receptor 5	Ccr5	117029	1.22	26.71	8.7E-01	1.3E-03	0.00	0.00	1.00	0.00
1368519_at	serine (or cysteine) peptidase inhibitor, clade E, member 1	Serpine1	24617	1.27	26.62	7.4E-01	1.1E-03	0.00	0.00	1.00	0.25
1370628_at	granzyme B	Gzmb	171528	1.16	25.43	9.0E-01	5.1E-03	0.00	0.00	1.00	0.00
1388046_at	integrin alpha M	Itgam	25021	0.91	23.20	8.2E-01	4.4E-05	0.00	0.00	1.00	0.00
1371079_at	Fc receptor, IgG, low affinity IIb	Fcgr2b	289211	1.49	21.15	8.2E-01	1.3E-04	0.00	0.00	1.00	1.00
1387472_at	CD3 antigen delta polypeptide	Cd3d	25710	1.12	21.15	9.4E-01	7.5E-04	0.00	0.00	1.00	0.00
1387835_at	interleukin 1 receptor antagonist	Il1rn	60582	1.06	20.57	8.9E-01	6.8E-03	0.00	0.00	0.60	0.00
1376062_at	syndecan 1	Sdc1	25216	1.50	20.33	6.5E-01	1.9E-03	0.00	0.00	1.00	0.00
1367581_a_at	secreted phosphoprotein 1	Spp1	25353	1.18	19.21	7.0E-01	6.2E-04	0.00	0.00	1.00	1.00
1368965_at	solute carrier family 16 (monocarboxylic acid transporters), member 3	Slc16a3	80878	1.41	19.20	6.2E-01	1.3E-04	0.00	0.00	1.00	0.00
1372989_at	hypothetical LOC308081	LOC308081	308081	1.02	17.60	9.7E-01	2.5E-04	0.00	0.00	0.90	0.00
1390797_at	lymphocyte cytosolic protein 2	Lcp2	155918	1.43	16.85	3.6E-01	2.5E-03	0.00	0.00	1.00	0.00
1370902_at	aldo-keto reductase family 1, member B8	Akr1b8	286921	1.63	16.22	2.8E-01	2.8E-04	0.40	0.00	1.00	0.25
1387160_at	potassium voltage-gated channel, Isk-related subfamily, member 3	Kcne3	63883	0.78	15.78	9.1E-01	1.5E-04	0.00	0.00	0.80	0.00
1398275_at	matrix metallopeptidase 9	Mmp9	81687	1.24	15.60	7.8E-01	1.4E-04	0.00	0.00	0.80	0.00
1370613_s_at	UDP glycosyltransferase 1 family, polypeptide A1	Ugt1a1	113992	1.11	15.38	9.3E-01	7.8E-04	0.00	0.00	1.00	0.00
1370080_at	heme oxygenase (decycling) 1	Hmox1	24451	1.29	14.78	4.9E-01	1.9E-04	1.00	1.00	1.00	1.00
1370090_at	lymphocyte cytosolic protein 2	Lcp2	155918	1.38	14.59	7.7E-01	1.5E-04	0.00	0.00	1.00	0.25
1383500_at	ribosome binding protein 1 homolog 180kDa (dog) (predicted)	Rrbp1_predicted	311483	0.91	13.91	9.6E-01	1.4E-03	0.00	0.00	0.60	0.00
1374779_at	Coagulation factor XIII, A1 subunit	F13a1	60327	1.40	13.71	2.8E-01	6.4E-05	1.00	0.63	1.00	1.00
1370904_at	major histocompatibility complex, class II, DM alpha	Hla-dma	294274	1.32	12.67	5.8E-01	1.3E-04	1.00	0.38	1.00	1.00
1371913_at	transforming growth factor, beta induced	Tgfbi	116487	1.01	12.42	9.9E-01	1.1E-04	1.00	1.00	1.00	1.00
1387017_at	squalene epoxidase	Sqle	29230	1.03	12.33	9.4E-01	2.2E-03	0.00	0.00	1.00	0.00
1370885_at	cathepsin Z	Ctsz	252929	1.14	12.13	6.8E-01	3.6E-04	1.00	1.00	1.00	1.00
1368930_at	potassium intermediate/small conductance calcium-activated channel, subfamily N, member 4	Kcnn4	65206	0.55	12.01	4.9E-01	4.1E-04	0.00	0.00	1.00	0.00
1368134_a_at	interleukin 4 receptor, alpha	Il4ra	25084	2.57	11.97	4.9E-01	6.5E-03	0.00	0.00	1.00	0.00
1388436_at	small nuclear ribonucleoprotein polypeptide A	Snrpa	292729	1.24	11.95	8.6E-01	4.8E-04	0.00	0.00	1.00	0.00
1369964_at	coronin, actin binding protein 1A	Coro1a	155151	1.73	11.63	1.0E-01	2.0E-04	0.00	0.00	1.00	0.13
1387396_at	hepcidin antimicrobial peptide	Hamp	84604	1.01	11.55	9.9E-01	3.5E-04	1.00	1.00	1.00	1.00
1371209_at	RT1 class I, CE5	RT1-CE5	309607	1.92	11.50	1.4E-01	2.0E-04	1.00	0.75	1.00	1.00
1377698_at	tumor necrosis factor receptor superfamily, member 5	Tnfrsf5	171369	0.92	11.47	8.9E-01	2.4E-03	0.00	0.00	0.80	0.00
1370483_at	similar to transmembrane NK cell receptor 2B4	LOC679046	679046	0.90	11.34	9.0E-01	1.4E-03	0.80	0.75	1.00	0.63
1373889_at	similar to Cd300D antigen	LOC498022	498022	1.06	11.34	9.1E-01	3.7E-04	1.00	1.00	1.00	1.00
1371447_at	placenta-specific 8 (predicted)	Plac8_predicted	360914	2.89	11.33	1.5E-01	4.3E-04	1.00	0.88	1.00	1.00
1377163_at	inhibin beta-B	Inhbb	25196	1.43	11.25	8.6E-01	4.7E-04	0.10	0.00	1.00	0.00
1367705_at	glutaredoxin 1 (thioltransferase)	Glrx1	64045	0.71	11.24	2.0E-01	2.2E-04	1.00	1.00	1.00	1.00
1376100_at	tubulin, beta 6	Tubb6	307351	1.05	11.18	9.5E-01	1.9E-04	0.20	0.00	1.00	1.00
1370964_at	argininosuccinate synthetase	Ass	25698	1.48	11.15	5.5E-01	1.4E-04	0.20	0.00	1.00	0.00
1369294_at	bone marrow stromal cell antigen 1	Bst1	81506	1.09	11.11	8.8E-01	2.8E-03	0.00	0.00	1.00	0.00
1374730_at	Thioredoxin reductase 3 (predicted)	Txnrd3_predicted	297437	1.12	11.10	6.5E-01	2.3E-04	1.00	1.00	1.00	1.00
1374775_at	antigen identified by monoclonal antibody Ki-67 (predicted)	Mki67_predicted	291234	2.06	11.09	4.7E-01	1.1E-02	0.00	0.00	1.00	0.50
1373932_at	Transcribed locus			3.21	10.89	2.5E-01	7.2E-04	1.00	0.25	1.00	1.00
1369665_a_at	interleukin 18	Il18	29197	1.41	10.78	4.9E-01	8.5E-05	0.00	0.00	1.00	0.63
1386998_at	aldolase C	Aldoc	24191	1.18	10.49	7.9E-01	2.7E-04	0.00	0.00	0.60	0.00

				Fold Change		False Discovery Rate		Average Present Call			
				Strain Effect	Rejection Effect						
Probe Set ID	Gene Title	Gene Symbol	Entrez Gene ID	Native BN / DA	Allograft / Isograft	Native BN / DA	Allograft / Isograft	Native BN	Native DA	Allograft	Isograft
1386052_at	Transcribed locus			0.82	10.48	7.8E-01	3.2E-03	0.00	0.00	1.00	0.00
1368674_at	liver glycogen phosphorylase	Pygl	64035	1.73	10.39	7.0E-01	3.7E-04	0.00	0.00	1.00	0.00
1368530_at	matrix metallopeptidase 12	Mmp12	117033	0.92	10.35	9.1E-01	6.9E-04	0.00	0.00	1.00	0.75
1370869_at	branched chain aminotransferase 1, cytosolic	Bcat1	29592	1.59	10.35	6.1E-01	6.4E-04	0.00	0.00	1.00	0.25
1388596_at	coactosin-like 1 (Dictyostelium) (predicted)	Cotl1_predicted	361422	1.11	10.29	7.8E-01	1.9E-04	0.40	0.00	1.00	1.00
1368518_at	CD53 antigen	Cd53	24251	1.24	10.15	4.2E-01	4.8E-04	1.00	1.00	1.00	1.00
1387675_at	Cystatin 10 (chondrocytes) (predicted)	Cst10_predicted	366219	0.52	10.08	4.8E-01	1.1E-04	0.00	0.00	1.00	0.50
1368482_at	B-cell leukemia/lymphoma 2 related protein A1	Bcl2a1	170929	1.13	9.99	7.2E-01	5.2E-04	0.00	0.00	1.00	0.75
1370382_at	RT1 class II, locus Bb	RT1-Bb	309622	1.08	9.98	9.7E-01	1.3E-03	0.00	0.00	1.00	0.00
1368490_at	CD14 antigen	Cd14	60350	1.29	9.75	5.3E-01	2.0E-04	0.80	0.50	1.00	1.00
1370371_a_at	CEA-related cell adhesion molecule 1	Ceacam1	287009	2.66	9.67	4.1E-01	6.7E-03	0.00	0.00	0.70	0.00
1392794_at	Transcribed locus			2.08	9.54	3.6E-01	1.1E-04	0.00	0.00	1.00	0.00
1398287_at	plasminogen activator, urokinase	Plau	25619	0.65	9.53	5.2E-01	6.2E-03	0.00	0.00	1.00	0.00
1368541_at	embigin	Emb	114511	0.73	9.48	4.6E-01	1.5E-04	0.20	0.25	1.00	0.75
1368735_a_at	transient receptor potential cation channel, subfamily V, member 2	Trpv2	29465	1.16	9.44	9.1E-01	3.1E-03	0.00	0.00	1.00	0.00
1372886_at	transforming, acidic coiled-coil containing protein 3	Tacc3	360962	0.38	9.38	5.2E-01	2.8E-03	0.00	0.00	1.00	0.00
1387125_at	S100 calcium binding protein A9 (calgranulin B)	S100a9	94195	1.67	9.38	6.6E-01	5.4E-03	1.00	1.00	1.00	1.00
1393280_at	lymphocyte antigen 86 (predicted)	Ly86_predicted	291359	0.99	9.36	9.9E-01	3.2E-04	0.00	0.00	1.00	0.88
1388255_x_at	RT1 class I, CE5	RT1-CE5	309607	2.72	9.36	2.0E-01	8.2E-04	1.00	1.00	1.00	1.00
1387134_at	schlafen 3	Slfn3	114247	1.27	9.25	4.7E-01	1.3E-04	0.00	0.00	1.00	0.00
1371146_at	transcription factor EC	Tfec	26296	0.97	9.19	9.8E-01	2.7E-03	0.00	0.00	0.60	0.00
1376390_at	membrane-spanning 4-domains, subfamily A, member 11 (predicted)	Ms4a11_predicted	361735	0.97	9.09	9.5E-01	5.3E-04	0.40	0.13	1.00	1.00
1370083_at	chemokine (C-C motif) receptor 1	Ccr1	57301	0.76	9.07	8.7E-01	6.4E-04	0.00	0.00	1.00	0.00
1374200_at	solute carrier family 29 (nucleoside transporters), member 3	Slc29a3	353307	1.33	8.95	7.4E-01	1.1E-02	0.00	0.00	1.00	0.00
1373722_at	kinesin family member 20A (predicted)	Kif20a_predicted	361308	0.20	8.94	6.3E-02	2.3E-02	0.00	0.00	0.60	0.00
1367849_at	syndecan 1	Sdc1	25216	1.07	8.93	8.7E-01	4.4E-04	0.40	0.75	1.00	1.00
1370682_at	leukocyte immunoglobulin-like receptor, subfamily B (with TM and ITIM domains), member 3	Lilrb3	361493	1.70	8.89	3.1E-01	1.4E-04	1.00	0.63	1.00	1.00
1370422_at	receptor-interacting serine-threonine kinase 3	Ripk3	246240	0.85	8.87	8.0E-01	2.0E-04	0.20	0.00	1.00	0.75
1386908_at	glutaredoxin 1 (thioltransferase)	Glrx1	64045	0.31	8.86	1.9E-01	1.1E-04	0.50	1.00	1.00	1.00
1390383_at	adipose differentiation related protein	Adfp	298199	1.18	8.86	2.1E-01	9.8E-05	1.00	1.00	1.00	1.00
1387715_at	extracellular peptidase inhibitor	Expi	171059	0.56	8.85	4.9E-01	2.2E-03	0.00	0.00	0.60	0.00
1392731_at	tumor necrosis factor receptor superfamily, member 1b	Tnfrsf1b	156767	1.25	8.81	8.6E-01	3.7E-04	0.00	0.00	0.70	0.00
1373025_at	complement component 1, q subcomponent, gamma polypeptide	C1qg	362634	1.41	8.78	4.4E-01	1.6E-04	1.00	1.00	1.00	1.00
1368760_at	chemokine (C-X-C motif) ligand 2	Cxcl2	114105	1.35	8.71	8.0E-01	1.0E-02	0.00	0.00	0.90	0.00
1390195_at	pleckstrin and Sec7 domain containing 4	Psd4	311785	0.54	8.65	5.5E-01	1.6E-03	0.00	0.00	0.90	0.00
1370119_at	leucocyte specific transcript 1	Lst1	64569	1.04	8.64	9.8E-01	3.2E-03	0.00	0.00	1.00	0.00
1368280_at	cathepsin C	Ctsc	25423	1.36	8.62	2.4E-01	1.6E-03	1.00	1.00	1.00	1.00
1385047_x_at	Complement component 6	C6	24237	1.42	8.55	6.4E-01	2.3E-04	0.00	0.00	1.00	0.50
1387799_at	FXYD domain-containing ion transport regulator 2	Fxyd2	29639	1.92	8.52	1.6E-01	3.0E-04	1.00	0.75	1.00	1.00
1368558_s_at	allograft inflammatory factor 1	Aif1	29427	1.04	8.45	9.4E-01	1.8E-04	0.60	0.50	1.00	1.00
1386938_at	alanyl (membrane) aminopeptidase	Anpep	81641	1.09	8.44	8.8E-01	1.6E-04	1.00	0.75	1.00	1.00
1370882_at	major histocompatibility complex, class II, DM beta	Hla-dmb	294273	1.26	8.41	4.3E-01	1.1E-04	1.00	1.00	1.00	1.00
1370383_s_at	RT1 class II, locus Db1	RT1-Db1	294270	1.11	8.39	8.5E-01	3.7E-04	1.00	1.00	1.00	1.00
1389210_at	lymphocyte cytosolic protein 1	Lcp1	306071	1.36	8.34	2.3E-01	1.1E-04	1.00	1.00	1.00	1.00
1369029_at	phospholipid scramblase 1	Plscr1	117540	1.76	8.18	5.9E-01	1.4E-04	0.80	0.25	1.00	0.75
1388340_at	Protocadherin beta 21 (predicted)	Pcdhb21_predicted	24189	1.78	8.15	6.6E-01	1.4E-04	0.80	0.50	1.00	1.00
1398540_at	regulator of G-protein signaling 1	Rgs1	54289	0.47	8.13	5.9E-01	7.1E-03	0.00	0.00	1.00	0.50
1375010_at	CD68 antigen	Cd68	287435	0.95	8.12	9.5E-01	4.9E-04	0.00	0.00	1.00	1.00
1398390_at	similar to Small inducible cytokine B13 precursor	LOC498335	498335	1.10	8.12	9.0E-01	1.2E-03	0.60	0.75	1.00	1.00
1372665_at	phosphoserine aminotransferase 1	Psat1	293820	1.10	8.03	9.7E-01	1.5E-02	0.00	0.00	1.00	0.00
1376296_at	Phosphatidylinositol 3-kinase, regulatory subunit, polypeptide 1	Pik3r1	25513	0.64	7.97	5.7E-01	3.0E-02	0.00	0.00	0.90	0.00
1398449_at	Protein tyrosine phosphatase, non-receptor type 7	Ptpn7	246781	0.88	7.96	9.2E-01	3.2E-04	0.00	0.00	1.00	0.00
1372321_at	lymphoblastic leukemia derived sequence 1	Lyl1	304663	1.00	7.92	1.0E+00	2.5E-03	0.00	0.00	0.50	0.00
1369166_at	matrix metallopeptidase 9	Mmp9	81687	0.47	7.90	4.7E-01	7.8E-03	0.00	0.00	0.50	0.00
1377034_at	serine (or cysteine) proteinase inhibitor, clade B, member 1a	Serpinb1a	291091	1.66	7.89	1.8E-01	6.3E-05	0.00	0.00	1.00	0.25
1368332_at	guanylate nucleotide binding protein 2	Gbp2	171164	1.35	7.88	5.1E-01	3.3E-04	1.00	1.00	1.00	1.00
1374469_at	Similar to C20orf118 (predicted)	RGD1561343_predicted	502689	1.40	7.84	7.7E-01	2.9E-03	0.10	0.00	0.80	0.00
1367998_at	secretory leukocyte peptidase inhibitor	Slpi	296356	1.03	7.82	9.6E-01	2.2E-04	0.80	0.00	1.00	1.00
1390141_at	methylenetetrahydrofolate dehydrogenase (NADP+ dependent) 1-like (predicted)	Mthfd1l_predicted	361472	1.55	7.74	8.1E-01	5.7E-03	0.00	0.00	1.00	0.00
1368693_at	Gardner-Rasheed feline sarcoma viral (Fgr) oncogene homolog	Fgr	79113	1.41	7.74	2.4E-01	1.5E-04	0.60	0.00	1.00	0.25
1370987_at	sialophorin	Spn	24796	2.36	7.62	4.8E-01	2.8E-03	0.10	0.00	1.00	0.38
1371210_s_at	RT1 class Ib, locus Aw2	RT1-Aw2	24737	3.69	7.59	2.0E-01	4.0E-03	0.80	0.00	1.00	0.50
1368487_at	serine (or cysteine) proteinase inhibitor, clade B, member 2	Serpinb2	60325	0.97	7.56	9.9E-01	8.3E-03	0.00	0.00	0.80	0.00
1388809_at	sphingomyelin phosphodiesterase, acid-like 3A	Smpdl3a	294422	0.85	7.54	5.3E-01	1.4E-03	1.00	1.00	1.00	1.00
1388673_at	BCL2-like 12 (proline rich) (predicted)	Bcl2l12_predicted	361567	1.58	7.45	5.7E-01	4.9E-04	0.00	0.00	1.00	0.50
1370400_at	Cullin 2 (predicted)	Cul2_predicted	361258	1.15	7.37	9.5E-01	4.5E-03	0.00	0.00	1.00	0.00
1398256_at	interleukin 1 beta	Il1b	24494	0.90	7.36	9.3E-01	1.7E-04	0.40	0.25	1.00	0.63
1368006_at	lysosomal-associated protein transmembrane 5	Laptm5	89783	1.28	7.36	4.7E-01	1.7E-04	1.00	1.00	1.00	1.00
1388740_at	similar to cDNA sequence BC032204 (predicted)	RGD1310168_predicted	309186	1.14	7.35	9.5E-01	4.5E-04	0.00	0.00	1.00	0.25
1388924_at	angiopoietin-like 4	Angptl4	362850	0.74	7.33	7.5E-01	3.7E-04	0.10	0.00	1.00	1.00
1371360_at	N-myc downstream regulated gene 1	Ndrg1	299923	1.10	7.32	8.2E-01	2.0E-04	1.00	0.88	1.00	1.00
1371239_s_at	tropomyosin 3, gamma	Tpm3	117557	1.11	7.31	7.4E-01	8.6E-04	1.00	1.00	1.00	1.00
1390687_at	pleckstrin	Plek	364206	1.66	7.30	6.2E-01	5.4E-04	0.00	0.00	1.00	0.00
1376327_at	tumor necrosis factor receptor superfamily, member 14 (herpesvirus entry mediator)	Tnfrsf14	366518	1.65	7.30	6.0E-01	5.1E-03	0.00	0.00	1.00	0.00
1398246_s_at	Fc receptor, IgG, low affinity III	Fcgr3	116591	1.10	7.30	6.8E-01	1.4E-03	1.00	1.00	1.00	1.00
1377064_at	dual specificity phosphatase 6	Dusp6	116663	0.98	7.29	9.7E-01	2.9E-04	1.00	0.50	1.00	1.00
1376255_at	mitogen activated protein kinase kinase kinase kinase 1 (predicted)	Map4k1_predicted	292763	1.07	7.27	9.6E-01	5.0E-03	0.00	0.00	1.00	0.00

				Fold Change		False Discovery Rate		Average Present Call			
				Strain Effect	Rejection Effect						
**Probe Set ID**	**Gene Title**	**Gene Symbol**	**Entrez Gene ID**	**Native BN / DA**	**Allograft / Isograft**	**Native BN / DA**	**Allograft / Isograft**	**Native BN**	**Native DA**	**Allograft**	**Isograft**
1387568_at	paired-Ig-like receptor B	Pirb	65146	1.05	7.21	9.7E-01	7.1E-04	0.00	0.00	1.00	0.25
1367786_at	proteosome (prosome, macropain) subunit, beta type 8	Psmb8	24968	1.34	7.18	3.5E-01	6.9E-04	1.00	1.00	1.00	1.00
1374976_a_at	Sterol O-acyltransferase 1	Soat1	81782	1.27	7.14	5.9E-01	1.0E-03	0.30	0.38	1.00	1.00
1369427_at	macrophage expressed gene 1	Mpeg1	64552	1.45	7.13	8.4E-01	1.2E-02	0.00	0.00	1.00	0.00
1378140_at	ADP-ribosylation factor-like 11	Arl11	364396	1.04	7.10	9.4E-01	4.3E-04	1.00	1.00	1.00	1.00
1369499_at	thymidylate synthase	Tyms	29261	0.75	7.08	8.7E-01	3.0E-03	0.00	0.00	0.80	0.00
1389696_at	Transcribed locus			2.11	7.05	5.0E-01	6.4E-04	0.00	0.00	1.00	0.25
1371033_at	RT1 class II, locus Bb	RT1-Bb	309622	0.92	7.02	8.4E-01	2.2E-04	1.00	1.00	1.00	1.00
1391505_x_at	Transcribed locus			0.76	6.93	6.7E-01	4.9E-04	0.20	0.75	1.00	1.00
1370463_x_at	RT1 class I, CE16	RT1-CE16	414819	2.61	6.90	2.2E-01	2.4E-03	1.00	0.00	1.00	0.75
1388054_a_at	chondroitin sulfate proteoglycan 2	Cspg2	114122	1.86	6.81	5.9E-01	2.7E-03	0.00	0.25	1.00	0.75
1388940_at	similar to Rho-GTPase-activating protein 25 (predicted)	RGD1562105_predicted	500246	0.28	6.81	1.3E-01	3.8E-03	0.00	0.00	1.00	0.00
1376652_at	complement component 1, q subcomponent, alpha polypeptide	C1qa	298566	1.19	6.80	5.7E-01	1.5E-04	1.00	1.00	1.00	1.00
1370215_at	complement component 1, q subcomponent, beta polypeptide	C1qb	29687	1.40	6.78	4.4E-01	7.5E-04	1.00	1.00	1.00	1.00
1374794_at	kinesin family member 15	Kif15	353302	0.65	6.77	6.4E-01	1.0E-02	0.00	0.00	1.00	0.00
1372685_at	cyclin-dependent kinase inhibitor 3 (predicted)	Cdkn3_predicted	289993	0.31	6.76	3.9E-01	9.6E-04	0.00	0.00	1.00	0.25
1373544_at	chemokine (C-X-C motif) ligand 9	Cxcl9	246759	1.27	6.76	5.0E-01	1.1E-03	1.00	1.00	1.00	1.00
1368494_at	S100 calcium binding protein A8 (calgranulin A)	S100a8	116547	1.11	6.71	9.4E-01	9.5E-03	0.70	0.50	1.00	0.50
1379497_at	Transcribed locus			0.85	6.69	7.6E-01	4.5E-04	0.10	0.25	1.00	0.88
1367942_at	acid phosphatase 5, tartrate resistant	Acp5	25732	1.16	6.67	8.3E-01	7.9E-04	0.00	0.00	1.00	0.63
1369953_a_at	CD24 antigen	Cd24	25145	1.89	6.67	4.9E-01	9.1E-03	0.30	0.00	0.80	0.00
1389408_at	ribonucleotide reductase M2 (mapped)	Rrm2	362720	0.89	6.65	9.2E-01	4.7E-04	0.30	0.00	1.00	0.50
1387831_at	Inhibitor of Bruton agammaglobulinemia tyrosine kinase (predicted)	Ibtk_predicted	315858	0.64	6.58	5.2E-01	1.9E-02	0.00	0.00	0.60	0.00
1367850_at	Fc receptor, IgG, low affinity III	Fcgr3	116591	1.11	6.55	7.8E-01	5.9E-04	1.00	1.00	1.00	1.00
1387687_at	immunoglobulin superfamily, member 6	Igsf6	171064	1.12	6.53	8.4E-01	4.0E-04	0.40	0.00	1.00	0.75
1389409_at	similar to Testis derived transcript	LOC500040	500040	1.64	6.50	7.9E-01	3.0E-04	0.00	0.00	1.00	0.00
1387627_at	cd86 antigen	Cd86	56822	0.76	6.42	8.2E-01	1.7E-03	0.00	0.00	0.80	0.00
1369190_at	CD2 antigen	Cd2	497761	1.32	6.42	5.8E-01	1.9E-03	0.00	0.00	0.70	0.00
1376130_a_at	dystrobrevin, beta	Dtnb	362715	1.34	6.38	8.1E-01	2.2E-04	0.00	0.00	1.00	0.00
1387005_at	cathepsin S	Ctss	50654	0.88	6.37	5.5E-01	1.9E-04	1.00	1.00	1.00	1.00
1370394_at	gamma-2a immunoglobulin heavy chain	IgG-2a	367586	0.78	6.36	7.7E-01	9.0E-03	0.00	0.00	0.60	0.00
1376304_at	Transcribed locus			1.09	6.36	9.7E-01	2.0E-02	0.00	0.00	0.70	0.00
1388484_at	ubiquitin-conjugating enzyme E2C (predicted)	Ube2c_predicted	296368	0.79	6.31	6.0E-01	3.7E-04	0.00	0.25	1.00	0.50
1370281_at	fatty acid binding protein 5, epidermal	Fabp5	140868	0.95	6.31	8.6E-01	1.6E-04	1.00	1.00	1.00	1.00
1389553_at	dendritic cell inhibitory receptor 3	Dcir3	362431	0.99	6.30	9.9E-01	1.5E-03	0.50	0.50	1.00	1.00
1374902_at	IQ motif containing GTPase activating protein 3 (predicted)	Iqgap3_predicted	310621	0.88	6.28	8.6E-01	3.3E-03	0.00	0.00	1.00	0.00
1370265_at	arrestin, beta 2	Arrb2	25388	1.23	6.19	8.6E-01	8.1E-04	0.00	0.00	1.00	0.25
1390287_at	bridging integrator 2	Bin2	366988	0.89	6.14	9.3E-01	6.4E-04	0.00	0.00	1.00	0.63
1386859_at	transketolase	Tkt	64524	1.05	6.13	9.3E-01	1.4E-04	1.00	1.00	1.00	1.00
1387378_at	ficolin B	Fcnb	114091	2.26	6.11	2.2E-01	2.9E-03	0.00	0.00	1.00	0.00
1390798_at	protein tyrosine phosphatase, receptor type, C	Ptprc	24699	1.50	6.09	1.2E-01	4.2E-04	0.80	0.75	1.00	1.00
1377351_at	sushi domain containing 3 (predicted)	Susd3_predicted	306810	0.73	6.04	8.5E-01	1.6E-03	0.00	0.00	1.00	0.00
1371960_at	YTH domain family 2 (predicted)	Ythdf2_predicted	313053	7.13	6.04	1.2E-01	1.1E-03	1.00	0.25	1.00	1.00
1387269_s_at	plasminogen activator, urokinase receptor	Plaur	50692	1.10	6.04	9.3E-01	3.7E-04	0.00	0.00	1.00	0.00
1387992_at	leukocyte immunoglobulin-like receptor, subfamily B (with TM and ITIM domains), member 3	Lilrb3	361493	2.28	6.02	5.2E-02	1.8E-03	0.40	0.13	0.80	0.00
1377239_at	amyloid beta (A4) precursor protein-binding, family B, member 1 interacting protein	Apbb1ip	307171	2.35	6.01	4.8E-01	3.7E-04	0.30	0.00	1.00	0.63
1370928_at	LPS-induced TN factor	Litaf	65161	1.58	5.99	1.1E-01	1.6E-04	1.00	1.00	1.00	1.00
1368247_at	heat shock 70kD protein 1A	Hspa1a	24472	1.07	5.98	7.8E-01	3.7E-04	0.80	0.88	1.00	1.00
1374961_at	similar to polyamine-modulated factor 1	LOC681050	681050	0.77	5.97	5.8E-01	2.5E-03	0.00	0.00	0.50	0.00
1387608_at	indoleamine-pyrrole 2,3 dioxygenase	Indo	66029	0.74	5.95	7.0E-01	3.8E-02	0.00	0.00	0.80	0.00
1370848_at	solute carrier family 2 (facilitated glucose transporter), member 1	Slc2a1	24778	0.65	5.93	1.5E-01	5.2E-04	0.60	1.00	1.00	1.00
1388337_at	nucleoside phosphorylase (mapped)	Np	290029	1.14	5.93	5.8E-01	6.1E-04	1.00	1.00	1.00	1.00
1387984_at	chemokine-like factor	Cklf	245978	1.63	5.92	7.4E-01	1.6E-02	0.00	0.00	1.00	0.00
1369688_s_at	protein tyrosine kinase 2 beta	Ptk2b	50646	1.37	5.91	7.4E-01	1.6E-03	0.00	0.00	1.00	0.00
1371016_at	similar to RIKEN cDNA A430107P09 gene	LOC290071	290071	0.98	5.89	9.8E-01	1.3E-03	1.00	1.00	1.00	1.00
1389092_at	interleukin 2 receptor, gamma (severe combined immunodeficiency)	Il2rg	140924	1.17	5.87	5.5E-01	1.4E-04	1.00	1.00	1.00	1.00
1373528_at				1.05	5.87	9.5E-01	4.8E-03	0.00	0.00	0.70	0.00
1371074_a_at	minichromosome maintenance deficient 6 (MIS5 homolog, S. pombe) (S. cerevisiae)	Mcm6	29685	1.84	5.85	6.7E-01	1.1E-03	0.00	0.00	0.80	0.00
1373611_at	interleukin 17 receptor (predicted)	Il17r_predicted	312679	1.82	5.83	2.2E-01	5.2E-05	1.00	0.75	1.00	1.00
1389911_at	meteorin, glial cell differentiation regulator-like	Metrnl	316842	0.89	5.83	4.1E-01	7.2E-04	1.00	1.00	1.00	1.00
1390137_at	TRAF4 associated factor 1	Traf4af1	311325	0.92	5.82	9.5E-01	4.2E-03	0.00	0.00	0.50	0.00
1371774_at	spermidine/spermine N1-acetyl transferase (mapped)	Sat	302642	1.32	5.82	2.9E-01	1.7E-04	1.00	1.00	1.00	1.00
1392785_at	Potassium channel tetramerisation domain containing 12 (predicted)	Kctd12_predicted	364458	3.12	5.80	1.6E-01	3.4E-03	0.50	0.00	1.00	0.75
1369186_at	caspase 1	Casp1	25166	1.14	5.80	7.7E-01	6.9E-04	1.00	0.75	1.00	1.00
1376709_at	solute carrier family 39 (metal ion transporter), member 8	Slc39a8	295455	1.69	5.76	1.2E-01	1.6E-04	1.00	0.50	1.00	1.00
1389248_at	galactokinase 1	Galk1	287835	0.82	5.73	5.7E-01	2.4E-03	0.00	0.00	0.80	0.00
1373490_at	Similar to chromosome 14 open reading frame 21 (predicted)	RGD1308396_predicted	290235	1.18	5.72	4.9E-01	3.0E-04	0.90	0.88	1.00	1.00
1389566_at	cyclin B2	Ccnb2	363088	0.93	5.71	9.3E-01	1.2E-03	0.00	0.00	1.00	0.00
1376089_at	Low density lipoprotein receptor-related protein 5 (predicted)	Lrp5_predicted	293649	1.70	5.70	1.2E-01	1.2E-03	0.50	0.00	1.00	1.00
1369204_at	hemopoietic cell kinase	Hck	25734	1.21	5.69	2.8E-01	6.4E-04	1.00	1.00	1.00	1.00
1374805_at	similar to hypothetical protein MGC5528 (predicted)	RGD1561749_predicted	299933	0.76	5.67	7.3E-01	1.2E-02	0.20	0.25	0.80	0.00
1376073_at	Sel1 (suppressor of lin-12) 1 homolog (C. elegans)	Sel1h	314352	2.25	5.66	4.2E-01	6.5E-03	0.00	0.00	1.00	0.00
1376026_at	downstream neighbor of SON	Donson	288257	0.67	5.65	4.6E-01	4.8E-02	0.00	0.00	1.00	0.00
1384787_at	CD3 antigen, gamma polypeptide	Cd3g	300678	1.39	5.65	7.6E-01	2.5E-03	0.00	0.00	1.00	0.00
1369724_at	coagulation factor XIII, A1 subunit	F13a1	60327	1.33	5.64	7.0E-01	1.1E-03	0.70	0.25	1.00	0.63
1389030_a_at	Rous sarcoma oncogene	Src	83805	1.42	5.62	8.6E-01	1.7E-02	0.00	0.00	0.70	0.00
1370603_a_at	protein tyrosine phosphatase, receptor type, C	Ptprc	24699	0.86	5.62	9.0E-01	2.0E-02	0.00	0.00	1.00	0.00

				Fold Change		False Discovery Rate		Average Present Call			
				Strain Effect	Rejection Effect						
**Probe Set ID**	**Gene Title**	**Gene Symbol**	**Entrez Gene ID**	**Native BN / DA**	**Allograft / Isograft**	**Native BN / DA**	**Allograft / Isograft**	**Native BN**	**Native DA**	**Allograft**	**Isograft**
1377058_at	similar to solute carrier family 37 (glycerol-3-phosphate transporter), member 2 (predicted)	RGD1564160_predicted	500973	0.36	5.60	5.0E-01	2.8E-03	0.00	0.00	1.00	0.00
1376076_at	Membrane protein, palmitoylated 5 (MAGUK p55 subfamily member 5) (predicted)	Mpp5_predicted	314259	0.96	5.56	9.1E-01	2.8E-04	1.00	0.75	1.00	1.00
1387759_s_at	UDP glycosyltransferase 1 family, polypeptide A1	Ugt1a1	113992	1.61	5.56	1.4E-01	7.9E-04	0.60	0.00	1.00	0.75
1398303_s_at	tropomyosin 3, gamma	Tpm3	117557	1.55	5.54	1.6E-01	3.1E-03	0.70	0.00	1.00	1.00
1370469_at	protein tyrosine phosphatase, non-receptor type 7	Ptpn7	246781	0.92	5.53	9.6E-01	9.3E-03	0.00	0.00	0.90	0.00
1373746_at	similar to HSPC049 protein (predicted)	RGD1306697_predicted	312225	0.53	5.53	6.8E-01	7.9E-04	0.20	0.00	1.00	0.00
1389695_at	similar to cDNA sequence BC004044	RGD1311980	304334	1.01	5.53	9.9E-01	1.1E-04	0.20	0.25	1.00	1.00
1377185_at	similar to RIKEN cDNA 6330442E10 gene (predicted)	RGD1562622_predicted	503035	0.86	5.52	7.9E-01	9.7E-03	0.00	0.00	0.70	0.00
1370024_at	Similar to RIKEN cDNA 4931400A14 (predicted)	RGD1307234_predicted	316732	0.96	5.52	9.9E-01	4.8E-02	0.00	0.00	1.00	0.00
1376231_at	similar to Hypothetical UPF0080 protein KIAA0186 (predicted)	RGD1562246_predicted	499914	1.43	5.51	7.9E-01	1.4E-02	0.00	0.00	0.50	0.00
1377023_at	dual specificity phosphatase 2	Dusp2	311406	1.15	5.49	7.7E-01	2.5E-04	0.50	0.63	1.00	0.38
1387742_at	chemokine (C-C motif) receptor 2	Ccr2	60463	1.13	5.49	9.1E-01	8.2E-03	0.00	0.00	0.60	0.00
1388272_at	immunoglobulin heavy chain 1a (serum IgG2a)	Igh-1a	299352	1.09	5.47	9.6E-01	1.1E-02	0.00	0.00	0.80	0.00
1369815_at	chemokine (C-C motif) ligand 3	Ccl3	25542	0.84	5.47	8.5E-01	3.7E-04	0.00	0.00	1.00	0.38
1375428_at	cellular repressor of E1A-stimulated genes (predicted)	Creg_predicted	289185	1.61	5.45	3.8E-01	4.3E-04	1.00	0.75	1.00	0.38
1367955_at	RAB4B, member RAS oncogene family	Rab4b	50866	1.61	5.44	4.8E-01	1.5E-02	0.00	0.00	0.60	0.00
1370234_at	fibronectin 1	Fn1	25661	1.65	5.41	2.5E-01	1.5E-03	1.00	1.00	1.00	1.00
1387180_at	interleukin 1 receptor, type II	Il1r2	117022	1.63	5.39	6.3E-01	5.6E-02	0.20	0.00	1.00	0.00
1386879_at	lectin, galactose binding, soluble 3	Lgals3	83781	1.24	5.39	6.6E-01	4.1E-04	1.00	1.00	1.00	1.00
1370942_at	RAS p21 protein activator 3	Rasa3	29372	2.06	5.37	5.4E-01	1.0E-02	0.00	0.00	1.00	0.00
1370883_at	RT1 class II, locus Da	RT1-Da	294269	0.89	5.34	6.9E-01	4.3E-04	1.00	1.00	1.00	1.00
1368527_at	prostaglandin-endoperoxide synthase 2	Ptgs2	29527	0.73	5.34	8.5E-01	1.2E-02	0.10	0.13	1.00	0.75
1369181_at	cytochrome b-245, beta polypeptide	Cybb	66021	1.01	5.33	9.9E-01	7.1E-04	0.20	0.25	1.00	0.50
1383426_at	proline-serine-threonine phosphatase-interacting protein 1 (predicted)	Pstpip1_predicted	300732	1.11	5.33	7.6E-01	4.4E-05	0.60	0.75	1.00	0.88
1369683_at	BH3 interacting domain death agonist	Bid	64625	2.09	5.33	4.5E-01	3.3E-02	0.00	0.00	0.80	0.00
1388265_x_at	chondroitin sulfate proteoglycan 2	Cspg2	114122	0.64	5.30	6.1E-01	1.5E-02	0.00	0.00	1.00	0.13
1387101_at	acyl-CoA synthetase long-chain family member 4	Acsl4	113976	1.16	5.27	3.6E-01	2.4E-04	1.00	1.00	1.00	1.00
1368742_at	complement component 5, receptor 1	C5r1	113959	1.15	5.27	6.7E-01	3.2E-04	1.00	1.00	1.00	1.00
1367856_at	glucose-6-phosphate dehydrogenase X-linked	G6pdx	24377	2.20	5.26	3.3E-01	4.4E-05	1.00	0.25	1.00	1.00
1370967_at	Claudin 10 (predicted)	Cldn10_predicted	290485	2.07	5.25	5.5E-01	1.3E-02	0.00	0.00	1.00	0.00
1368260_at	aurora kinase B	Aurkb	114592	1.14	5.24	8.8E-01	6.3E-04	0.60	0.00	1.00	0.50
1368407_at	heparanase	Hpse	64537	3.26	5.23	4.1E-01	2.0E-02	0.00	0.00	1.00	0.00
1370832_at	Alpha thalassemia/mental retardation syndrome X-linked homolog (human)	Atrx	246284	0.84	5.22	4.7E-01	9.9E-04	1.00	1.00	1.00	1.00
1389123_at	chemokine (C-C motif) ligand 6	Ccl6	287910	1.73	5.22	2.7E-01	8.9E-04	1.00	1.00	1.00	1.00
1387969_at	chemokine (C-X-C motif) ligand 10	Cxcl10	245920	1.54	5.21	4.7E-02	9.4E-03	0.80	0.13	1.00	0.75
1370585_a_at	protein kinase C, beta 1	Prkcb1	25023	1.32	5.20	5.4E-01	4.6E-04	1.00	1.00	1.00	1.00
1379766_at	Src-like adaptor	Sla	338477	1.59	5.14	6.7E-01	3.0E-04	0.00	0.00	1.00	0.63
1390659_at	Transcribed locus			1.43	5.13	3.8E-01	1.5E-04	1.00	1.00	1.00	1.00
1390389_at	C-mer proto-oncogene tyrosine kinase	Mertk	65037	0.92	5.10	9.1E-01	9.7E-04	0.00	0.00	1.00	0.00
1390674_at	Transcribed locus			0.56	5.08	3.7E-01	1.3E-02	0.00	0.00	0.60	0.00
1372200_at	G-protein signalling modulator 3 (AGS3-like, C. elegans)	Gpsm3	406163	1.05	5.08	9.8E-01	1.3E-03	0.00	0.00	1.00	0.25
1389413_at	similar to ecotropic viral integration site 2A	LOC685433	685433	0.67	5.06	5.0E-01	2.0E-04	0.00	0.00	1.00	0.50
1389006_at	Transcribed locus			1.38	5.03	4.9E-01	7.9E-04	0.70	0.00	1.00	1.00
1368921_a_at	CD44 antigen	Cd44	25406	1.33	5.02	6.4E-01	1.2E-04	0.80	0.38	1.00	1.00
1388142_at	chondroitin sulfate proteoglycan 2	Cspg2	114122	1.13	5.01	8.6E-01	6.7E-03	0.40	0.25	1.00	0.75
1369672_at	arachidonate 5-lipoxygenase activating protein	Alox5ap	29624	1.18	4.99	7.6E-01	1.1E-03	1.00	0.38	1.00	1.00
1368027_at	thromboxane A synthase 1	Tbxas1	24886	0.99	4.99	9.8E-01	1.3E-04	0.00	0.13	1.00	0.88
1370186_at	proteosome (prosome, macropain) subunit, beta type 9	Psmb9	24967	0.92	4.97	7.7E-01	1.3E-03	1.00	1.00	1.00	1.00
1377867_at	similar to Glutaminyl-peptide cyclotransferase precursor (QC) (predicted)	RGD1562284_predicted	313837	0.64	4.97	2.9E-01	7.8E-04	0.20	0.88	1.00	0.88
1368000_at	complement component 3	C3	24232	1.53	4.95	3.6E-01	2.9E-03	1.00	0.88	1.00	1.00
1368412_a_at	protein tyrosine phosphatase, receptor type, O	Ptpro	50677	0.77	4.93	4.6E-01	9.9E-04	0.00	0.00	1.00	1.00
1368179_at	thymic stromal-derived lymphopoietin, receptor	Tslpr	171499	1.64	4.92	6.2E-01	5.7E-03	0.00	0.00	1.00	0.00
1376675_at	similar to CD300A antigen	LOC501736	501736	0.97	4.92	9.6E-01	9.8E-04	0.00	0.00	1.00	0.75
1370097_a_at	chemokine (C-X-C motif) receptor 4	Cxcr4	60628	1.43	4.92	3.0E-01	6.5E-04	1.00	0.75	1.00	1.00
1368799_at	baculoviral IAP repeat-containing 5	Birc5	64041	1.28	4.91	8.4E-01	1.5E-02	0.00	0.00	0.60	0.00
1368046_at	solute carrier family 31 (copper transporters), member 1	Slc31a1	171135	2.87	4.90	2.9E-01	3.3E-02	0.00	0.00	0.60	0.00
1389440_at	Transcribed locus			1.03	4.88	9.9E-01	4.3E-03	0.00	0.00	0.90	0.00
1369031_at	interleukin 18 binding protein	Il18bp	84388	0.83	4.87	4.3E-01	1.3E-03	0.90	1.00	1.00	1.00
1388650_at	topoisomerase (DNA) 2 alpha	Top2a	360243	1.01	4.87	9.9E-01	2.4E-03	0.00	0.00	1.00	0.50
1371961_at	phospholipase D family, member 3	Pld3	361527	1.04	4.87	9.4E-01	1.7E-03	0.80	0.63	1.00	1.00
1387952_a_at	CD44 antigen	Cd44	25406	2.43	4.85	1.1E-01	2.4E-03	0.00	0.00	1.00	0.50
1373207_at	Transcribed locus			0.63	4.83	4.5E-01	7.2E-04	0.00	0.25	1.00	1.00
1387956_s_at	chemokine-like factor	Cklf	245978	0.90	4.81	9.5E-01	1.6E-02	0.00	0.00	0.80	0.00
1376914_at	DEP domain containing 1a (predicted)	Depdc1a_predicted	295538	0.87	4.81	9.2E-01	7.5E-02	0.00	0.00	0.70	0.00
1379387_at	Regulator of G-protein signaling 18	Rgs18	289076	1.07	4.77	9.6E-01	7.2E-03	0.00	0.00	1.00	0.50
1376784_at	Formin binding protein 1	Fnbp1	192348	1.89	4.76	5.9E-01	1.3E-02	0.00	0.00	0.90	0.00
1372064_at	similar to chemokine (C-X-C motif) ligand 16	Cxcl16	497942	0.94	4.75	8.7E-01	3.3E-04	1.00	1.00	1.00	1.00
1393688_at	Complement component 6	C6	24237	1.22	4.75	9.0E-01	4.2E-04	0.00	0.00	1.00	0.25
1370339_at				0.89	4.75	4.2E-01	2.0E-04	1.00	1.00	1.00	1.00
1370740_at	killer cell lectin-like receptor, subfamily A, member 5	Klra5	297666	1.10	4.75	9.7E-01	1.4E-02	0.00	0.00	1.00	0.13
1376943_at				1.09	4.73	8.6E-01	4.8E-04	0.90	0.50	1.00	0.75
1387808_at	solute carrier family 7 (cationic amino acid transporter, y+ system), member 7	Slc7a7	83509	1.20	4.72	5.8E-01	3.7E-04	1.00	1.00	1.00	1.00
1388460_at	capping protein (actin filament), gelsolin-like	Capg	297339	0.98	4.72	9.5E-01	2.8E-04	1.00	1.00	1.00	1.00
1373504_at	GLI pathogenesis-related 1 (glioma)	Glipr1	299783	1.06	4.71	7.9E-01	5.1E-04	1.00	1.00	1.00	1.00
1374544_at	hypothetical protein LOC679150	LOC679150	679150	1.29	4.71	4.8E-01	1.2E-04	0.00	0.00	1.00	0.63
1387048_at	DEAD (Asp-Glu-Ala-Asp) box polypeptide 39	Ddx39	89827	1.03	4.70	9.4E-01	6.4E-04	0.80	0.38	1.00	1.00
1388149_at	transporter 1, ATP-binding cassette, sub-family B (MDR/TAP)	Tap1	24811	1.66	4.70	6.7E-01	4.4E-04	0.00	0.00	1.00	0.00

				Fold Change		False Discovery Rate		Average Present Call			
				Strain Effect	Rejection Effect						
Probe Set ID	Gene Title	Gene Symbol	Entrez Gene ID	Native BN / DA	Allograft / Isograft	Native BN / DA	Allograft / Isograft	Native BN	Native DA	Allograft	Isograft
1368207_at	FXYD domain-containing ion transport regulator 5	Fxyd5	60338	1.27	4.69	5.7E-01	4.1E-04	1.00	0.75	1.00	1.00
1387834_at	megakaryocyte-associated tyrosine kinase	Matk	60450	1.39	4.69	8.6E-01	1.4E-02	0.00	0.00	1.00	0.00
1373575_at	similar to NADH dehydrogenase (ubiquinone) Fe-S protein 2	LOC498279	498279	1.31	4.69	1.5E-01	5.5E-04	1.00	1.00	1.00	1.00
1368808_at	CAP, adenylate cyclase-associated protein 1 (yeast)	Cap1	64185	1.67	4.69	1.3E-01	1.3E-03	1.00	0.50	1.00	1.00
1368975_at	CD38 antigen	Cd38	25668	0.90	4.68	8.8E-01	8.4E-04	0.00	0.50	1.00	0.75
1368826_at	catechol-O-methyltransferase	Comt	24267	0.95	4.68	7.9E-01	3.9E-04	1.00	1.00	1.00	1.00
1368464_at	macrophage galactose N-acetyl-galactosamine specific lectin 1	Mgl1	64195	0.92	4.67	8.8E-01	1.4E-03	0.00	0.00	1.00	1.00
1375211_at	ribonuclease T2 (predicted)	Rnaset2_predicted	292306	1.02	4.67	9.6E-01	2.8E-04	1.00	1.00	1.00	1.00
1373823_at	similar to Cyclin-dependent kinases regulatory subunit 2 (CKS-2) (predicted)	RGD1562047_predicted	498709	1.45	4.67	8.2E-01	3.0E-03	0.00	0.00	1.00	0.38
1371803_at	GM2 ganglioside activator protein	Gm2a	282838	1.07	4.65	9.2E-01	5.3E-04	0.90	0.63	1.00	1.00
1390850_at	Adipose differentiation related protein	Adfp	298199	2.43	4.65	4.9E-01	4.4E-04	0.00	0.00	1.00	0.00
1372082_at	NECAP endocytosis associated 2	Necap2	298598	1.03	4.65	9.7E-01	2.5E-04	1.00	1.00	1.00	1.00
1388848_at	similar to hypothetical protein MGC13251	RGD1308350	362790	0.80	4.64	5.5E-01	3.7E-03	0.00	0.00	1.00	0.00
1369173_at	complement component 3a receptor 1	C3ar1	84007	1.78	4.63	6.2E-01	6.5E-04	1.00	0.25	1.00	0.63
1372852_at	similar to protein tyrosine phosphatase, receptor type, C polypeptide-associated protein	LOC499300	499300	0.69	4.63	7.4E-01	1.5E-03	0.00	0.00	1.00	0.25
1371184_x_at	tropomyosin 3, gamma	Tpm3	117557	0.79	4.62	7.3E-01	5.6E-03	1.00	0.75	1.00	1.00
1370393_at	coiled-coil domain containing 5	Ccdc5	192228	1.88	4.60	6.6E-01	1.2E-02	0.00	0.00	0.70	0.00
1377153_a_at	kelch-like 6 (Drosophila) (predicted)	Klhl6_predicted	287974	1.38	4.60	4.3E-01	4.6E-04	1.00	0.75	1.00	1.00
1371782_at	nipsnap homolog 3A (C. elegans)	Nipsnap3a	313211	0.75	4.60	7.6E-01	2.7E-04	0.00	0.00	1.00	0.75
1389997_at	CD3 antigen, epsilon polypeptide (predicted)	Cd3e_predicted	315609	0.76	4.60	7.7E-01	1.3E-03	0.00	0.00	1.00	0.00
1387202_at	intercellular adhesion molecule 1	Icam1	25464	1.82	4.58	4.3E-01	5.7E-03	0.00	0.00	1.00	0.25
1368455_at	natural killer cell group 7 sequence	Nkg7	171062	1.20	4.58	6.8E-01	3.6E-03	0.80	0.75	1.00	1.00
1371646_at	phosphogluconate dehydrogenase (mapped)	Pgd	362660	1.11	4.57	8.0E-01	3.0E-04	1.00	1.00	1.00	1.00
1388275_at	T-cell receptor beta chain	Tcrb	24820	1.12	4.57	9.5E-01	1.7E-03	0.00	0.13	1.00	0.50
1369814_at	chemokine (C-C motif) ligand 20	Ccl20	29538	1.07	4.55	9.5E-01	1.1E-02	0.20	0.00	1.00	0.00
1376006_at	Transcribed locus			1.06	4.53	8.5E-01	2.2E-04	0.60	0.13	1.00	1.00
1377340_at	tissue factor pathway inhibitor 2	Tfpi2	286926	1.29	4.52	6.2E-01	1.7E-03	1.00	0.75	1.00	1.00
1383684_at	ASF1 anti-silencing function 1 homolog B (S. cerevisiae) (predicted)	Asf1b_predicted	304648	1.10	4.51	9.2E-01	1.2E-03	0.20	0.25	1.00	0.50
1372516_at	kinesin family member 22	Kif22	293502	1.36	4.51	5.4E-01	3.6E-04	0.00	0.00	1.00	0.00
1387667_at	nitric oxide synthase 2, inducible	Nos2	24599	1.15	4.50	8.9E-01	3.6E-03	0.00	0.13	1.00	0.00
1377832_at	polo-like kinase 4 (Drosophila) (predicted)	Plk4_predicted	310344	0.52	4.50	4.3E-01	3.6E-03	0.20	1.00	1.00	0.25
1390325_at	CD38 antigen	Cd38	25668	0.88	4.49	4.3E-01	1.4E-04	1.00	1.00	1.00	1.00
1387483_at	phospholipase C, gamma 2	Plcg2	29337	1.09	4.48	9.6E-01	1.7E-02	0.00	0.00	0.70	0.00
1368679_a_at	Yamaguchi sarcoma viral (v-yes-1) oncogene homolog	Lyn	81515	1.13	4.48	8.1E-01	8.5E-04	1.00	1.00	1.00	1.00
1368187_at	glycoprotein (transmembrane) nmb	Gpnmb	113955	1.77	4.46	2.2E-01	7.4E-03	1.00	0.00	1.00	1.00
1370912_at	heat shock 70kD protein 1B (mapped)	Hspa1b	294254	1.45	4.46	1.1E-01	2.8E-04	1.00	1.00	1.00	1.00
1368258_at	apelin, AGTRL1 ligand	Apln	58812	0.78	4.45	5.4E-01	3.7E-04	1.00	1.00	1.00	0.63
1386925_at	actin related protein 2/3 complex, subunit 1B	Arpc1b	54227	1.43	4.45	8.1E-02	1.3E-04	1.00	1.00	1.00	1.00
1368379_at	scavenger receptor class B, member 2	Scarb2	117106	0.72	4.45	3.5E-01	5.1E-04	0.40	0.63	1.00	1.00
1387154_at	neuropeptide Y	Npy	24604	0.67	4.43	1.9E-01	1.8E-03	1.00	1.00	1.00	1.00
1372585_at	RGD1566254 (predicted)	RGD1566254_predicted	499318	1.17	4.42	6.3E-01	1.0E-03	1.00	1.00	1.00	1.00
1367905_at	ectonucleotide pyrophosphatase/phosphodiesterase 3	Enpp3	54410	1.83	4.39	8.7E-02	3.8E-04	1.00	0.75	1.00	1.00
1367632_at	glutamate-ammonia ligase (glutamine synthase)	Glul	24957	1.49	4.36	2.7E-01	1.3E-04	1.00	1.00	1.00	1.00
1368010_at	protein tyrosine phosphatase, non-receptor type 6	Ptpn6	116689	0.98	4.36	9.3E-01	4.8E-04	0.00	0.25	1.00	1.00
1367676_at	high mobility group box 2	Hmgb2	29395	1.47	4.35	1.9E-01	6.3E-04	1.00	1.00	1.00	1.00
1387076_at	hypoxia inducible factor 1, alpha subunit	Hif1a	29560	1.02	4.35	9.6E-01	4.4E-05	1.00	1.00	1.00	1.00
1371946_at	kidney predominant protein NCU-G1	RGD1303130	295231	1.10	4.33	8.0E-01	2.7E-04	1.00	1.00	1.00	1.00
1390226_at	similar to hypothetical protein LOC340061 (predicted)	RGD1562552_predicted	498840	1.81	4.33	6.4E-01	5.4E-04	0.00	0.00	1.00	0.00
1369979_at	src family associated phosphoprotein 2	Scap2	155183	1.26	4.32	6.0E-01	2.7E-03	0.00	0.00	1.00	0.50
1367776_at	cell division cycle 2 homolog A (S. pombe)	Cdc2a	54237	0.89	4.32	9.0E-01	2.8E-03	0.00	0.00	1.00	0.38
1370531_a_at	phospholipase D1	Pld1	25096	1.03	4.31	9.9E-01	1.7E-02	0.00	0.00	1.00	0.00
1374778_at	Similar to GA binding protein transcription factor, beta subunit 2 (GABPB2) (predicted)	RGD1560391_predicted	499883	1.28	4.29	5.4E-01	2.2E-04	1.00	1.00	1.00	1.00
1367633_at	glutamate-ammonia ligase (glutamine synthase)	Glul	24957	1.42	4.26	1.3E-01	1.5E-03	1.00	1.00	1.00	1.00
1368420_at	ceruloplasmin	Cp	24268	1.24	4.26	7.2E-01	7.6E-04	0.90	0.50	1.00	1.00
1387918_at	tropomyosin 3, gamma	Tpm3	117557	1.74	4.26	5.7E-01	3.2E-03	0.00	0.00	0.80	0.00
1388784_at	colony stimulating factor 1 receptor	Csf1r	307403	1.34	4.25	4.6E-01	1.2E-04	1.00	1.00	1.00	1.00
1373661_a_at	chemokine (C-X-C motif) receptor 4	Cxcr4	60628	1.05	4.25	9.2E-01	6.2E-04	1.00	1.00	1.00	1.00
1372890_at	Transcribed locus			0.53	4.24	1.7E-01	4.9E-04	0.00	0.00	1.00	0.25
1370177_at	poliovirus receptor	PVR	25066	1.74	4.21	3.4E-01	1.1E-03	0.30	0.00	1.00	0.00
1372056_at	CKLF-like MARVEL transmembrane domain containing 6	Cmtm6	316035	1.11	4.19	7.7E-01	1.9E-04	1.00	1.00	1.00	1.00
1373850_at	sphingomyelin phosphodiesterase, acid-like 3B	Smpdl3b	362619	1.20	4.19	7.9E-01	1.5E-03	0.00	0.00	0.90	0.00
1372404_at	RAS-related C3 botulinum substrate 2	Rac2	366957	1.02	4.19	9.2E-01	3.5E-03	1.00	1.00	1.00	1.00
1372478_at	similar to chemokine-like factor super family 7	LOC501065	501065	1.10	4.18	4.9E-01	5.4E-04	1.00	0.50	1.00	1.00
1376151_a_at	Similar to novel protein similar to human oligophrenin 1 (OPHN1) (predicted)	RGD1564081_predicted	500943	1.18	4.17	5.5E-01	4.6E-03	1.00	1.00	1.00	1.00
1389250_at	Transcribed locus			1.11	4.14	9.4E-01	1.9E-03	0.00	0.00	1.00	0.25
1377385_at	Rho GTPase activating protein 27	Arhgap27	303583	3.29	4.14	2.5E-01	1.1E-02	0.00	0.00	1.00	0.38
1368073_at	interferon regulatory factor 1	Irf1	24508	1.40	4.14	3.5E-01	1.2E-03	1.00	1.00	1.00	1.00
1370219_at	cytochrome b-245, alpha polypeptide	Cyba	79129	0.99	4.13	9.8E-01	1.2E-03	1.00	1.00	1.00	1.00
1372303_at	similar to 0910001A06Rik protein (predicted)	RGD1307697_predicted	299909	0.99	4.13	9.9E-01	1.8E-03	1.00	0.88	1.00	1.00
1387057_at	solute carrier family 7 (cationic amino acid transporter, y+ system), member 8	Slc7a8	192117	1.46	4.12	6.5E-01	3.3E-03	0.00	0.00	0.80	0.00
1371529_at	receptor (TNFRSF)-interacting serine-threonine kinase 1 (predicted)	Ripk1_predicted	306886	1.56	4.12	7.7E-01	9.3E-04	0.00	0.00	1.00	0.25
1367677_at	peroxiredoxin 5	Prdx5	113898	0.96	4.11	9.5E-01	3.7E-04	1.00	1.00	1.00	1.00
1387354_at	signal transducer and activator of transcription 1	Stat1	25124	1.36	4.10	3.4E-01	2.2E-04	1.00	1.00	1.00	1.00
1383320_at	lymphocyte protein tyrosine kinase (mapped)	Lck	313050	0.99	4.08	9.8E-01	3.2E-04	1.00	1.00	1.00	1.00
1369387_at	vav 1 oncogene	Vav1	25156	0.87	4.07	7.8E-01	9.8E-04	0.00	0.00	1.00	0.50
1368605_at	adaptor protein with pleckstrin homology and src homology 2 domains	Aps	114203	0.86	4.07	8.2E-01	1.6E-03	0.00	0.25	1.00	0.50
1391946_at	selectin, platelet	Selp	25651	0.50	4.07	5.3E-01	2.8E-03	0.00	0.00	1.00	0.00

				Fold Change		False Discovery Rate		Average Present Call			
				Strain Effect	Rejection Effect						
Probe Set ID	Gene Title	Gene Symbol	Entrez Gene ID	Native BN / DA	Allograft / Isograft	Native BN / DA	Allograft / Isograft	Native BN	Native DA	Allograft	Isograft
1368270_at	apolipoprotein B editing complex 1	Apobec1	25383	0.44	4.06	4.8E-01	4.7E-03	0.00	0.00	1.00	0.50
1370340_x_at	tropomyosin 3, gamma	Tpm3	117557	1.36	4.04	3.7E-01	3.2E-03	0.00	0.00	1.00	0.00
1387982_at	toll-like receptor 4	Tlr4	29260	0.89	4.03	8.2E-01	5.6E-03	0.60	0.88	1.00	0.75
1374518_at	transmembrane protein 77	Tmem77	362011	1.20	4.03	2.5E-01	4.3E-04	1.00	1.00	1.00	1.00
1389732_at	Similar to CG4025-PA	LOC679937	679937	0.97	4.02	9.2E-01	6.5E-04	1.00	1.00	1.00	1.00
1374033_at	proteasome (prosome, macropain) subunit, beta type 10	Psmb10	291983	0.84	4.02	6.6E-01	3.6E-03	1.00	1.00	1.00	1.00
1368811_at	lamin B1	Lmnb1	116685	0.70	4.00	8.1E-01	4.2E-04	0.00	0.00	1.00	0.00
1371040_at	solute carrier family 1 (neutral amino acid transporter), member 5	Slc1a5	292657	0.72	4.00	8.3E-01	5.3E-04	0.00	0.00	1.00	0.00
1387739_at	CD8 antigen, beta chain	Cd8b	24931	0.49	3.98	6.0E-01	2.7E-03	0.00	0.00	1.00	0.00
1371194_at	tumor necrosis factor alpha induced protein 6	Tnfaip6	84397	1.73	3.97	5.4E-01	6.1E-02	0.00	0.00	1.00	0.50
1398523_a_at	interferon (alpha and beta) receptor 1 (predicted)	Ifnar1_predicted	288264	0.93	3.97	9.2E-01	1.2E-04	0.60	0.50	1.00	0.75
1371970_at	similar to expressed sequence AW413625 (predicted)	RGD1560913_predicted	499322	1.20	3.96	8.8E-01	3.0E-04	0.00	0.00	1.00	0.00
1387221_at	GTP cyclohydrolase 1	Gch	29244	1.03	3.94	9.5E-01	1.3E-02	0.00	0.25	1.00	0.00
1368809_at	CAP, adenylate cyclase-associated protein 1 (yeast)	Cap1	64185	0.86	3.94	5.9E-01	2.5E-04	1.00	1.00	1.00	1.00
1373250_at	similar to Anillin (predicted)	RGD1566097_predicted	307056	0.87	3.94	9.5E-01	1.4E-02	0.00	0.00	1.00	0.25
1371932_at	similar to hypothetical protein FLJ38482	RGD1309341	361137	0.82	3.93	4.6E-01	5.7E-04	0.00	0.00	1.00	0.00
1373494_at	breakpoint cluster region (predicted)	Bcr_predicted	309696	1.69	3.89	7.7E-01	8.8E-02	0.00	0.00	1.00	0.13
1373613_at	similar to RIKEN cDNA 4930570C03	LOC300191	300191	1.00	3.89	9.9E-01	2.0E-04	1.00	1.00	1.00	1.00
1387432_at	ROD1 regulator of differentiation 1 (S. pombe)	Rod1	83515	1.32	3.88	8.7E-01	5.5E-02	0.00	0.00	0.80	0.00
1377116_at	Cation channel, sperm associated 3 (predicted)	Catsper3_predicted	290989	1.01	3.86	9.7E-01	6.0E-04	1.00	1.00	1.00	1.00
1374388_at	EF hand domain containing 2	Efhd2	298609	1.28	3.86	4.1E-01	1.4E-04	1.00	1.00	1.00	1.00
1374393_at	protein tyrosine phosphatase-like (proline instead of catalytic arginine), member b (predicted)	Ptplb_predicted	288058	1.40	3.85	7.8E-01	1.4E-04	0.40	0.00	1.00	0.63
1390348_at	folate receptor 2 (fetal) (predicted)	Folr2_predicted	293154	1.07	3.85	8.0E-01	4.6E-04	0.00	0.00	1.00	1.00
1373818_at	Potassium channel tetramerisation domain containing 12 (predicted)	Kctd12_predicted	364458	1.22	3.84	5.4E-01	2.6E-04	1.00	1.00	1.00	1.00
1399148_s_at	glycosyltransferase 25 domain containing 1 (predicted)	Glt25d1_predicted	290637	1.35	3.84	8.4E-01	5.6E-02	0.00	0.00	1.00	0.13
1387074_at	regulator of G-protein signaling 2	Rgs2	84583	1.86	3.82	5.3E-02	3.5E-04	1.00	1.00	1.00	1.00
1371123_x_at	RT1 class Ib, locus S3	RT1-S3	294228	1.76	3.81	1.5E-01	2.5E-03	1.00	1.00	1.00	1.00
1373514_at	similar to chromosome 17 open reading frame 27	LOC688296	688296	0.93	3.80	9.3E-01	4.0E-03	0.00	0.00	1.00	0.00
1390510_at	membrane-spanning 4-domains, subfamily A, member 6B	Ms4a6b	293749	0.97	3.80	9.5E-01	7.7E-04	1.00	1.00	1.00	1.00
1388282_s_at	killer cell lectin-like receptor, subfamily A, member 5	Klra5	297666	2.73	3.80	3.6E-01	9.9E-03	0.00	0.00	1.00	0.13
1388667_at	mannosidase 2, alpha 1	Man2a1	25478	1.98	3.79	5.5E-01	1.7E-03	0.00	0.00	1.00	1.00
1373897_at	Ubiquitin carboxyl-terminal hydrolase L5	Uchl5	360853	1.03	3.79	9.6E-01	2.8E-04	1.00	0.88	1.00	1.00
1376795_at	Phosphoinositide-3-kinase adaptor protein 1 (predicted)	Pik3ap1_predicted	294048	1.66	3.79	2.9E-01	1.6E-04	1.00	0.00	1.00	1.00
1372070_at	interferon gamma inducible protein 30	Ifi30	290644	1.13	3.79	5.6E-01	9.2E-04	1.00	1.00	1.00	1.00
1388213_a_at	RT1 class Ib, locus S3	RT1-S3	294228	1.81	3.78	2.9E-01	5.3E-03	1.00	1.00	1.00	1.00
1389244_x_at	chemokine (C-X-C motif) receptor 4	Cxcr4	60628	1.18	3.77	5.7E-01	7.9E-04	1.00	1.00	1.00	1.00
1387947_at	v-maf musculoaponeurotic fibrosarcoma oncogene family, protein B (avian)	Mafb	54264	0.63	3.77	6.7E-01	5.8E-03	0.00	0.00	1.00	0.25
1374678_at	sema domain, immunoglobulin domain (Ig), transmembrane domain (TM) and short cytoplasmic domain, (semaphorin) 4B	Sema4b	293042	2.06	3.76	5.9E-01	1.1E-02	0.00	0.00	0.70	0.25
1372889_at	Transcribed locus			1.84	3.76	4.2E-01	5.7E-03	0.00	0.00	0.50	0.00
1373658_at	Rac GTPase-activating protein 1 (predicted)	Racgap1_predicted	315298	1.49	3.75	6.2E-01	1.0E-03	0.20	0.00	1.00	0.63
1373026_at	spindle pole body component 24 homolog (S. cerevisiae) (predicted)	Spbc24_predicted	363028	0.49	3.75	6.4E-01	1.1E-03	0.00	0.00	0.80	0.00
1372893_at	Yip1 domain family, member 1	Yipf1	298312	1.16	3.74	6.8E-01	1.5E-04	1.00	0.75	1.00	0.75
1368655_at	proteoglycan peptide core protein	Pgsg	56782	0.99	3.74	9.9E-01	3.5E-04	1.00	1.00	1.00	1.00
1381968_at	Sema domain, transmembrane domain (TM), and cytoplasmic domain, (semaphorin) 6D (predicted)	Sema6d_predicted	311384	0.95	3.72	8.4E-01	8.1E-04	1.00	1.00	1.00	1.00
1373992_at	similar to interferon-inducible GTPase	MGC108823	307414	1.07	3.72	9.2E-01	3.0E-03	1.00	1.00	1.00	1.00
1376109_at	Transcribed locus			1.19	3.71	6.0E-01	1.3E-03	1.00	1.00	1.00	1.00
1367721_at	syndecan 4	Sdc4	24771	1.00	3.71	1.0E+00	1.6E-04	0.50	0.00	1.00	0.25
1389227_at	Ras homolog gene family, member G	Rhog	308875	1.17	3.71	5.2E-01	1.7E-04	1.00	1.00	1.00	1.00
1373685_at	similar to low density lipoprotein receptor-related protein binding protein (predicted)	RGD1565715_predicted	361149	0.67	3.71	6.9E-01	1.7E-03	0.00	0.00	0.80	0.00
1372043_at	similar to ribosomal protein P0-like protein; 60S acidic ribosomal protein PO; ribosomal protein, large, P0-like (predicted)	RGD1311709_predicted	298586	1.13	3.70	9.3E-01	2.0E-03	0.20	0.00	1.00	0.00
1374799_at	similar to mKIAA0159 protein (predicted)	RGD1562596_predicted	362438	0.82	3.68	6.2E-01	4.3E-02	0.20	0.00	1.00	0.13
1387087_at	CCAAT/enhancer binding protein (C/EBP), beta	Cebpb	24253	1.08	3.68	8.5E-01	7.4E-04	1.00	1.00	1.00	1.00
1368430_at	legumain	Lgmn	63865	1.21	3.68	3.6E-01	2.1E-04	1.00	1.00	1.00	1.00
1376728_at	Hypothetical RNA binding protein RGD1359713	RGD1359713	305340	0.88	3.68	6.9E-01	5.7E-04	0.40	0.13	1.00	0.38
1375422_at				1.81	3.67	3.3E-01	3.9E-03	1.00	1.00	1.00	1.00
1390022_at	actin related protein 2/3 complex, subunit 5	Arpc5	360854	1.25	3.67	2.1E-01	4.4E-04	1.00	1.00	1.00	1.00
1371248_at	similar to Cornifin A (Small proline-rich protein 1A) (SPR1A) (SPRR1)	LOC499660	499660	1.86	3.66	4.1E-01	5.8E-02	0.00	0.50	1.00	0.00
1386870_at	glutamate-ammonia ligase (glutamine synthase)	Glul	24957	1.15	3.65	6.4E-01	2.0E-04	1.00	1.00	1.00	1.00
1369793_a_at	melanoma cell adhesion molecule	Mcam	78967	1.59	3.65	3.1E-01	3.0E-03	1.00	0.63	1.00	1.00
1373949_at	ATPase type 13A2 (predicted)	Atp13a2_predicted	362645	1.07	3.63	8.4E-01	2.9E-03	0.00	0.00	1.00	0.38
1376850_a_at	chemokine (C-C motif) ligand 27 (predicted)	Ccl27_predicted	362505	1.22	3.63	8.3E-01	1.6E-02	0.00	0.00	1.00	0.88
1371402_at	ATPase, H transporting, lysosomal V1 subunit B2	Atp6v1b2	117596	1.02	3.63	9.5E-01	3.1E-04	1.00	1.00	1.00	1.00
1373554_at	splA/ryanodine receptor domain and SOCS box containing 1 (predicted)	Spsb1_predicted	313722	0.94	3.63	9.7E-01	2.5E-04	0.00	0.00	1.00	0.25
1387135_at	a disintegrin and metallopeptidase domain 15 (metargidin)	Adam15	57025	0.97	3.62	9.2E-01	1.2E-04	1.00	1.00	1.00	1.00
1389525_at	ring finger protein 149	Rnf149	363222	0.95	3.62	8.6E-01	1.5E-04	1.00	1.00	1.00	1.00
1374529_at	Thrombospondin 1	Thbs1	445442	1.31	3.62	7.3E-01	3.3E-03	1.00	1.00	1.00	1.00
1389668_at	spindle pole body component 25 homolog (S. cerevisiae)	Spbc25	295661	1.00	3.62	1.0E+00	1.4E-03	0.00	0.50	1.00	0.75
1370530_a_at	phospholipase D1	Pld1	25096	1.19	3.62	7.6E-01	4.6E-04	0.40	0.50	1.00	1.00
1384934_at	Clone GDY20 unknown mRNA			0.91	3.62	7.2E-01	1.5E-03	0.00	0.25	1.00	0.88
1368754_at	pyrimidinergic receptor P2Y, G-protein coupled, 6	P2ry6	117264	0.98	3.60	9.4E-01	2.6E-03	0.50	0.00	1.00	0.75
1370073_at	DnaJ (Hsp40) homolog, subfamily C, member 3	Dnajc3	63880	0.92	3.60	8.5E-01	1.6E-03	1.00	1.00	1.00	1.00
1369562_at	hippocalcin-like 1	Hpcal1	50871	1.89	3.60	3.6E-01	7.1E-03	0.20	0.00	1.00	0.38
1368976_at	CD38 antigen	Cd38	25668	0.99	3.60	9.7E-01	5.3E-03	1.00	0.50	1.00	0.63
1368045_at	solute carrier family 31 (copper transporters), member 1	Slc31a1	171135	1.43	3.59	6.9E-01	4.5E-03	0.00	0.00	0.60	0.00
1373262_at	similar to 2310014H01Rik protein (predicted)	RGD1309543_predicted	361790	1.07	3.59	9.2E-01	4.8E-04	0.00	0.00	1.00	0.88
1377002_at	Interferon (alpha and beta) receptor 1 (predicted)	Ifnar1_predicted	288264	0.91	3.59	8.1E-01	1.6E-03	0.00	0.00	1.00	0.25
1368647_at	G protein-coupled receptor kinase 6	Gprk6	59076	1.13	3.58	9.4E-01	3.7E-03	0.00	0.00	1.00	0.00

Probe Set ID	Gene Title	Gene Symbol	Entrez Gene ID	Fold Change, Strain Effect: Native BN / DA	Fold Change, Rejection Effect: Allograft / Isograft	False Discovery Rate: Native BN / DA	False Discovery Rate: Allograft / Isograft	Average Present Call: Native BN	Average Present Call: Native DA	Average Present Call: Allograft	Average Present Call: Isograft
1388762_at	IQ motif containing GTPase activating protein 1 (predicted)	Iqgap1_predicted	361598	1.05	3.58	8.0E-01	2.0E-04	1.00	1.00	1.00	1.00
1377172_at	similar to Pins (predicted)	RGD1560967_predicted	362021	1.28	3.58	8.4E-01	8.3E-02	0.00	0.00	0.70	0.00
1392407_at	similar to RIKEN cDNA 6030405P05 gene (predicted)	RGD1561419_predicted	498282	1.01	3.57	9.9E-01	1.5E-03	0.80	0.25	1.00	1.00
1373881_at	Rho, GDP dissociation inhibitor (GDI) beta	Arhgdib	362456	0.87	3.56	5.0E-01	1.4E-04	1.00	1.00	1.00	1.00
1368555_at	CD37 antigen	Cd37	29185	1.15	3.56	8.1E-01	3.8E-03	0.10	0.00	1.00	0.75
1368186_a_at	spleen tyrosine kinase	Syk	25155	1.50	3.56	3.0E-01	1.8E-04	0.00	0.00	1.00	0.50
1385903_at	Zinc finger, SWIM domain containing 6	Zswim6	310062	0.71	3.56	7.8E-01	5.7E-04	0.00	0.00	0.50	0.00
1373657_at	solute carrier family 31, member 2	Slc31a2	298091	1.05	3.55	9.2E-01	1.5E-03	1.00	1.00	1.00	1.00
1377759_at	Transcribed locus			0.94	3.54	8.8E-01	3.4E-04	1.00	0.63	1.00	1.00
1368683_at	oxidized low density lipoprotein (lectin-like) receptor 1	Oldlr1	140914	0.64	3.54	7.1E-01	7.0E-03	0.00	0.00	1.00	0.25
1373672_at	Biotinidase	Btd	306262	0.97	3.54	9.5E-01	9.2E-04	0.80	1.00	1.00	1.00
1368878_at	isopentenyl-diphosphate delta isomerase	Idi1	89784	1.54	3.52	2.5E-01	1.1E-03	1.00	0.75	1.00	0.75
1387198_at	inositol polyphosphate-5-phosphatase D	Inpp5d	54259	1.02	3.50	9.6E-01	1.1E-02	1.00	1.00	1.00	1.00
1399050_at	adenylosuccinate synthetase, non muscle (predicted)	Adss_predicted	289276	0.95	3.50	8.6E-01	2.0E-04	1.00	1.00	1.00	1.00
1373224_at	ST3 beta-galactoside alpha-2,3-sialyltransferase 4	St3gal4	363040	1.72	3.49	5.3E-01	2.2E-03	0.00	0.00	1.00	0.00
1375857_at	similar to Myoferlin (Fer-1 like protein 3) (predicted)	RGD1564216_predicted	309499	1.56	3.49	6.4E-01	7.9E-04	0.00	0.00	1.00	0.50
1370516_at	solute carrier family 15, member 3	Slc15a3	246239	0.76	3.48	2.7E-01	1.2E-03	1.00	1.00	1.00	1.00
1374468_at	myeloid differentiation primary response gene 88	Myd88	301059	1.09	3.48	8.6E-01	1.8E-04	1.00	1.00	1.00	1.00
1372032_at	Transcribed locus			0.80	3.48	6.9E-01	2.0E-03	1.00	1.00	1.00	1.00
1389496_at	A kinase (PRKA) anchor protein 7	Akap7	361458	0.82	3.47	2.7E-01	6.5E-04	1.00	1.00	1.00	1.00
1388482_at	similar to RIKEN cDNA 9130404D14	RGD1307018	362115	1.12	3.47	8.4E-01	1.6E-04	1.00	1.00	1.00	1.00
1370043_at	activated leukocyte cell adhesion molecule	Alcam	79559	1.00	3.46	1.0E+00	1.0E-03	1.00	1.00	1.00	1.00
1370365_at	glutathione synthetase	Gss	25458	1.17	3.45	8.7E-01	1.3E-02	0.00	0.00	1.00	0.00
1372097_at	Transcribed locus, strongly similar to NP_543171.1 apoptotic inhibitory molecule [Rattus norvegicus]			1.27	3.45	6.3E-01	1.5E-03	1.00	0.38	1.00	1.00
1369268_at	activating transcription factor 3	Atf3	25389	1.04	3.44	9.8E-01	1.2E-02	0.00	0.00	1.00	0.75
1377092_at	Transcribed locus			1.68	3.44	3.1E-01	1.4E-03	1.00	1.00	1.00	1.00
1370348_at	ninjurin 1	Ninj1	25338	0.94	3.43	8.3E-01	1.9E-04	1.00	1.00	1.00	1.00
1371539_at	nucleolar protein family A, member 2 (predicted)	Nola2_predicted	287273	1.16	3.43	7.4E-01	9.6E-04	1.00	1.00	1.00	1.00
1370347_at	PDZ and LIM domain 7	Pdlim7	286908	0.99	3.43	9.9E-01	1.1E-03	0.30	0.00	1.00	1.00
1389756_at	maternal embryonic leucine zipper kinase (predicted)	Melk_predicted	362510	1.20	3.43	7.5E-01	3.7E-03	0.00	0.13	1.00	0.50
1368066_at	BCL2-antagonist/killer 1	Bak1	116502	0.67	3.40	4.6E-01	1.5E-03	0.20	0.25	1.00	0.75
1368762_at	ubiquitin D	Ubd	29168	0.62	3.40	2.8E-01	3.6E-02	0.30	0.50	1.00	1.00
1374556_at	spermine oxidase (predicted)	Smox_predicted	308652	0.77	3.40	3.3E-01	5.3E-04	1.00	1.00	1.00	1.00
1391880_at	tumor protein D52 (predicted)	Tpd52_predicted	294900	1.35	3.40	4.6E-01	2.4E-04	1.00	1.00	1.00	1.00
1367973_at	chemokine (C-C motif) ligand 2	Ccl2	24770	0.80	3.39	6.2E-01	7.4E-04	0.10	0.00	1.00	1.00
1388182_at	DNA primase, p49 subunit	Prim1	246327	1.34	3.37	5.1E-01	5.2E-04	0.40	0.00	1.00	1.00
1369940_at	transaldolase 1	Taldo1	83688	1.15	3.37	6.5E-01	2.9E-03	1.00	0.75	1.00	1.00
1376481_at	A disintegrin-like and metalloprotease (reprolysin type) with thrombospondin type 1 motif, 9 (predicted)	Adamts9_predicted	312566	1.76	3.37	7.4E-01	2.2E-03	0.00	0.00	1.00	0.00
1371977_at	actin related protein 2/3 complex, subunit 3 (predicted)	Arpc3_predicted	288669	0.98	3.36	9.6E-01	1.9E-04	1.00	1.00	1.00	1.00
1373959_at	protein phosphatase 2 (formerly 2A), regulatory subunit A (PR 65), beta isoform	Ppp2r1b	315648	2.12	3.36	3.5E-01	7.6E-04	0.00	0.00	1.00	0.00
1376636_at	transforming growth factor, beta receptor 1	Tgfbr1	29591	1.21	3.35	6.3E-01	9.9E-04	1.00	0.88	1.00	1.00
1373138_at	nudix (nucleoside diphosphate linked moiety X)-type motif 5	Nudt5	361274	2.09	3.34	2.7E-01	1.9E-04	0.00	0.00	1.00	0.50
1376693_at	similar to OEF2 (predicted)	RGD1563091_predicted	500011	1.07	3.34	8.8E-01	7.5E-03	1.00	0.50	1.00	1.00
1394316_a_at	tetraspanin 5	Tspan5	362048	0.72	3.34	7.7E-01	3.0E-02	0.00	0.00	1.00	0.00
1369456_at	5-hydroxytryptamine (serotonin) receptor 2B	Htr2b	29581	1.54	3.34	6.6E-01	4.0E-03	0.60	0.25	1.00	0.75
1373869_at	sterol O-acyltransferase 1	Soat1	81782	1.44	3.33	4.3E-01	1.2E-03	0.00	0.00	1.00	0.25
1374626_at	leucine-rich alpha-2-glycoprotein 1	Lrg1	367455	1.18	3.33	5.8E-01	9.9E-04	1.00	0.25	1.00	0.75
1373592_at	similar to SPI6	MGC94010	361241	1.21	3.33	5.0E-01	1.8E-03	0.80	1.00	1.00	1.00
1398349_at	adenylate kinase 2	Ak2	24184	0.69	3.32	2.5E-01	2.8E-04	1.00	1.00	1.00	1.00
1376637_at	Transcribed locus			1.07	3.32	9.7E-01	9.7E-02	0.00	0.25	0.80	0.00
1375170_at	S100 calcium binding protein A11 (calizzarin)	S100a11	445415	1.12	3.31	7.0E-01	3.7E-04	1.00	1.00	1.00	1.00
1367712_at	tissue inhibitor of metallopeptidase 1	Timp1	116510	1.37	3.31	5.7E-01	2.6E-02	0.80	0.63	1.00	1.00
1368914_at	runt related transcription factor 1	Runx1	50662	2.39	3.30	2.1E-01	3.3E-02	0.00	0.00	0.80	0.25
1388071_x_at	RT1 class Ib, locus Aw2	RT1-Aw2	24737	1.65	3.29	1.9E-01	7.5E-04	1.00	1.00	1.00	1.00
1371537_at	Transcribed locus			1.15	3.29	7.6E-01	7.9E-04	0.90	1.00	1.00	1.00
1374578_at	Transcribed locus			1.24	3.29	3.3E-01	4.2E-04	0.80	0.88	1.00	1.00
1374449_at	Solute carrier family 35, member E1 (predicted)	Slc35e1_predicted	306328	0.85	3.28	5.9E-01	1.5E-03	0.60	1.00	1.00	0.88
1373536_at				0.90	3.28	8.4E-01	4.5E-04	1.00	0.75	1.00	1.00
1368948_at	moesin	Msn	81521	1.55	3.28	2.7E-01	3.2E-02	0.50	0.25	1.00	0.50
1370294_a_at	cell division cycle 20 homolog (S. cerevisiae)	Cdc20	64515	0.86	3.28	7.8E-01	3.7E-04	0.00	0.00	1.00	0.25
1389873_at	PYD and CARD domain containing	Pycard	282817	1.07	3.27	6.6E-01	1.9E-03	1.00	1.00	1.00	1.00
1371922_at	Transcribed locus			0.86	3.27	7.7E-01	3.1E-02	0.00	0.88	1.00	0.13
1367914_at	epithelial membrane protein 3	Emp3	81505	0.82	3.27	6.1E-01	1.8E-04	1.00	1.00	1.00	1.00
1367936_at	serine/threonine kinase 10	Stk10	29398	1.22	3.27	5.2E-01	1.9E-03	1.00	1.00	1.00	1.00
1371232_a_at	chondroitin sulfate proteoglycan 2	Cspg2	114122	0.98	3.27	9.9E-01	3.7E-02	0.00	0.00	1.00	0.50
1376842_at	Transcribed locus			1.32	3.27	5.4E-01	2.0E-03	0.80	0.25	1.00	0.50
1390391_at	Transcribed locus			1.13	3.26	7.2E-01	1.1E-03	0.80	1.00	1.00	1.00
1374458_at	Transcribed locus, strongly similar to XP_001060531.1 similar to serum/glucocorticoid regulated kinase 3			1.18	3.25	8.2E-01	6.6E-04	1.00	1.00	1.00	1.00
1370972_x_at	RT1 class I, CE5	RT1-CE5	309607	0.71	3.25	3.6E-01	1.4E-02	0.00	0.00	1.00	0.00
1389742_at	Transcribed locus			1.22	3.25	8.2E-01	1.0E-02	0.20	0.25	1.00	0.50
1367780_at	pituitary tumor-transforming 1	Pttg1	64193	0.99	3.24	9.9E-01	1.2E-03	0.00	0.00	0.60	0.00
1372132_at	CNDP dipeptidase 2 (metallopeptidase M20 family)	Cndp2	291394	1.12	3.23	6.7E-01	2.1E-04	1.00	1.00	1.00	1.00
1374431_at	similar to ATPase, H+ transporting, V1 subunit A, isoform 1	LOC685232	685232	1.22	3.23	6.5E-01	4.7E-04	1.00	1.00	1.00	1.00
1392819_at	Transcribed locus			1.31	3.22	7.0E-01	1.2E-03	0.10	0.00	1.00	0.63
1375696_at	interferon (alpha and beta) receptor 1 (predicted)	Ifnar1_predicted	288264	1.22	3.21	7.5E-01	5.8E-03	1.00	1.00	1.00	1.00
1388440_at	anterior pharynx defective 1b homolog (C. elegans)	Aph1b	300802	0.88	3.21	4.2E-01	2.4E-04	1.00	1.00	1.00	1.00
1388203_x_at	RT1 class Ib, locus Aw2	RT1-Aw2	24737	1.01	3.21	9.8E-01	2.7E-04	1.00	1.00	1.00	1.00

				Fold Change		False Discovery Rate		Average Present Call			
				Strain Effect	Rejection Effect						
Probe Set ID	Gene Title	Gene Symbol	Entrez Gene ID	Native BN / DA	Allograft / Isograft	Native BN / DA	Allograft / Isograft	Native BN	Native DA	Allograft	Isograft
1370539_at	RAB8B, member RAS oncogene family	Rab8b	266688	1.67	3.21	1.5E-01	4.2E-03	1.00	1.00	1.00	1.00
1387946_at	lectin, galactoside-binding, soluble, 3 binding protein	Lgals3bp	245955	0.91	3.20	7.7E-01	5.0E-04	1.00	1.00	1.00	1.00
1370184_at	cofilin 1, non-muscle	Cfl1	29271	0.94	3.19	9.1E-01	1.9E-03	1.00	1.00	1.00	1.00
1390386_at	caspase 3, apoptosis related cysteine protease	Casp3	25402	1.20	3.18	9.2E-01	1.5E-03	0.00	0.00	1.00	0.00
1371644_at	protein tyrosine kinase 9	Ptk9	315265	0.62	3.17	7.4E-01	1.7E-04	0.00	0.00	1.00	1.00
1369122_at	Bcl2-associated X protein	Bax	24887	0.99	3.17	9.7E-01	2.8E-03	0.10	0.50	1.00	1.00
1376793_at	similar to RIKEN cDNA 5730414C17 (predicted)	RGD1308377_predicted	306687	0.89	3.16	6.2E-01	3.3E-04	1.00	1.00	1.00	1.00
1370082_at	transforming growth factor, beta 1	Tgfb1	59086	1.30	3.16	5.8E-01	7.7E-04	0.00	0.00	1.00	0.00
1374565_at	NIMA (never in mitosis gene a)-related expressed kinase 6	Nek6	360161	1.17	3.16	6.4E-01	6.8E-04	1.00	0.00	1.00	0.75
1372691_at	uridine phosphorylase 1	Upp1	289801	0.91	3.16	8.3E-01	5.0E-04	1.00	1.00	1.00	1.00
1371618_s_at	tubulin, beta 3	Tubb3	246118	0.99	3.15	9.9E-01	7.3E-03	0.00	0.00	1.00	1.00
1373751_at	Transcribed locus			1.49	3.15	1.8E-01	4.4E-04	1.00	0.50	1.00	1.00
1375020_at	Ras and Rab interactor 3 (predicted)	Rin3_predicted	314397	1.21	3.14	3.0E-01	2.6E-03	0.00	0.00	1.00	0.50
1374615_at				1.61	3.14	4.9E-01	1.3E-02	0.60	0.50	1.00	1.00
1372513_at	ras-related C3 botulinum toxin substrate 1	Rac1	363875	1.70	3.14	7.3E-02	3.8E-03	1.00	1.00	1.00	1.00
1375420_at	tumor protein p53 inducible protein 11 (predicted)	Tp53i11_predicted	311209	0.98	3.13	9.7E-01	1.9E-04	0.40	0.13	1.00	0.88
1373198_at	similar to RIKEN cDNA 2810451A06	RGD1311098	362998	1.46	3.13	2.0E-01	2.5E-03	0.20	0.00	1.00	0.00
1383912_at	similar to Protein C6orf115	LOC684848	684848	0.84	3.13	3.1E-01	6.9E-03	1.00	1.00	1.00	1.00
1367745_at	diacylglycerol kinase zeta	Dgkz	81821	1.09	3.12	8.4E-01	7.0E-03	0.20	0.00	1.00	0.00
1390563_at	ATP-binding cassette, sub-family A (ABC1), member 3	Abca3	302973	1.54	3.12	6.2E-01	2.8E-03	0.00	0.00	1.00	0.00
1376722_at	nucleoporin 205kDa (predicted)	Nup205_predicted	362335	1.47	3.12	3.3E-01	1.9E-04	0.90	0.00	1.00	1.00
1371368_at	Sec61 alpha 1 subunit (S. cerevisiae)	Sec61a1	80843	1.42	3.12	4.9E-01	4.6E-04	0.70	0.25	1.00	1.00
1372156_at	transmembrane protein 97	Tmem97	303330	1.11	3.12	6.9E-01	5.7E-04	0.00	0.00	1.00	1.00
1370499_at	killer cell lectin-like receptor subfamily B, member 1A (mapped)	Klrb1a	362443	0.86	3.12	8.1E-01	6.6E-02	0.80	0.75	1.00	0.50
1387402_at	myosin, heavy polypeptide 9, non-muscle	Myh9	25745	0.99	3.12	9.9E-01	8.5E-02	0.00	0.00	0.60	0.00
1374912_at	Alpha-2u globulin PGCL1	LOC259246	259246	0.92	3.12	9.4E-01	4.9E-02	0.00	0.00	0.60	0.00
1370096_at	perforin 1 (pore forming protein)	Prf1	50669	1.16	3.12	9.4E-01	1.4E-03	0.00	0.00	0.60	0.00
1374933_at	melanoma cell adhesion molecule	Mcam	78967	1.84	3.12	4.0E-01	1.3E-03	1.00	1.00	1.00	1.00
1372182_at	phosphofructokinase, platelet	Pfkp	60416	1.17	3.11	6.7E-01	5.7E-03	1.00	1.00	1.00	1.00
1370954_at	procollagen-proline, 2-oxoglutarate 4-dioxygenase (proline 4-hydroxylase), alpha 1 polypeptide	P4ha1	64475	1.04	3.11	9.3E-01	1.8E-04	1.00	1.00	1.00	1.00
1388857_at	SEC23B (S. cerevisiae) (predicted)	Sec23b_predicted	362226	1.34	3.11	3.6E-01	6.5E-04	1.00	1.00	1.00	1.00
1386976_at	CD82 antigen	Cd82	83628	1.10	3.11	8.2E-01	1.4E-03	0.60	0.38	1.00	1.00
1368437_at	carbonic anhydrase 4	Ca4	29242	0.34	3.10	2.2E-01	7.2E-03	0.00	0.00	0.90	0.00
1388745_at	sema domain, immunoglobulin domain (Ig), transmembrane domain (TM) and short cytoplasmic domain, (semaphorin) 4A	Sema4a	310630	1.01	3.10	9.7E-01	9.3E-04	1.00	0.75	1.00	1.00
1388913_at	phosphatidic acid phosphatase type 2c	Ppap2c	246115	1.24	3.10	8.8E-01	7.2E-02	0.00	0.00	0.70	0.00
1375424_at	ARP2 actin-related protein 2 homolog (yeast)	Actr2	289820	1.25	3.10	4.9E-01	1.3E-04	1.00	1.00	1.00	1.00
1367740_at	creatine kinase, brain	Ckb	24264	1.23	3.10	5.3E-01	2.9E-03	1.00	1.00	1.00	1.00
1383906_at	similar to lung inducible neuralized-related C3HC4 RING finger protein	LOC316326	316326	1.63	3.09	5.3E-01	5.2E-04	0.00	0.00	1.00	0.00
1380546_at	similar to hypothetical protein FLJ10986	LOC298250	298250	0.72	3.09	4.1E-01	4.0E-03	1.00	1.00	1.00	1.00
1388600_at	CDNA clone IMAGE:7320582			1.51	3.08	3.3E-01	1.5E-03	1.00	0.88	1.00	1.00
1377353_a_at	tumor necrosis factor (ligand) superfamily, member 13	Tnfsf13	287437	1.04	3.08	8.5E-01	2.7E-03	0.20	0.13	1.00	0.75
1388347_at	similar to lymphocyte antigen 6 complex, locus E	LOC362934	362934	0.72	3.08	2.8E-01	2.1E-03	1.00	1.00	1.00	1.00
1389380_at	similar to Folylpolyglutamate synthase, mitochondrial precursor	LOC687266	687266	1.36	3.08	7.3E-01	4.2E-03	0.00	0.00	1.00	0.00
1373043_at	similar to stromal cell-derived factor 2-like 1	LOC680945	680945	1.06	3.08	9.0E-01	7.2E-04	1.00	1.00	1.00	1.00
1368305_at	caspase 6	Casp6	83584	1.39	3.07	5.5E-01	4.7E-02	0.00	0.00	0.60	0.00
1370631_at	regenerating islet-derived 3 gamma	Reg3g	24620	4.48	3.07	1.1E-01	6.5E-02	1.00	0.75	1.00	1.00
1376624_at	Transcribed locus			1.02	3.07	9.6E-01	3.6E-04	1.00	0.75	1.00	1.00
1377821_at	Transcribed locus			0.92	3.07	9.2E-01	4.4E-05	1.00	1.00	1.00	1.00
1388416_at	low density lipoprotein receptor-related protein 1	Lrp1	299858	2.73	3.07	3.0E-01	3.4E-03	1.00	0.25	1.00	1.00
1373221_at	diaphanous homolog 1 (Drosophila) (predicted)	Diap1_predicted	307483	1.28	3.06	6.3E-01	6.4E-04	1.00	0.75	1.00	1.00
1398892_at	Niemann Pick type C2	Npc2	286898	1.02	3.06	9.7E-01	1.3E-03	1.00	1.00	1.00	1.00
1398319_at	Nclone10 mRNA			1.12	3.06	7.8E-01	2.1E-03	1.00	0.88	1.00	1.00
1372770_at				1.55	3.05	1.9E-01	5.3E-04	1.00	1.00	1.00	1.00
1398360_at	similar to Elongation of very long chain fatty acids protein 1	LOC679532	679532	0.87	3.04	7.4E-01	1.4E-04	1.00	1.00	1.00	1.00
1387871_at	cofilin 1, non-muscle	Cfl1	29271	0.92	3.04	8.8E-01	2.5E-03	0.70	0.75	1.00	1.00
1374939_at	cytoplasmic FMR1 interacting protein 2 (predicted)	Cyfip2_predicted	303073	0.80	3.04	5.4E-01	5.2E-03	0.60	0.63	1.00	0.75
1373164_at	Transcribed locus			1.10	3.04	8.6E-01	3.3E-04	1.00	1.00	1.00	1.00
1370999_at	sperm associated antigen 5	Spag5	252918	1.71	3.04	5.5E-01	1.4E-02	0.00	0.00	0.50	0.00
1376697_at	carbohydrate sulfotransferase 12	Chst12	304322	0.86	3.03	8.3E-01	6.5E-04	0.00	0.00	1.00	0.00
1388393_at	proteolipid protein 2 (mapped)	Plp2	302562	1.14	3.03	5.6E-01	1.8E-04	1.00	1.00	1.00	1.00
1377260_at	Transcribed locus			0.74	3.03	4.5E-01	8.6E-03	0.00	0.13	1.00	0.25
1371027_at	Casitas B-lineage lymphoma b	Cblb	171136	0.95	3.03	9.1E-01	2.5E-03	1.00	1.00	1.00	1.00
1370822_at	RT1 class II, locus Ba	RT1-Ba	309621	1.08	3.03	9.3E-01	8.3E-03	0.00	0.00	1.00	1.00
1369262_at	caspase 8	Casp8	64044	0.90	3.02	9.3E-01	3.6E-04	0.00	0.00	1.00	0.00
1374876_at	leptin receptor overlapping transcript-like 1	Leprotl1	361160	0.84	3.02	4.8E-01	2.6E-03	0.90	0.88	1.00	1.00
1387195_at	suppression of tumorigenicity 14 (colon carcinoma)	St14	114093	1.05	3.02	9.3E-01	1.4E-03	1.00	0.75	1.00	1.00
1370297_at	polo-like kinase 1 (Drosophila)	Plk1	25515	1.04	3.02	9.4E-01	3.2E-02	0.00	0.00	0.60	0.00
1376657_at	immunoglobulin superfamily, member 4A	Igsf4a	363058	0.61	3.02	5.9E-01	2.4E-02	0.00	0.00	1.00	0.25
1372459_at	vasodilator-stimulated phosphoprotein (predicted)	Vasp_predicted	361517	1.12	3.02	7.2E-01	2.6E-04	1.00	1.00	1.00	1.00
1376317_at	origin recognition complex, subunit 6-like (S. cerevisiae)	Orc6l	291927	0.89	3.02	8.6E-01	1.3E-03	0.00	0.00	1.00	0.00
1370051_at	transglutaminase 1	Tgm1	60335	2.92	3.01	3.3E-01	2.9E-02	0.00	0.00	0.70	0.00
1379598_at	Transcribed locus			1.13	3.01	9.4E-01	1.6E-03	0.00	0.00	1.00	0.75
1371936_at	eukaryotic translation initiation factor 4A1	Eif4a1	287436	0.98	3.01	9.6E-01	2.4E-04	1.00	1.00	1.00	1.00
1388566_at	LIM and SH3 protein 1	Lasp1	29278	1.18	3.01	2.9E-01	3.8E-04	1.00	1.00	1.00	1.00
1373831_at	hypothetical protein LOC619574	LOC619574	619574	0.85	3.01	8.2E-01	4.4E-04	0.00	0.00	1.00	0.00
1388750_at	transferrin receptor	Tfrc	64678	0.59	3.00	3.2E-01	2.1E-04	1.00	1.00	1.00	1.00
1388151_at	coronin 7	Coro7	192276	0.96	3.00	9.3E-01	1.9E-04	0.40	0.13	1.00	0.75

				Fold Change		False Discovery Rate		Average Present Call			
				Strain Effect	Rejection Effect						
Probe Set ID	Gene Title	Gene Symbol	Entrez Gene ID	Native BN / DA	Allograft / Isograft	Native BN / DA	Allograft / Isograft	Native BN	Native DA	Allograft	Isograft
1374320_at	coagulation factor 5 (mapped)	F5	304929	1.30	3.00	3.4E-01	3.9E-04	1.00	1.00	1.00	1.00
1367938_at	UDP-glucose dehydrogenase	Ugdh	83472	1.27	3.00	3.0E-01	9.5E-03	1.00	1.00	1.00	1.00
1376169_at	similar to PHD finger protein 20-like 1 isoform 1	LOC314964	314964	1.02	0.33	9.8E-01	5.5E-02	1.00	1.00	0.70	1.00
1368252_at	kelch repeat and BTB (POZ) domain containing 10	Kbtbd10	117537	1.21	0.33	3.4E-01	4.3E-03	1.00	1.00	1.00	1.00
1370918_a_at	ATP synthase, H+ transporting, mitochondrial F1 complex, gamma polypeptide 1	Atp5c1	116550	0.89	0.33	7.4E-01	5.0E-03	1.00	1.00	1.00	1.00
1377054_at	Transcribed locus			1.02	0.33	9.8E-01	1.3E-03	1.00	1.00	0.00	1.00
1375640_at	FK506 binding protein 9	Fkbp9	297123	0.96	0.33	9.0E-01	5.2E-04	0.80	1.00	1.00	1.00
1388112_at	solute carrier family 25 (mitochondrial carrier; adenine nucleotide translocator), member 4	Slc25a4	85333	0.85	0.33	6.3E-01	2.8E-03	1.00	1.00	1.00	1.00
1373249_at	Transcribed locus			0.86	0.33	6.5E-01	1.3E-03	1.00	1.00	1.00	1.00
1372054_at	Transcribed locus			1.08	0.33	8.1E-01	5.2E-03	1.00	1.00	1.00	1.00
1383175_a_at	similar to C11orf17 protein (predicted)	RGD1306959_predicted	361624	1.24	0.33	5.5E-01	9.9E-03	1.00	1.00	1.00	1.00
1389805_at				0.17	0.33	7.6E-02	1.5E-02	0.00	0.00	0.00	0.50
1376066_at	Transcribed locus			0.90	0.33	8.7E-01	2.5E-04	1.00	1.00	1.00	1.00
1367654_at	fat tumor suppressor homolog (Drosophila)	Fath	83720	1.52	0.33	1.8E-01	9.9E-04	1.00	1.00	1.00	1.00
1376584_at	Transcribed locus			1.35	0.33	3.6E-01	1.0E-03	1.00	1.00	1.00	1.00
1375197_at	Transcribed locus			0.81	0.33	2.8E-01	2.5E-03	1.00	1.00	1.00	1.00
1371293_at	similar to Myosin light polypeptide 4 (Myosin light chain 1, atrial isoform)	LOC688228	688228	1.25	0.33	4.6E-01	2.8E-03	1.00	1.00	0.00	1.00
1367726_at	thyroid hormone receptor alpha	Thra	81812	0.91	0.33	7.7E-01	1.4E-03	0.90	0.75	0.00	1.00
1373239_at	SH3 and PX domain containing 3 (predicted)	Sh3px3_predicted	315696	0.74	0.33	4.8E-01	6.1E-03	0.30	0.50	0.00	1.00
1371793_at	Acetylcholinesterase	Ache	83817	0.94	0.33	8.5E-01	1.9E-03	1.00	1.00	0.80	1.00
1370060_at	solute carrier family 25 (mitochondrial carrier; oxoglutarate carrier), member 11	Slc25a11	64201	0.97	0.33	9.3E-01	1.4E-03	1.00	1.00	1.00	1.00
1372612_at	dynein light chain LC8-type 2	Dynll2	140734	0.84	0.33	3.8E-01	1.1E-03	1.00	1.00	1.00	1.00
1370447_at	phospholipase B	Phlpb	192259	1.22	0.33	7.4E-01	3.9E-02	1.00	1.00	0.80	1.00
1372296_at	similar to putative SH3BGR protein (predicted)	RGD1563599_predicted	498066	0.82	0.33	4.7E-01	1.3E-02	1.00	1.00	1.00	1.00
1387898_at	heat shock protein, alpha-crystallin-related, B6	Hspb6	192245	1.03	0.33	9.5E-01	3.8E-02	1.00	1.00	1.00	1.00
1389918_at	Similar to AL023001 protein (predicted)	RGD1305001_predicted	304714	0.53	0.33	4.1E-03	1.0E-03	0.90	1.00	1.00	1.00
1373134_at	fumarylacetoacetate hydrolase domain containing 2A (predicted)	Fahd2a_predicted	296131	0.99	0.33	9.8E-01	3.2E-03	1.00	1.00	0.60	1.00
1375411_at	NADH dehydrogenase (ubiquinone) 1 alpha subcomplex, 7 (B14.5a) (predicted)	Ndufa7_predicted	299643	0.74	0.33	1.7E-01	1.0E-02	1.00	1.00	1.00	1.00
1375628_at	NADH dehydrogenase (ubiquinone) 1 beta subcomplex 8 (predicted)	Ndufb8_predicted	293991	0.75	0.32	4.8E-01	1.2E-02	1.00	1.00	0.50	1.00
1376924_a_at	palmdelphin	Palmd	310811	0.90	0.32	8.3E-01	7.7E-03	1.00	1.00	0.10	1.00
1371871_at	RAB12, member RAS oncogene family	Rab12	25530	1.04	0.32	8.7E-01	1.2E-03	1.00	1.00	1.00	1.00
1388936_at	cadherin 11	Cdh11	84407	1.43	0.32	2.7E-01	1.1E-02	1.00	1.00	0.80	1.00
1388437_at	Transcribed locus			1.25	0.32	4.1E-01	2.0E-04	1.00	1.00	1.00	1.00
1371346_at	NADH dehydrogenase (ubiquinone) 1 beta subcomplex, 6 (predicted)	Ndufb6_predicted	297990	0.79	0.32	4.0E-01	8.5E-03	1.00	1.00	1.00	1.00
1388200_at	myosin regulatory light chain 2, ventricular/cardiac muscle isoform	Mlc2	363925	0.84	0.32	6.6E-01	2.6E-02	1.00	1.00	1.00	1.00
1368818_at	proteasome (prosome, macropain) activator subunit 4	Psme4	498433	1.18	0.32	2.8E-01	7.6E-04	1.00	1.00	1.00	1.00
1373967_at	Transcribed locus			1.01	0.32	9.9E-01	2.5E-03	1.00	1.00	0.20	1.00
1399130_at	similar to RIKEN cDNA 2610022G08	LOC502782	502782	0.83	0.32	5.2E-01	1.7E-03	1.00	1.00	1.00	1.00
1368281_at	dipeptidase 1 (renal)	Dpep1	94199	0.48	0.32	2.3E-02	4.8E-04	0.60	0.88	0.60	1.00
1387768_at	myoglobin	Mb	59108	0.84	0.32	5.6E-01	2.5E-02	1.00	1.00	1.00	1.00
1372586_at	Ubiquitin-conjugating enzyme E2D 3 (UBC4/5 homolog, yeast)	Ube2d3	81920	0.78	0.32	3.4E-01	5.6E-03	1.00	1.00	1.00	1.00
1390706_at	spectrin beta 2	Spnb2	305614	1.52	0.32	1.2E-01	4.3E-03	1.00	1.00	1.00	1.00
1371371_at	NADH dehydrogenase (ubiquinone) 1 beta subcomplex, 4, 15kDa	Ndufb4	288088	0.81	0.32	5.6E-01	2.6E-03	1.00	1.00	1.00	1.00
1371989_at	high mobility group nucleosomal binding domain 3	Hmgn3	113990	0.60	0.32	2.3E-01	6.7E-04	1.00	1.00	1.00	1.00
1371786_at	tripartite motif protein 35	Trim35	498538	0.88	0.32	5.8E-01	4.8E-04	1.00	1.00	1.00	1.00
1383192_at	spastin (predicted)	Spast_predicted	362700	0.94	0.32	9.0E-01	8.9E-02	0.40	0.13	0.00	0.75
1370642_s_at	platelet derived growth factor receptor, beta polypeptide	Pdgfrb	24629	1.24	0.32	6.2E-01	1.0E-02	1.00	0.75	1.00	1.00
1367604_at	cysteine-rich protein 2	Crip2	338401	0.88	0.32	6.4E-01	1.6E-03	1.00	1.00	1.00	1.00
1372799_at	deoxyguanosine kinase (predicted)	Dguok_predicted	297389	0.60	0.32	1.9E-01	2.6E-03	1.00	1.00	1.00	1.00
1375642_at	ubiquitin-conjugating enzyme E2B, RAD6 homolog (S. cerevisiae)	Ube2b	81816	1.04	0.32	9.3E-01	2.0E-03	1.00	1.00	1.00	1.00
1370238_at	RAB12, member RAS oncogene family	Rab12	25530	0.77	0.32	2.4E-01	8.0E-03	1.00	1.00	1.00	1.00
1370288_a_at	tropomyosin 1, alpha	Tpm1	24851	0.92	0.32	7.0E-01	1.9E-02	1.00	1.00	1.00	1.00
1369927_at	malate dehydrogenase 2, NAD (mitochondrial)	Mdh2	81829	0.90	0.32	7.8E-01	2.5E-03	1.00	1.00	1.00	1.00
1371738_at	similar to hypothetical protein FLJ10375 (predicted)	RGD1308260_predicted	301111	0.99	0.32	9.9E-01	2.9E-03	1.00	1.00	1.00	1.00
1388603_a_at	HESB like domain containing 2	Hbld2	290985	0.96	0.32	8.5E-01	8.9E-04	1.00	1.00	1.00	1.00
1388935_at	Transcribed locus			1.21	0.32	5.3E-01	1.6E-03	1.00	1.00	0.60	1.00
1383161_a_at				0.83	0.32	6.6E-01	7.7E-03	1.00	1.00	1.00	1.00
1367764_at	cyclin G1	Ccng1	25405	0.97	0.32	7.7E-01	2.5E-03	1.00	1.00	1.00	1.00
1370006_at	Receptor-interacting serine-threonine kinase 3	Ripk3	246240	0.95	0.32	8.9E-01	6.6E-03	1.00	1.00	1.00	1.00
1372149_at	AU RNA binding protein/enoyl-coenzyme A hydratase (predicted)	Auh_predicted	361215	0.78	0.32	2.8E-01	1.3E-03	1.00	1.00	1.00	1.00
1373142_at	Growth hormone inducible transmembrane protein	Ghitm	290596	0.94	0.32	8.3E-01	2.0E-03	1.00	1.00	1.00	1.00
1368814_at	aldehyde dehydrogenase family 6, subfamily A1	Aldh6a1	81708	1.73	0.32	2.1E-01	7.7E-04	1.00	1.00	0.70	1.00
1372934_at	similar to 1700019E19Rik protein (predicted)	RGD1307392_predicted	299209	0.91	0.32	6.4E-01	9.5E-03	1.00	1.00	1.00	1.00
1369817_at	heart and neural crest derivatives expressed transcript 2	Hand2	64637	1.08	0.32	7.7E-01	9.5E-04	1.00	1.00	0.10	1.00
1387873_at	WAP four-disulfide core domain 1	Wfdc1	171112	0.68	0.32	2.1E-01	1.4E-03	1.00	1.00	0.30	1.00
1372590_at	C1q and tumor necrosis factor related protein 1	C1qtnf1	303701	0.89	0.32	7.8E-01	6.4E-02	0.50	0.13	0.00	0.75
1369636_at	sorbitol dehydrogenase	Sord	24788	0.80	0.32	4.5E-01	2.1E-03	1.00	1.00	0.20	1.00
1373487_at	Spondin 1	Spon1	64456	2.64	0.32	1.1E-01	1.1E-02	1.00	0.38	1.00	1.00
1372299_at	cyclin-dependent kinase inhibitor 1C (P57)	Cdkn1c	246060	1.22	0.32	5.8E-01	2.6E-03	0.90	1.00	0.20	1.00
1389287_at	CDNA clone IMAGE:7193711			1.36	0.32	2.3E-01	4.0E-04	1.00	1.00	1.00	1.00
1373848_at	similar to RIKEN cDNA 5730449L18 (predicted)	RGD1308584_predicted	289083	0.72	0.31	5.0E-01	9.0E-02	1.00	1.00	0.50	1.00
1388111_at	elastin	Eln	25043	0.93	0.31	9.0E-01	6.5E-03	0.80	0.75	0.40	1.00
1371542_at	tubulin, alpha 4	Tuba4	316531	0.81	0.31	4.0E-01	1.5E-02	1.00	1.00	1.00	1.00
1370166_at	syndecan 2	Sdc2	25615	1.29	0.31	4.7E-01	1.3E-02	1.00	1.00	1.00	1.00
1373158_at	Transcribed locus			0.76	0.31	3.2E-01	1.5E-03	1.00	1.00	1.00	1.00
1373582_at	similar to CLIP-170-related protein (predicted)	RGD1306245_predicted	308493	1.32	0.31	5.4E-01	8.2E-02	0.00	0.00	0.20	0.75
1388167_at	nuclear factor I/B	Nfib	29227	1.06	0.31	8.7E-01	3.2E-04	1.00	1.00	0.80	1.00

Probe Set ID	Gene Title	Gene Symbol	Entrez Gene ID	Fold Change: Strain Effect: Native BN / DA	Fold Change: Rejection Effect: Allograft / Isograft	False Discovery Rate: Native BN / DA	False Discovery Rate: Allograft / Isograft	Average Present Call: Native BN	Average Present Call: Native DA	Average Present Call: Allograft	Average Present Call: Isograft
1372144_at	DnaJ (Hsp40) homolog, subfamily B, member 5 (predicted)	Dnajb5_predicted	313811	1.49	0.31	1.2E-01	2.1E-02	1.00	1.00	0.20	1.00
1371607_at	microtubule-associated protein 4	LOC367171	367171	1.29	0.31	6.0E-01	2.5E-03	1.00	1.00	1.00	1.00
1372849_at	Transcribed locus			0.90	0.31	8.7E-01	3.0E-02	1.00	1.00	0.70	1.00
1371349_at	procollagen, type VI, alpha 1 (predicted)	Col6a1_predicted	294337	1.06	0.31	8.7E-01	6.5E-02	1.00	1.00	1.00	1.00
1368137_at	microtubule-associated protein tau	Mapt	29477	1.06	0.31	8.3E-01	1.6E-03	0.90	0.63	0.00	1.00
1389562_at	Transcribed locus			0.79	0.31	5.6E-01	3.7E-04	0.00	0.38	0.00	1.00
1387919_at	mitofusin 2	Mfn2	64476	0.92	0.31	6.6E-01	1.6E-03	1.00	1.00	1.00	1.00
1376861_at	similar to RIKEN cDNA 1810018L05	LOC317312	317312	1.12	0.31	8.0E-01	1.1E-03	1.00	1.00	1.00	1.00
1387773_at	cytochrome c, somatic	Cycs	25309	0.84	0.31	5.2E-01	5.8E-03	1.00	1.00	1.00	1.00
1389816_at	Procollagen, type XI, alpha 2 (mapped)	Col11a2_mapped	294279	1.10	0.31	6.7E-01	1.4E-03	1.00	1.00	1.00	1.00
1370855_at	cystatin C	Cst3	25307	0.78	0.31	2.5E-01	2.4E-03	1.00	1.00	1.00	1.00
1375018_at	similar to hypothetical protein D630003M21 (predicted)	RGD1563354_predicted	311592	0.78	0.31	2.0E-01	8.8E-02	0.00	0.63	0.00	1.00
1376320_at	Transcribed locus			0.79	0.31	4.8E-01	3.5E-02	1.00	0.50	0.00	1.00
1367599_at	ATP synthase, H+ transporting, mitochondrial F0 complex, subunit c (subunit 9), isoform 1	Atp5g1	29754	0.81	0.31	4.5E-01	2.4E-03	1.00	1.00	1.00	1.00
1371691_at	retinoic acid receptor responder (tazarotene induced) 2	Rarres2	297073	1.47	0.31	3.6E-01	2.5E-02	1.00	1.00	1.00	1.00
1384240_at	angiotensin II receptor, type 1 (AT1A)	Agtr1a	24180	0.65	0.31	6.8E-01	5.5E-02	0.00	0.00	0.10	1.00
1373092_at	Transcribed locus			1.29	0.31	1.8E-01	3.0E-03	1.00	1.00	1.00	1.00
1376646_at	popeye domain containing 2	Popdc2	360718	0.81	0.31	4.8E-01	5.6E-03	1.00	1.00	1.00	1.00
1387224_at	diacylglycerol kinase, beta	Dgkb	54248	0.48	0.31	7.6E-02	1.1E-02	0.20	1.00	0.80	1.00
1374636_at	PHD finger protein 17 (predicted)	Phf17_predicted	310352	0.86	0.31	4.9E-01	3.5E-04	1.00	1.00	1.00	1.00
1367959_a_at	sodium channel, voltage-gated, type I, beta	Scn1b	29686	1.30	0.31	4.7E-01	2.1E-02	1.00	1.00	1.00	1.00
1373270_at	WD repeat domain, phosphoinositide interacting 1 (predicted)	Wipi1_predicted	303630	0.94	0.31	8.8E-01	6.9E-04	1.00	1.00	1.00	1.00
1371593_at	Proliferating cell nuclear antigen	Pcna	25737	0.98	0.31	9.7E-01	1.1E-03	1.00	1.00	1.00	1.00
1392530_at	similar to NADH dehydrogenase (ubiquinone) 1, subcomplex unknown, 1	LOC679398	679398	0.70	0.31	2.6E-01	4.0E-03	1.00	1.00	1.00	1.00
1368544_a_at	nucleolar protein 3 (apoptosis repressor with CARD domain)	Nol3	85383	1.71	0.31	1.1E-01	9.3E-04	1.00	1.00	1.00	1.00
1373839_at	Neighbor of Punc E11 (predicted)	Nope_predicted	363081	1.17	0.31	5.1E-01	3.2E-03	1.00	1.00	0.60	1.00
1374520_at	similar to heart alpha-kinase (predicted)	RGD1564431_predicted	498875	1.30	0.31	4.8E-01	6.3E-03	1.00	1.00	1.00	1.00
1388323_at	NADH dehydrogenase (ubiquinone) 1 alpha subcomplex, 9	Ndufa9	362440	0.83	0.31	4.6E-01	1.1E-03	1.00	1.00	1.00	1.00
1388361_at	similar to NADH-ubiquinone oxidoreductase PDSW subunit (Complex I-PDSW) (CI-PDSW)	LOC681418	681418	0.73	0.31	3.1E-01	3.1E-03	1.00	1.00	1.00	1.00
1376750_at	Transcribed locus			0.83	0.31	8.4E-01	5.4E-03	0.00	0.00	0.00	0.50
1389159_at	Transcribed locus			0.86	0.31	7.0E-01	2.3E-03	1.00	0.88	0.00	1.00
1376465_at	Sim ilar to glucocorticoid induced gene 1 (predicted)	RGD1563869_predicted	310233	0.99	0.30	9.9E-01	1.7E-03	1.00	1.00	0.90	1.00
1376302_at	Coilin	Coil	50998	1.14	0.30	5.1E-01	1.2E-03	1.00	0.75	0.00	1.00
1389652_at	Similar to 2700029M09Rik protein (predicted)	RGD1311747_predicted	290706	0.82	0.30	4.8E-01	1.7E-03	1.00	1.00	1.00	1.00
1372684_at	Similar to ovary-specific MOB-like protein (predicted)	RGD1562983_predicted	499288	1.25	0.30	5.6E-01	4.0E-04	1.00	1.00	1.00	1.00
1373167_at	Transcribed locus			0.75	0.30	5.2E-01	8.8E-03	1.00	1.00	0.30	1.00
1373836_at	Forty-two-three domain containing 1	Fyttd1	360726	1.14	0.30	6.9E-01	3.7E-04	1.00	1.00	1.00	1.00
1372440_at	serine (or cysteine) proteinase inhibitor, clade E, member 2	Serpine2	29366	1.12	0.30	8.4E-01	4.5E-03	1.00	1.00	1.00	1.00
1373151_at	similar to RIKEN cDNA 2810489O06 (predicted)	RGD1560177_predicted	499615	0.97	0.30	9.3E-01	1.6E-04	1.00	1.00	1.00	1.00
1377462_at	Transcribed locus			1.24	0.30	7.7E-01	2.5E-02	0.20	0.00	0.00	0.50
1373883_at	Similar to PDZ-domain protein scribble (predicted)	RGD1565055_predicted	362938	0.89	0.30	5.2E-01	3.4E-03	1.00	1.00	0.20	1.00
1375545_at	RNA binding motif protein 9 (predicted)	Rbm9_predicted	362950	1.02	0.30	9.7E-01	1.7E-04	1.00	0.50	0.40	1.00
1368053_at	par-3 (partitioning defective 3) homolog (C. elegans)	Pard3	81918	0.91	0.30	8.2E-01	2.6E-03	1.00	0.88	0.00	1.00
1388870_at	similar to RNA-binding protein Musashi2-S (predicted)	RGD1560397_predicted	360596	0.82	0.30	3.2E-01	1.0E-03	1.00	1.00	1.00	1.00
1368298_at	adenylate cyclase 5	Adcy5	64532	0.99	0.30	9.7E-01	2.1E-03	1.00	1.00	0.40	1.00
1371475_at	ribonuclease, RNase A family 4	Rnase4	56759	1.36	0.30	1.4E-01	3.4E-02	1.00	1.00	1.00	1.00
1390126_at	Similar to protein tyrosine phosphatase-like (proline instead of catalytic arginine), member b	LOC690040	266708	0.93	0.30	8.2E-01	3.2E-03	1.00	1.00	0.50	1.00
1383176_at	similar to C11orf17 protein (predicted)	RGD1306959_predicted	361624	1.14	0.30	8.0E-01	1.2E-02	1.00	1.00	1.00	1.00
1373193_at	Hypothetical protein LOC690089	LOC690089	690089	0.71	0.30	4.9E-01	8.2E-03	1.00	1.00	1.00	1.00
1376770_at	similar to EF hand domain containing 1 (predicted)	RGD1559565_predicted	501181	0.59	0.30	1.0E-01	1.5E-02	1.00	1.00	0.00	1.00
1394234_x_at	Similar to IQ motif and WD repeats 1 (predicted)	RGD1561961_predicted	289181	0.80	0.30	6.7E-01	7.3E-04	0.80	1.00	1.00	1.00
1390711_at				1.03	0.30	9.8E-01	3.9E-02	0.60	0.50	0.00	1.00
1369960_at	GATA binding protein 4	Gata4	54254	0.69	0.30	2.9E-01	8.0E-03	1.00	1.00	1.00	1.00
1390255_at	similar to mKIAA0843 protein (predicted)	RGD1565118_predicted	307395	0.63	0.30	1.4E-01	1.4E-03	1.00	1.00	1.00	1.00
1371202_a_at	nuclear factor I/B	Nfib	29227	1.08	0.30	8.3E-01	2.2E-04	1.00	1.00	1.00	1.00
1370178_at	calcium channel, voltage-dependent, beta 2 subunit	Cacnb2	116600	0.83	0.30	3.3E-01	2.4E-03	1.00	1.00	1.00	1.00
1370816_at	nuclear receptor subfamily 1, group D, member 1	Nr1d1	252917	0.80	0.30	4.0E-01	8.3E-04	1.00	1.00	1.00	1.00
1389333_at	similar to F-box only protein 3 isoform 1	LOC687307	687307	1.11	0.30	7.0E-01	6.2E-04	1.00	1.00	1.00	1.00
1386993_at	myosin, heavy polypeptide 7, cardiac muscle, beta	Myh7	29557	1.93	0.30	5.2E-02	1.8E-02	1.00	1.00	1.00	1.00
1375295_at	Citrate synthase	Cs	170587	1.20	0.30	6.6E-01	4.3E-03	1.00	1.00	1.00	1.00
1388975_at	succinate-Coenzyme A ligase, ADP-forming, beta subunit (predicted)	Sucla2_predicted	361071	0.98	0.30	9.7E-01	1.1E-03	1.00	1.00	1.00	1.00
1375685_at	cytoplasmic linker associated protein 1	Clasp1	304740	0.93	0.30	8.9E-01	1.7E-04	1.00	1.00	1.00	1.00
1387709_at	c-fos induced growth factor	Figf	360457	0.61	0.30	7.3E-02	5.8E-02	0.00	0.00	0.00	0.63
1389332_at	Transcribed locus			1.82	0.30	8.4E-02	2.1E-04	1.00	1.00	1.00	1.00
1373937_at	FYVE and coiled-coil domain containing 1 (predicted)	Fyco1_predicted	301085	1.14	0.29	6.8E-01	1.9E-03	1.00	1.00	1.00	1.00
1388489_at	similar to NADH-ubiquinone oxidoreductase B9 subunit (Complex I-B9) (CI-B9)	LOC683547	683547	0.78	0.29	5.2E-01	9.9E-03	1.00	1.00	1.00	1.00
1389354_at	similar to semaF cytoplasmic domain associated protein 2	LOC365960	365960	0.69	0.29	2.5E-01	2.7E-03	1.00	1.00	1.00	1.00
1374173_at	Hexosaminidase B	Hexb	294673	0.79	0.29	7.1E-01	1.7E-02	0.80	1.00	0.10	1.00
1388925_at	sirtuin 5 (silent mating type information regulation 2 homolog) 5 (S. cerevisiae)	Sirt5	306840	0.92	0.29	7.9E-01	1.6E-03	1.00	1.00	1.00	1.00
1376572_a_at	supervillin (predicted)	Svil_predicted	361256	1.32	0.29	1.8E-01	4.1E-04	1.00	1.00	1.00	1.00
1371634_at	similar to RIKEN cDNA 1810020E01	RGD1305677	293113	0.82	0.29	4.9E-01	3.5E-03	1.00	1.00	1.00	1.00
1372338_at	similar to CG9240-PA	LOC687395	687395	1.06	0.29	9.0E-01	2.5E-03	1.00	1.00	1.00	1.00
1376125_at	Activity and neurotransmitter-induced early gene 11 (ania-11) mRNA, 3' UTR			1.10	0.29	7.0E-01	5.7E-04	1.00	1.00	1.00	1.00
1367828_at	acetyl-Coenzyme A dehydrogenase, short chain	Acads	64304	0.90	0.29	7.9E-01	2.5E-03	1.00	1.00	1.00	1.00
1388908_at	peroxisomal delta3, delta2-enoyl-Coenzyme A isomerase	Peci	291075	0.86	0.29	5.6E-01	1.4E-03	1.00	1.00	1.00	1.00
1388403_at	similar to NADP+-specific isocitrate dehydrogenase	LOC361596	361596	0.86	0.29	6.1E-01	4.4E-03	1.00	1.00	1.00	1.00
1372762_at	HD domain containing 3 (predicted)	Hddc3_predicted	308758	0.82	0.29	4.0E-01	9.1E-04	1.00	1.00	1.00	1.00

				Fold Change		False Discovery Rate		Average Present Call			
				Strain Effect	Rejection Effect						
Probe Set ID	Gene Title	Gene Symbol	Entrez Gene ID	Native BN / DA	Allograft / Isograft	Native BN / DA	Allograft / Isograft	Native BN	Native DA	Allograft	Isograft
1374275_at	carbonic anhydrase 11	Car11	308588	0.75	0.29	4.8E-01	8.9E-02	0.80	0.13	0.00	0.75
1377807_a_at	Similar to IQ motif and WD repeats 1 (predicted)	RGD1561961_predicted	289181	1.26	0.29	3.9E-01	8.7E-03	1.00	1.00	1.00	1.00
1375001_at	Transcribed locus			0.50	0.29	9.5E-02	1.2E-02	0.40	1.00	0.00	1.00
1388799_at	kelch-like 7 (Drosophila)	Klhl7	362303	1.43	0.29	6.4E-02	1.2E-03	1.00	1.00	1.00	1.00
1372733_at	similar to intracellular membrane-associated calcium-independent phospholipase A2 gamma (predicted)	RGD1311444_predicted	314075	0.99	0.29	9.9E-01	1.2E-03	1.00	1.00	1.00	1.00
1368008_at	prominin 1	Prom1	60357	0.52	0.29	9.8E-02	1.4E-03	1.00	1.00	1.00	1.00
1388569_at	serine (or cysteine) peptidase inhibitor, clade F, member 1	Serpinf1	287526	1.55	0.29	2.9E-01	7.6E-02	1.00	1.00	0.80	1.00
1375898_at	similar to RNA binding protein gene with multiple splicing (predicted)	RGD1561067_predicted	498642	1.06	0.29	8.2E-01	1.7E-03	1.00	1.00	1.00	1.00
1372603_at	protein kinase C and casein kinase substrate in neurons 3	Pacsin3	311187	1.29	0.29	3.7E-01	2.8E-03	1.00	1.00	1.00	1.00
1373892_at	F-box and leucine-rich repeat protein 17 (predicted)	Fbxl17_predicted	316663	0.89	0.29	6.7E-01	1.4E-03	1.00	1.00	1.00	1.00
1388546_at				1.18	0.29	6.0E-01	2.4E-03	1.00	1.00	1.00	1.00
1367907_a_at	clathrin, light polypeptide (Lcb)	Cltb	116561	1.05	0.29	8.8E-01	2.0E-03	1.00	1.00	1.00	1.00
1372273_at	glycophorin C (Gerbich blood group)	Gypc	364837	0.85	0.29	5.2E-01	3.6E-03	1.00	1.00	1.00	1.00
1374969_at	similar to phosphoglucomutase 5	LOC679990	679990	1.37	0.29	3.0E-01	1.4E-03	1.00	1.00	0.60	1.00
1376785_at	Synaptonemal complex protein 3	Sycp3	25561	0.88	0.29	8.0E-01	3.8E-02	1.00	1.00	0.20	1.00
1368371_at	potassium voltage-gated channel, subfamily Q, member 1	Kcnq1	84020	0.78	0.29	5.0E-01	5.0E-03	0.60	1.00	0.00	0.75
1374278_at	Transcribed locus			0.71	0.29	4.9E-02	3.2E-03	1.00	1.00	0.40	1.00
1377456_at				0.87	0.29	4.7E-01	1.7E-03	1.00	1.00	0.10	1.00
1389527_at	promethin	LOC378467	378467	0.51	0.29	2.3E-01	1.7E-03	1.00	1.00	1.00	1.00
1388343_at	NADH dehydrogenase (ubiquinone) 1 beta subcomplex, 7 (predicted)	Ndufb7_predicted	361385	0.64	0.29	2.3E-01	7.0E-03	1.00	1.00	1.00	1.00
1376200_at	Unknown (protein for MGC:72974)	MGC72974	316976	1.10	0.29	7.9E-01	1.2E-03	1.00	1.00	1.00	1.00
1374171_at	Transcribed locus			1.19	0.28	5.4E-01	1.5E-03	1.00	1.00	1.00	1.00
1369989_at	pyridoxine 5'-phosphate oxidase	Pnpo	64533	0.41	0.28	1.2E-01	7.0E-03	1.00	1.00	0.60	1.00
1388118_at	3-hydroxyisobutyrate dehydrogenase	Hibadh	63938	0.87	0.28	4.3E-01	1.6E-03	1.00	1.00	1.00	1.00
1379380_at	sprouty homolog 1 (Drosophila) (predicted)	Spry1_predicted	294981	1.04	0.28	9.4E-01	2.2E-04	1.00	1.00	0.00	1.00
1373351_at	ankyrin 2, neuronal	Ank2	362036	0.69	0.28	3.8E-01	1.9E-03	1.00	1.00	1.00	1.00
1390664_at	hypothetical protein LOC687361	LOC687361	687361	0.95	0.28	8.9E-01	2.6E-03	1.00	1.00	0.00	1.00
1388465_at	Transcribed locus			1.02	0.28	9.0E-01	3.3E-03	1.00	1.00	1.00	1.00
1386981_at	solute carrier family 16 (monocarboxylic acid transporters), member 1	Slc16a1	25027	1.45	0.28	2.6E-01	1.9E-04	1.00	1.00	1.00	1.00
1385548_at	similar to ubiquitin-conjugating enzyme E2D 1, UBC4/5 homolog	LOC687789	687789	0.82	0.28	2.8E-01	4.6E-04	1.00	1.00	0.00	1.00
1367897_at	acyl-Coenzyme A dehydrogenase, very long chain	Acadvl	25363	0.96	0.28	9.2E-01	2.6E-04	1.00	1.00	1.00	1.00
1376692_at	homeodomain interacting protein kinase 2 (predicted)	Hipk2_predicted	362342	1.17	0.28	6.8E-01	1.1E-03	1.00	0.50	0.20	1.00
1398417_at	feminization 1 homolog a (C. elegans)	Fem1a	316131	1.09	0.28	6.7E-01	1.0E-03	1.00	1.00	1.00	1.00
1398265_at	ATP-binding cassette, sub-family C (CFTR/MRP), member 9	Abcc9	25560	1.46	0.28	2.9E-01	1.7E-03	1.00	1.00	0.60	1.00
1377011_at	similar to hypothetical protein CG003 (predicted)	RGD1307034_predicted	304244	0.85	0.28	6.6E-01	4.8E-04	1.00	1.00	1.00	1.00
1370333_a_at	insulin-like growth factor 1	Igf1	24482	1.37	0.28	3.1E-01	1.2E-03	0.70	0.00	0.80	1.00
1389650_at	Transcribed locus, strongly similar to XP_214980.3 similar to hypothetical protein DKFZp547K1113 [Rattus norvegicus]			0.41	0.28	3.3E-03	1.0E-02	1.00	1.00	0.20	1.00
1387010_s_at	sodium channel, voltage-gated, type I, beta	Scn1b	29686	1.00	0.28	9.9E-01	4.4E-03	1.00	1.00	1.00	1.00
1390480_at	Transcribed locus			0.30	0.28	2.8E-01	5.4E-04	0.00	1.00	0.30	1.00
1374004_at	similar to RIKEN cDNA D030028O16	RGD1310143	298771	1.37	0.28	3.0E-01	2.9E-03	1.00	1.00	1.00	1.00
1370276_at	ATP synthase, H+ transporting, mitochondrial F1 complex, O subunit	Atp5o	192241	0.63	0.28	1.5E-01	4.8E-03	1.00	1.00	1.00	1.00
1377072_at	Transcribed locus, weakly similar to XP_238535.3 similar to ankyrin repeat domain 25 [Rattus norvegicus]			1.08	0.28	7.6E-01	6.1E-04	0.60	0.50	0.00	1.00
1372568_at	EPM2A (laforin) interacting protein 1 (predicted)	Epm2aip1_predicted	316021	1.11	0.28	5.9E-01	1.1E-03	1.00	1.00	1.00	1.00
1371482_at	NADH dehydrogenase (ubiquinone) Fe-S protein 2	Ndufs2	289218	0.90	0.28	6.5E-01	1.7E-03	1.00	1.00	1.00	1.00
1374060_at	Transcribed locus			1.09	0.28	8.6E-01	1.1E-03	1.00	1.00	1.00	1.00
1367678_at	succinate dehydrogenase complex, subunit A, flavoprotein (Fp)	Sdha	157074	1.01	0.28	9.8E-01	3.6E-03	1.00	1.00	1.00	1.00
1371554_at	similar to Telethonin (Titin cap protein)	LOC688173	688173	0.80	0.28	5.0E-01	1.2E-02	1.00	1.00	1.00	1.00
1383222_at	FERM-domain-containing protein 163SCII	LOC257646	257646	0.95	0.28	8.6E-01	1.7E-03	1.00	1.00	1.00	1.00
1374685_at	Transcribed locus			1.69	0.28	2.6E-01	3.0E-03	1.00	1.00	0.80	1.00
1374466_at	Transcribed locus			1.42	0.28	6.6E-01	1.2E-03	1.00	1.00	0.90	1.00
1390253_at	ankyrin repeat domain 9	Ankrd9	314457	1.13	0.28	7.3E-01	2.4E-02	0.40	0.13	0.00	0.50
1390203_at	Transcribed locus, strongly similar to XP_580018.1 hypothetical protein XP_580018 [Rattus norvegicus]			1.10	0.28	7.7E-01	3.3E-03	1.00	1.00	1.00	1.00
1369127_a_at	prostaglandin F receptor	Ptgfr	25652	0.89	0.28	8.1E-01	1.0E-02	0.50	0.13	0.00	1.00
1387398_at	protein kinase inhibitor, alpha	Pkia	114906	1.36	0.28	2.0E-01	8.1E-03	1.00	1.00	0.00	1.00
1372110_at	Transcribed locus			1.43	0.28	1.8E-01	1.2E-03	1.00	1.00	1.00	1.00
1382446_at	mitochondrial ATP synthase regulatory component factor B	Atp5s	362749	0.85	0.28	6.6E-01	4.0E-04	1.00	1.00	1.00	1.00
1390096_at	SH3-domain GRB2-like B1 (endophilin)	Sh3glb1	292156	0.81	0.28	6.2E-01	1.8E-03	1.00	1.00	1.00	1.00
1390592_at	similar to SH3-domain binding protein 3	LOC688018	688018	0.66	0.28	2.2E-01	2.3E-03	1.00	1.00	1.00	1.00
1373362_at	ubiquinol cytochrome c reductase core protein 2	Uqcrc2	293448	1.08	0.28	8.0E-01	2.0E-03	1.00	1.00	1.00	1.00
1370164_at	hydroxyacyl-Coenzyme A dehydrogenase/3-ketoacyl-Coenzyme A thiolase/enoyl-Coenzyme A hydratase (trifunctional protein), alpha subunit	Hadha	170670	0.89	0.28	7.0E-01	1.5E-03	1.00	1.00	1.00	1.00
1367818_at	coenzyme Q3 homolog, methyltransferase (yeast)	Coq3	29309	0.87	0.28	7.1E-01	8.1E-04	1.00	1.00	0.80	1.00
1372524_at	Transcribed locus			1.03	0.28	9.4E-01	2.2E-04	1.00	1.00	1.00	1.00
1367653_a_at	malate dehydrogenase 1, NAD (soluble)	Mdh1	24551	0.91	0.28	7.9E-01	1.2E-03	1.00	1.00	1.00	1.00
1389610_at	Transcribed locus			1.12	0.28	7.5E-01	2.7E-02	1.00	1.00	0.40	1.00
1386934_at	solute carrier family 6 (neurotransmitter transporter, creatine), member 8	Slc6a8	50690	1.79	0.27	1.3E-02	2.7E-03	1.00	1.00	1.00	1.00
1386867_at	brain protein 44-like	Brp44l	171087	0.91	0.27	7.2E-01	4.1E-03	1.00	1.00	1.00	1.00
1377463_at	Mannosidase 2, alpha 2 (predicted)	Man2a2_predicted	308757	0.93	0.27	9.1E-01	7.1E-03	1.00	0.75	0.80	1.00
1374959_at	NAD(P)H dehydrogenase, quinone 2	Nqo2	291084	0.63	0.27	1.3E-01	1.3E-03	1.00	1.00	1.00	1.00
1367869_at	oxidation resistance 1	Oxr1	117520	0.54	0.27	1.3E-01	2.2E-03	1.00	1.00	1.00	1.00
1371388_at	pyruvate dehydrogenase (lipoamide) beta	Pdhb	289950	0.89	0.27	6.2E-01	6.4E-04	1.00	1.00	1.00	1.00
1382061_at	lactate dehydrogenase D	Ldhd	307858	0.96	0.27	8.0E-01	1.1E-03	1.00	1.00	0.70	1.00
1390259_at	similar to ubiquitin-conjugating enzyme E2D 1, UBC4/5 homolog	LOC687789	687789	0.75	0.27	1.8E-01	4.3E-03	1.00	1.00	1.00	1.00
1373922_at	Transcribed locus			1.76	0.27	2.4E-01	2.9E-04	1.00	1.00	0.70	1.00
1371416_at	NADH dehydrogenase (ubiquinone) flavoprotein 1	Ndufv1	293655	0.95	0.27	7.9E-01	1.2E-03	1.00	1.00	1.00	1.00
1371369_at	procollagen, type VI, alpha 2	Col6a2	361821	1.36	0.27	3.7E-01	9.9E-02	0.50	0.25	0.80	1.00
1387059_at	serine/threonine kinase 39, STE20/SPS1 homolog (yeast)	Stk39	54348	0.77	0.27	4.9E-01	2.8E-03	1.00	1.00	1.00	1.00
1369679_a_at	nuclear factor I/A	Nfia	25492	1.25	0.27	5.1E-01	2.2E-03	1.00	1.00	0.60	1.00

				Fold Change		False Discovery Rate		Average Present Call			
				Strain Effect	Rejection Effect						
**Probe Set ID**	**Gene Title**	**Gene Symbol**	**Entrez Gene ID**	**Native BN / DA**	**Allograft / Isograft**	**Native BN / DA**	**Allograft / Isograft**	**Native BN**	**Native DA**	**Allograft**	**Isograft**
1373074_at	similar to RIKEN cDNA 2700002I20	RGD1307279	307210	0.83	0.27	5.4E-01	8.1E-04	1.00	1.00	1.00	1.00
1371323_at	General transcription factor IIIC, polypeptide 5 (predicted)	Gtf3c5_predicted	362095	0.85	0.27	6.6E-01	1.9E-03	1.00	1.00	1.00	1.00
1388160_a_at	isocitrate dehydrogenase 3 (NAD+) beta	Idh3B	311530	0.92	0.27	7.4E-01	2.1E-03	1.00	1.00	1.00	1.00
1374698_at	similar to Cytochrome c oxidase polypeptide VIIa-heart, mitochondrial precursor (Cytochrome c oxidase subunit VIIa-H) (COX VIIa-M)	LOC687508	687508	0.89	0.27	7.4E-01	3.2E-04	1.00	1.00	0.00	1.00
1389253_at	vanin 1	Vnn1	29142	1.57	0.27	3.8E-02	4.3E-04	1.00	1.00	1.00	1.00
1388304_at	NADH dehydrogenase (ubiquinone) 1 beta subcomplex, 5 (predicted)	Ndufb5_predicted	294964	0.81	0.27	4.1E-01	2.7E-03	1.00	1.00	1.00	1.00
1369633_at	chemokine (C-X-C motif) ligand 12	Cxcl12	24772	1.81	0.27	1.4E-02	9.1E-03	1.00	1.00	0.90	1.00
1376180_at	Similar to RIKEN cDNA 1700012B09 (predicted)	RGD1561795_predicted	500945	0.58	0.27	3.9E-01	2.6E-03	1.00	1.00	0.10	1.00
1373934_at				0.66	0.27	9.8E-02	1.3E-03	1.00	1.00	1.00	1.00
1376380_at	Transcribed locus			1.11	0.27	8.7E-01	5.6E-02	0.70	0.75	0.00	1.00
1371436_at	dimethylarginine dimethylaminohydrolase 2	Ddah2	294239	0.84	0.27	5.4E-01	3.0E-03	0.90	0.50	0.00	1.00
1374710_at	Transcribed locus			0.94	0.27	7.7E-01	7.5E-03	1.00	1.00	1.00	1.00
1388462_at	similar to SH3-binding kinase	LOC292603	292603	0.93	0.27	7.9E-01	2.8E-02	0.80	0.50	0.00	1.00
1392890_at	platelet-activating factor acetylhydrolase, isoform Ib, alpha subunit 45kDa	Pafah1b1	83572	1.21	0.27	8.6E-01	1.6E-02	0.40	0.25	0.00	0.88
1375517_at	tumor protein p53 inducible nuclear protein 2	Trp53inp2	362246	1.09	0.27	6.6E-01	3.7E-04	1.00	1.00	1.00	1.00
1384217_at	zinc fingers and homeoboxes 2	Zhx2	314988	1.06	0.27	9.5E-01	6.4E-04	1.00	1.00	1.00	1.00
1373223_at	Transcribed locus			0.77	0.26	5.2E-01	3.3E-03	0.00	0.00	0.20	1.00
1389601_at	nuclear factor I/B	Nfib	29227	0.89	0.26	6.6E-01	1.3E-03	1.00	1.00	0.90	1.00
1372159_at				0.81	0.26	4.0E-01	1.6E-03	1.00	1.00	1.00	1.00
1377015_at	Transcribed locus			0.75	0.26	3.3E-01	6.9E-04	1.00	1.00	0.20	1.00
1389180_at	Transcribed locus			0.89	0.26	7.8E-01	2.2E-03	1.00	1.00	0.60	1.00
1389024_at	similar to Sec1 family domain containing protein 2 (Syntaxin binding protein 1-like 1) (Neuronal Sec1)	LOC498353	498353	0.83	0.26	4.8E-01	1.2E-03	1.00	1.00	0.00	1.00
1373581_at	Similar to KIAA0672 gene product	RGD1305664	303222	1.62	0.26	6.4E-02	3.5E-04	1.00	1.00	0.30	1.00
1386974_at	pleckstrin homology-like domain, family B, member 1	Phldb1	171434	1.22	0.26	8.3E-01	4.4E-03	0.30	0.00	0.00	1.00
1387441_at	potassium channel, subfamily K, member 3	Kcnk3	29553	1.35	0.26	2.9E-01	4.0E-03	1.00	1.00	0.70	1.00
1387053_at	flavin containing monooxygenase 1	Fmo1	25256	1.13	0.26	8.0E-01	8.3E-04	1.00	1.00	0.10	1.00
1376184_at	Ly6/neurotoxin 1 (predicted)	Lynx1_predicted	300018	0.56	0.26	2.3E-01	9.5E-04	1.00	1.00	0.20	1.00
1388469_at	Insulin-like growth factor 1	Igf1	24482	1.52	0.26	3.7E-01	8.8E-03	1.00	1.00	1.00	1.00
1398378_at	glutathione S-transferase kappa 1	Gstk1	297029	0.82	0.26	4.3E-01	2.0E-03	1.00	1.00	1.00	1.00
1371576_at	mitochondrial ribosomal protein S36 (predicted)	Mrps36_predicted	294696	0.72	0.26	3.5E-01	2.1E-03	1.00	1.00	1.00	1.00
1387914_at	cytochrome P450, family 27, subfamily a, polypeptide 1	Cyp27a1	301517	0.77	0.26	5.3E-01	1.8E-03	1.00	1.00	1.00	1.00
1388611_at	transcription elongation factor A (SII), 3	Tcea3	298559	0.67	0.26	2.8E-01	3.1E-03	1.00	1.00	1.00	1.00
1399056_at	similar to CG10585-PA	LOC365592	365592	0.74	0.26	3.3E-01	4.0E-02	0.80	1.00	0.00	1.00
1398249_at	solute carrier family 25 (mitochondrial carnitine/acylcarnitine translocase), member 20	Slc25a20	117035	0.70	0.26	2.5E-01	3.0E-04	1.00	1.00	1.00	1.00
1390020_at	Similar to HORMA domain containing 2	LOC498400	305479	1.04	0.26	9.2E-01	1.2E-03	1.00	1.00	1.00	1.00
1370068_at	phospholipase A2, group V	Pla2g5	29354	0.76	0.26	3.8E-01	3.1E-04	1.00	1.00	0.00	1.00
1375916_at	protein-L-isoaspartate (D-aspartate) O-methyltransferase domain containing 2 (predicted)	Pcmtd2_predicted	311726	1.02	0.26	9.8E-01	4.6E-04	1.00	1.00	1.00	1.00
1368358_a_at	protein tyrosine phosphatase, receptor type, R	Ptprr	94202	1.47	0.26	2.3E-01	8.0E-03	1.00	1.00	1.00	1.00
1374179_at				1.00	0.26	9.9E-01	2.5E-03	1.00	1.00	1.00	1.00
1367572_at	myosin, light polypeptide 3	Myl3	24585	0.86	0.26	6.7E-01	3.8E-03	1.00	1.00	1.00	1.00
1374635_at	Hypothetical protein LOC689663	LOC689663	689663	1.42	0.26	3.5E-01	6.3E-02	0.20	0.13	0.00	0.50
1367680_at	acyl-Coenzyme A oxidase 1, palmitoyl	Acox1	50681	1.00	0.26	9.9E-01	1.3E-03	1.00	1.00	1.00	1.00
1370865_at	isocitrate dehydrogenase 3 (NAD), gamma	Idh3g	25179	0.75	0.26	2.9E-01	2.2E-03	1.00	1.00	1.00	1.00
1373564_at	3-hydroxyisobutyryl-Coenzyme A hydrolase	Hibch	301384	1.15	0.26	7.7E-01	9.1E-04	1.00	1.00	1.00	1.00
1371864_at	Transcribed locus			0.93	0.26	7.7E-01	4.3E-04	1.00	1.00	1.00	1.00
1388692_at	similar to spermatogenesis associated 11	LOC679944	679944	0.57	0.26	1.0E-01	2.8E-03	1.00	1.00	1.00	1.00
1368059_at	crystallin, mu	Crym	117024	0.70	0.26	2.8E-01	4.2E-04	1.00	1.00	1.00	1.00
1372109_at	Pre-B-cell leukemia transcription factor interacting protein 1	Pbxip1	310644	0.65	0.25	3.5E-01	4.1E-03	1.00	1.00	1.00	1.00
1376214_at	Transcribed locus			1.15	0.25	5.7E-01	1.9E-03	1.00	1.00	1.00	1.00
1372248_at	sestrin 1 (predicted)	Sesn1_predicted	294518	0.84	0.25	6.3E-01	2.5E-03	1.00	1.00	1.00	1.00
1368013_at	DNA-damage-inducible transcript 4-like	Ddit4l	140582	0.72	0.25	4.8E-01	1.2E-02	1.00	1.00	0.00	1.00
1377381_at	Transcribed locus			0.83	0.25	5.1E-01	2.0E-04	1.00	1.00	0.90	1.00
1388688_at	Transcribed locus			0.75	0.25	2.1E-01	1.7E-03	1.00	1.00	1.00	1.00
1374160_at	similar to RIKEN cDNA 1110063G11 (predicted)	RGD1311960_predicted	305095	0.49	0.25	4.9E-02	3.7E-04	1.00	1.00	1.00	1.00
1371736_at	hypothetical protein LOC679333	LOC679333	679333	0.65	0.25	2.3E-01	1.3E-03	1.00	1.00	1.00	1.00
1389964_at	TBC1 domain family, member 19 (predicted)	Tbc1d19_predicted	289657	0.80	0.25	5.4E-01	7.4E-03	1.00	1.00	1.00	1.00
1372507_at	T-cell leukemia translocation altered gene	Tcta	306587	0.79	0.25	5.9E-01	1.1E-03	1.00	1.00	0.80	1.00
1390852_x_at	Similar to IQ motif and WD repeats 1 (predicted)	RGD1561961_predicted	289181	1.36	0.25	4.2E-01	2.7E-03	1.00	1.00	1.00	1.00
1389015_at	similar to FUN14 domain containing 2 (predicted)	RGD1560916_predicted	361288	0.56	0.25	2.1E-02	9.8E-04	1.00	1.00	1.00	1.00
1372639_at	tripartite motif-containing 54	Trim54	362708	1.59	0.25	1.8E-01	6.0E-03	1.00	1.00	1.00	1.00
1371041_at	NADH dehydrogenase (ubiquinone) flavoprotein 2	Ndufv2	81728	0.85	0.25	5.6E-01	1.3E-03	1.00	1.00	1.00	1.00
1398343_at	similar to DnaJ (Hsp40) homolog, subfamily A, member 4	LOC498996	498996	0.89	0.25	5.8E-01	5.7E-04	1.00	1.00	1.00	1.00
1377773_at	Transcribed locus, weakly similar to NP_766020.1 cDNA C030002O17 [Mus musculus]			0.65	0.25	4.5E-01	4.6E-02	0.40	0.50	0.00	0.50
1374515_at	similar to RIKEN cDNA 6330409N04	RGD1306437	290303	0.82	0.25	6.0E-01	1.3E-03	1.00	1.00	1.00	1.00
1368618_at	growth factor receptor bound protein 14	Grb14	58844	1.16	0.25	6.3E-01	6.7E-04	1.00	1.00	1.00	1.00
1389966_at	procollagen, type VI, alpha 3 (predicted)	Col6a3_predicted	367313	1.10	0.25	8.0E-01	4.8E-02	1.00	1.00	1.00	1.00
1387039_at	glypican 1	Gpc1	58920	0.98	0.25	9.4E-01	3.3E-04	1.00	1.00	1.00	1.00
1389572_at	malic enzyme 3, NADP(+)-dependent, mitochondrial (predicted)	Me3_predicted	361602	1.03	0.25	9.3E-01	2.5E-03	1.00	1.00	0.80	1.00
1376194_at	Transcribed locus			1.21	0.25	7.1E-01	1.1E-03	1.00	0.88	1.00	1.00
1373186_at	similar to RIKEN cDNA 5033405K12 (predicted)	RGD1311593_predicted	305310	0.80	0.25	3.2E-01	1.1E-03	1.00	1.00	1.00	1.00
1374726_at	fibronectin type III domain containing 1	Fndc1	308099	1.07	0.25	9.2E-01	2.0E-02	0.00	0.25	0.20	1.00
1388447_at	similar to limb-bud and heart	LOC683626	683626	1.00	0.25	9.9E-01	3.9E-04	1.00	1.00	1.00	1.00
1388194_at	dihydrolipoamide S-acetyltransferase (E2 component of pyruvate dehydrogenase complex)	Dlat	81654	1.24	0.25	2.9E-01	3.2E-03	1.00	1.00	1.00	1.00
1373659_at	hypothetical protein LOC688257	LOC688257	688257	1.23	0.25	7.7E-01	5.7E-03	1.00	1.00	0.00	1.00
1373312_at	Transcribed locus			0.77	0.25	6.5E-01	1.1E-02	1.00	1.00	0.00	1.00
1372583_at	Transcribed locus			1.38	0.25	4.0E-01	8.1E-04	1.00	1.00	1.00	1.00
1368827_at	GATA binding protein 6	Gata6	29300	1.24	0.25	2.9E-01	2.0E-04	1.00	1.00	0.00	1.00

				Fold Change		False Discovery Rate		Average Present Call			
				Strain Effect	Rejection Effect						
Probe Set ID	Gene Title	Gene Symbol	Entrez Gene ID	Native BN / DA	Allograft / Isograft	Native BN / DA	Allograft / Isograft	Native BN	Native DA	Allograft	Isograft
1368064_a_at	dopa decarboxylase	Ddc	24311	1.17	0.25	6.2E-01	1.1E-03	1.00	1.00	0.60	1.00
1373790_at				0.87	0.25	7.6E-01	2.9E-03	1.00	1.00	0.00	1.00
1380854_at	R3H domain containing 1	R3hdm1	304763	0.99	0.24	9.9E-01	1.0E-03	1.00	1.00	1.00	1.00
1371995_at	kelch-like 21 (Drosophila) (predicted)	Klhl21_predicted	313743	1.07	0.24	8.6E-01	1.5E-02	1.00	0.88	0.00	1.00
1370541_at	nuclear receptor subfamily 1, group D, member 2	Nr1d2	259241	1.06	0.24	9.2E-01	8.6E-03	1.00	1.00	1.00	1.00
1388902_at	lysyl oxidase-like 1	Loxl1	315714	0.23	0.24	2.5E-01	2.4E-02	0.00	0.00	0.30	1.00
1377563_at	similar to leiomodin 3 (fetal) (predicted)	RGD1564924_predicted	500267	1.33	0.24	4.7E-01	8.8E-04	1.00	1.00	0.00	1.00
1372084_at	protein tyrosine phosphatase 4a3 (predicted)	Ptp4a3_predicted	362930	1.13	0.24	7.0E-01	1.1E-03	1.00	1.00	1.00	1.00
1374705_at	collagen, type IV, alpha 5 (predicted)	Col4a5_predicted	363457	0.97	0.24	9.7E-01	1.7E-04	1.00	1.00	1.00	1.00
1370312_at	spondin 1	Spon1	64456	2.40	0.24	5.2E-02	1.0E-02	1.00	1.00	1.00	1.00
1368132_at	transducer of ErbB-2.1	Tob1	170842	0.86	0.24	6.2E-01	1.3E-03	1.00	1.00	1.00	1.00
1376672_at	Transcribed locus			1.19	0.24	4.5E-01	5.8E-04	1.00	1.00	1.00	1.00
1373278_at	nuclear factor, erythroid derived 2,-like 1 (predicted)	Nfe2l1_predicted	360610	0.37	0.24	3.1E-03	5.2E-04	1.00	1.00	1.00	1.00
1372727_at	Transcribed locus			0.86	0.24	6.8E-01	5.3E-04	1.00	1.00	1.00	1.00
1374235_at	Down syndrome critical region gene 1-like 1	Dscr1l1	140666	0.87	0.24	7.2E-01	1.3E-03	1.00	1.00	0.10	1.00
1390430_at	Nuclear receptor subfamily 1, group D, member 2	Nr1d2	259241	0.75	0.24	3.3E-01	1.3E-03	1.00	1.00	1.00	1.00
1367694_at	hydroxyacyl-Coenzyme A dehydrogenase/3-ketoacyl-Coenzyme A thiolase/enoyl-Coenzyme A hydratase (trifunctional protein), beta subunit	Hadhb	171155	0.85	0.24	5.2E-01	9.3E-04	1.00	1.00	1.00	1.00
1377423_at	Transcribed locus			0.86	0.24	6.6E-01	1.9E-02	0.90	0.63	0.20	1.00
1370258_at	basic leucine zipper and W2 domains 2	Bzw2	171439	0.80	0.24	5.4E-01	2.2E-03	1.00	1.00	1.00	1.00
1373016_at	dihydrolipoamide dehydrogenase	Dld	298942	0.94	0.24	9.3E-01	1.0E-03	1.00	1.00	1.00	1.00
1374218_at	similar to IQ motif and Sec7 domain 2	LOC683475	683475	0.87	0.24	6.8E-01	1.4E-03	1.00	1.00	1.00	1.00
1368893_at	CAP, adenylate cyclase-associated protein, 2 (yeast)	Cap2	116653	1.03	0.24	9.3E-01	4.6E-03	1.00	1.00	0.60	1.00
1368114_at	fibroblast growth factor 13	Fgf13	84488	0.96	0.24	8.8E-01	8.8E-04	1.00	1.00	1.00	1.00
1387669_a_at	epoxide hydrolase 1, microsomal	Ephx1	25315	0.83	0.24	4.8E-01	3.7E-03	0.60	1.00	0.00	1.00
1387795_at	polymerase (DNA directed), alpha 2	Pola2	85242	1.39	0.24	3.6E-01	2.7E-02	1.00	1.00	0.00	0.75
1370389_at	glycoprotein m6b	Gpm6b	192179	1.40	0.24	4.9E-01	4.2E-02	0.60	0.50	0.20	1.00
1367687_a_at	peptidylglycine alpha-amidating monooxygenase	Pam	25508	0.95	0.24	8.2E-01	4.1E-03	1.00	1.00	1.00	1.00
1371414_at	gelsolin	Gsn	296654	1.14	0.24	7.1E-01	3.7E-04	1.00	1.00	1.00	1.00
1389521_at	Transcribed locus, strongly similar to NP_445868.1 perinuclear RNA-binding protein [Rattus norvegicus]	Mnab_predicted	311909	0.86	0.24	5.4E-01	4.9E-04	1.00	1.00	1.00	1.00
1373212_at	mitochondrial ATP synthase regulatory component factor B	Atp5s	362749	0.76	0.24	2.9E-01	2.4E-03	1.00	1.00	0.20	1.00
1389376_at	Transcribed locus			0.97	0.24	9.3E-01	3.7E-04	1.00	1.00	0.80	1.00
1369949_at	basal cell adhesion molecule	Bcam	78958	0.99	0.24	9.9E-01	8.0E-04	1.00	1.00	0.00	1.00
1373371_a_at	similar to RIKEN cDNA 1110001J03	MGC112899	500087	0.66	0.24	2.1E-01	1.5E-03	1.00	1.00	1.00	1.00
1375223_at				0.74	0.24	5.3E-01	2.4E-02	1.00	1.00	0.20	1.00
1372925_at	sirtuin 3 (silent mating type information regulation 2, homolog) 3 (S. cerevisiae) (predicted)	Sirt3_predicted	293615	0.87	0.23	3.5E-01	1.4E-03	1.00	1.00	0.60	1.00
1370814_at	dehydrogenase/reductase (SDR family) member 4	Dhrs4	266686	0.95	0.23	8.2E-01	3.7E-04	1.00	1.00	1.00	1.00
1372695_at	Fibronectin type III domain containing 5	Fndc5	260327	0.84	0.23	2.6E-01	2.0E-03	1.00	1.00	1.00	1.00
1371954_at	tensin	Tns	29136	1.07	0.23	8.3E-01	1.9E-03	1.00	1.00	1.00	1.00
1376498_at	similar to 2900002H16Rik protein (predicted)	RGD1307973_predicted	304469	0.76	0.23	5.3E-02	1.2E-03	1.00	1.00	1.00	1.00
1367813_at	protein phosphatase 1, regulatory (inhibitor) subunit 14A	Ppp1r14a	114004	1.07	0.23	8.5E-01	9.2E-02	0.20	0.00	0.10	1.00
1372044_at	similar to Ser/Thr-rich protein T10 in DGCR region (predicted)	RGD1310348_predicted	360738	0.82	0.23	6.2E-01	1.0E-03	1.00	1.00	1.00	1.00
1375297_at	similar to RIKEN cDNA 0610008C08 (predicted)	RGD1565289_predicted	363474	1.09	0.23	8.6E-01	6.3E-04	1.00	1.00	1.00	1.00
1370656_a_at	homer homolog 1 (Drosophila)	Homer1	29546	1.03	0.23	9.6E-01	3.5E-02	0.80	0.50	0.00	0.50
1372864_at	Transcribed locus			0.85	0.23	5.9E-01	5.0E-04	1.00	1.00	1.00	1.00
1375092_at	Transcribed locus, moderately similar to XP_620246.2 myosin XVIIIb [Mus musculus]			0.92	0.23	8.9E-01	7.4E-04	1.00	1.00	0.10	1.00
1398396_at	Transcribed locus			0.91	0.23	6.6E-01	1.3E-02	0.20	0.50	0.00	1.00
1372856_at	Transcribed locus			1.46	0.23	1.1E-01	1.6E-03	1.00	1.00	0.60	1.00
1368154_at	guanylate cyclase 1, soluble, alpha 3	Gucy1a3	497757	1.02	0.23	9.7E-01	4.0E-03	0.40	0.38	0.00	1.00
1389020_at	similar to immunoglobulin superfamily containing leucine-rich repeat	LOC686539	686539	0.64	0.23	2.8E-01	3.7E-04	1.00	1.00	1.00	1.00
1368574_at	adrenergic receptor, alpha 1b	Adra1b	24173	0.46	0.23	1.1E-01	2.7E-03	1.00	1.00	1.00	1.00
1390449_at	CDNA clone IMAGE:7309127			1.46	0.23	2.4E-01	1.2E-03	1.00	1.00	1.00	1.00
1372168_s_at	insulin-like growth factor binding protein 6	Igfbp6	25641	0.49	0.23	1.0E-01	2.4E-03	0.80	1.00	0.70	1.00
1389543_at	mannan-binding lectin serine peptidase 1	Masp1	64023	1.04	0.23	9.3E-01	6.7E-04	1.00	1.00	1.00	1.00
1376170_at				0.92	0.23	9.0E-01	2.0E-02	0.00	0.25	0.00	1.00
1371253_at	electron transferring flavoprotein, alpha polypeptide	Etfa	300726	0.84	0.23	4.8E-01	4.6E-04	1.00	1.00	1.00	1.00
1370342_at	potassium channel, subfamily K, member 2	Kcnk2	170899	0.37	0.23	5.3E-02	1.9E-03	1.00	1.00	0.00	1.00
1388145_at	tenascin XA	Tnxa	25602	1.51	0.23	3.1E-01	2.8E-03	1.00	1.00	0.30	1.00
1374039_at	Itchy homolog E3 ubiquitin protein ligase (mouse)	Itch	311567	0.77	0.23	5.0E-01	1.5E-03	1.00	1.00	0.00	1.00
1388804_at	Transcribed locus			1.37	0.23	4.8E-01	6.2E-02	0.70	0.50	0.00	1.00
1372199_at	PTEN induced putative kinase 1 (predicted)	Pink1_predicted	298575	0.94	0.23	7.6E-01	1.3E-03	1.00	1.00	1.00	1.00
1368429_at	TAF9-like RNA polymerase II, TATA box binding protein (TBP)-associated factor, 31kDa	Taf9l	171152	1.07	0.23	9.2E-01	1.9E-03	1.00	0.75	0.30	1.00
1372289_at	similar to solute carrier family 25 (mitochondrial carrier, Aralar), member 12 (predicted)	RGD1561141_predicted	311122	0.82	0.23	3.3E-01	3.7E-04	1.00	1.00	1.00	1.00
1390420_at	carboxypeptidase X 1 (M14 family) (predicted)	Cpxm1_predicted	296156	0.79	0.23	5.3E-01	2.0E-03	0.00	0.25	0.00	1.00
1372720_at	BTB (POZ) domain containing 1	Btbd1	293060	0.73	0.23	5.2E-02	5.5E-04	1.00	1.00	1.00	1.00
1377060_at	methylcrotonoyl-Coenzyme A carboxylase 2 (beta)	Mccc2	361884	1.01	0.23	9.6E-01	1.7E-03	1.00	1.00	1.00	1.00
1369674_at	purinergic receptor P2X, ligand-gated ion channel, 5	P2rx5	113995	0.99	0.23	9.9E-01	6.9E-04	1.00	1.00	1.00	1.00
1370165_at	small muscle protein, X-linked	Smpx	84416	0.92	0.23	8.4E-01	1.1E-02	1.00	1.00	1.00	1.00
1372979_at	Transcribed locus			1.06	0.23	8.0E-01	5.8E-03	1.00	1.00	1.00	1.00
1374352_at	actin alpha cardiac 1	Actc1	29275	0.94	0.22	8.2E-01	1.1E-02	1.00	1.00	1.00	1.00
1386914_at	guanosine monophosphate reductase	Gmpr	117533	0.95	0.22	9.0E-01	8.9E-04	1.00	1.00	1.00	1.00
1389386_at				0.82	0.22	5.5E-01	3.2E-04	1.00	1.00	0.60	1.00
1376912_at	hypothetical LOC287388 (predicted)	RGD1310324_predicted	287388	0.84	0.22	5.1E-01	3.5E-02	1.00	1.00	0.40	1.00
1379936_at	Tropomyosin 1, alpha	Tpm1	24851	1.26	0.22	6.6E-01	4.1E-02	1.00	0.88	0.20	1.00
1389704_at	Transcribed locus			1.26	0.22	2.0E-01	3.9E-04	1.00	1.00	0.00	1.00
1384309_at	Transcribed locus			1.00	0.22	1.0E+00	1.8E-03	1.00	1.00	0.20	1.00
1368772_at	solute carrier family 4, member 3	Slc4a3	24781	0.81	0.22	2.8E-01	5.2E-05	1.00	1.00	1.00	1.00
1368440_at	solute carrier family 3, member 1	Slc3a1	29484	0.16	0.22	1.7E-01	8.6E-04	0.00	1.00	0.60	1.00

				Fold Change		False Discovery Rate		Average Present Call			
				Strain Effect	Rejection Effect						
Probe Set ID	Gene Title	Gene Symbol	Entrez Gene ID	Native BN / DA	Allograft / Isograft	Native BN / DA	Allograft / Isograft	Native BN	Native DA	Allograft	Isograft
1377448_at	transmembrane protein 38a (predicted)	Tmem38a_predicted	306327	1.03	0.22	9.6E-01	3.5E-02	1.00	1.00	0.00	0.75
1388617_at	biphenyl hydrolase-like (serine hydrolase, breast epithelial mucin-associated antigen)	Bphl	361239	0.54	0.22	1.5E-01	7.3E-03	1.00	1.00	0.10	1.00
1368673_at	discoidin domain receptor family, member 2	Ddr2	83573	1.60	0.22	7.0E-01	2.5E-02	0.00	0.00	0.00	0.88
1386921_at	carboxypeptidase E	Cpe	25669	0.80	0.22	4.5E-01	7.3E-03	1.00	1.00	1.00	1.00
1370981_at	retinoid X receptor gamma	Rxrg	83574	0.94	0.22	8.6E-01	4.6E-04	1.00	1.00	0.20	1.00
1375549_at	Transcribed locus			0.71	0.22	4.8E-01	1.8E-03	1.00	1.00	0.00	1.00
1386880_at	acetyl-Coenzyme A acyltransferase 2 (mitochondrial 3-oxoacyl-Coenzyme A thiolase)	Acaa2	170465	0.81	0.22	7.0E-01	1.3E-03	1.00	1.00	1.00	1.00
1388305_at	v-raf murine sarcoma 3611 viral oncogene homolog	Araf	64363	0.71	0.22	5.8E-01	4.1E-04	1.00	1.00	1.00	1.00
1387370_at	tropomodulin 1	Tmod1	25566	1.51	0.22	2.1E-01	3.1E-03	1.00	1.00	0.50	1.00
1367913_at	cytoglobin	Cygb	170520	1.04	0.22	9.4E-01	2.1E-03	1.00	1.00	0.70	1.00
1388423_at	hypothetical protein MGC:15854	RGD1302996	294231	0.88	0.22	6.5E-01	2.8E-03	1.00	1.00	1.00	1.00
1368642_at	cadherin 2	Cdh2	83501	0.99	0.22	9.9E-01	1.7E-03	1.00	1.00	1.00	1.00
1384466_at	notch1-induced protein	LOC493574	493574	1.15	0.22	7.8E-01	4.8E-04	1.00	1.00	1.00	1.00
1387814_at	caveolin 3	Cav3	29161	1.04	0.22	9.3E-01	1.1E-02	1.00	1.00	0.50	1.00
1373178_at	Transcribed locus			1.28	0.22	4.9E-01	5.9E-04	1.00	1.00	0.50	1.00
1389334_at	NADH dehydrogenase (ubiquinone) 1 alpha subcomplex 10	Ndufa10	314071	0.58	0.22	6.9E-02	2.7E-03	1.00	1.00	1.00	1.00
1368317_at	aquaporin 7	Aqp7	29171	0.51	0.22	1.2E-01	9.9E-04	1.00	1.00	1.00	1.00
1377266_at	Transcribed locus			1.02	0.22	9.5E-01	2.6E-03	0.40	0.13	0.00	0.75
1388471_at	t-complex 11 (mouse) like 2	Tcp11l2	314683	1.35	0.22	2.4E-01	6.7E-04	1.00	1.00	1.00	1.00
1388364_at	NADH dehydrogenase (ubiquinone) Fe-S protein 3 (predicted)	Ndufs3_predicted	295923	0.75	0.22	3.6E-01	1.1E-03	1.00	1.00	1.00	1.00
1387018_at	Arg/Abl-interacting protein ArgBP2	Argbp2	114901	1.03	0.22	8.6E-01	7.2E-03	1.00	1.00	1.00	1.00
1389256_at	Transcribed locus			1.44	0.22	7.4E-02	4.0E-04	1.00	1.00	1.00	1.00
1370419_a_at	SH3-domain kinase binding protein 1	Sh3kbp1	84357	0.89	0.22	7.8E-01	3.7E-04	1.00	1.00	1.00	1.00
1377452_at	Transcribed locus			1.99	0.21	1.4E-01	6.1E-02	0.60	0.00	0.00	0.75
1368700_at	phospholipase C-like 1	Plcl1	84587	1.00	0.21	9.9E-01	2.9E-02	0.20	0.50	0.10	0.75
1389423_at	Discoidin domain receptor family, member 2	Ddr2	83573	1.22	0.21	5.9E-01	7.8E-03	1.00	1.00	1.00	1.00
1369926_at	glutathione peroxidase 3	Gpx3	64317	1.27	0.21	5.7E-01	8.6E-03	1.00	1.00	1.00	1.00
1375698_at	Transcribed locus			0.75	0.21	3.6E-01	6.2E-04	1.00	1.00	0.00	1.00
1390339_at	Transcribed locus			0.97	0.21	9.3E-01	9.3E-04	1.00	1.00	1.00	1.00
1372736_at	amylo-1, 6-glucosidase, 4-alpha-glucanotransferase	Agl_predicted	362029	1.12	0.21	7.1E-01	3.7E-04	1.00	1.00	1.00	1.00
1374062_x_at	microtubule-associated protein, RP/EB family, member 3	Mapre3	298848	0.87	0.21	5.7E-01	7.2E-04	1.00	1.00	0.40	1.00
1398354_at	catenin (cadherin associated protein), alpha-like 1 (predicted)	Ctnnal1_predicted	298019	0.71	0.21	2.3E-01	2.7E-03	1.00	1.00	0.80	1.00
1373810_at	phospholipase A2, group XIIA (predicted)	Pla2g12a_predicted	362039	0.46	0.21	5.9E-02	5.3E-02	1.00	1.00	0.00	0.75
1376441_at	similar to RIKEN cDNA 1810048J11 (predicted)	RGD1305147_predicted	314219	0.66	0.21	4.1E-01	1.2E-03	0.80	1.00	0.00	1.00
1367735_at	acetyl-Coenzyme A dehydrogenase, long-chain	Acadl	25287	0.90	0.21	6.6E-01	1.1E-03	1.00	1.00	1.00	1.00
1369876_at	guanine nucleotide binding protein, beta 3	Gnb3	60449	0.98	0.21	9.7E-01	1.4E-03	1.00	1.00	0.30	1.00
1372266_at	REV3-like, catalytic subunit of DNA polymerase zeta RAD54 like (S. cerevisiae)	Rev3l	309812	0.82	0.21	6.4E-01	1.0E-03	1.00	1.00	1.00	1.00
1390130_at	Transcribed locus			1.02	0.21	9.6E-01	4.9E-04	1.00	1.00	1.00	1.00
1373921_at	similar to enoyl Coenzyme A hydratase domain containing 3	LOC684538	684538	0.95	0.21	9.6E-01	2.3E-03	1.00	1.00	0.50	1.00
1369065_a_at	ATPase, Ca++ transporting, cardiac muscle, slow twitch 2	Atp2a2	29693	0.89	0.21	7.1E-01	4.1E-03	1.00	1.00	1.00	1.00
1371412_a_at	Neuronal regeneration related protein	Nrep	338475	0.68	0.21	4.4E-01	6.5E-04	1.00	1.00	1.00	1.00
1370678_s_at	monoamine oxidase A	Maoa	29253	1.28	0.21	5.8E-01	1.2E-03	1.00	1.00	1.00	1.00
1374353_x_at	actin alpha cardiac 1	Actc1	29275	1.04	0.21	9.0E-01	1.0E-02	1.00	1.00	1.00	1.00
1388133_at	cold shock domain containing C2, RNA binding	Csdc2	266600	0.79	0.21	3.8E-01	7.5E-04	1.00	1.00	0.00	1.00
1387197_at	osteomodulin	Omd	83717	0.87	0.21	7.4E-01	1.7E-03	0.80	1.00	0.20	1.00
1370301_at	matrix metallopeptidase 2	Mmp2	81686	1.23	0.21	6.0E-01	4.4E-02	1.00	1.00	1.00	1.00
1377808_at	Similar to IQ motif and WD repeats 1 (predicted)	RGD1561961_predicted	289181	1.38	0.21	4.1E-01	5.9E-03	1.00	1.00	1.00	1.00
1389179_at	cell death-inducing DNA fragmentation factor, alpha subunit-like effector A (predicted)	Cidea_predicted	291541	0.82	0.21	5.8E-01	4.8E-04	1.00	1.00	1.00	1.00
1370018_at	heat shock 27kDa protein 2	Hspb2	161476	0.85	0.21	4.8E-01	2.2E-02	1.00	1.00	0.90	1.00
1370328_at	dickkopf homolog 3 (Xenopus laevis)	Dkk3	171548	1.30	0.20	5.2E-01	1.8E-03	1.00	1.00	0.20	1.00
1398326_at	similar to Nur77 downstream protein 2	MGC105647	361824	0.84	0.20	5.6E-01	2.5E-03	1.00	1.00	1.00	1.00
1374389_at	guanylate cyclase 1, soluble, beta 3	Gucy1b3	25202	0.76	0.20	5.4E-01	6.8E-02	0.50	0.25	0.20	0.88
1371886_at	carnitine acetyltransferase	Crat	311849	0.63	0.20	8.4E-02	3.4E-03	1.00	1.00	1.00	1.00
1376015_at				1.14	0.20	5.9E-01	2.0E-02	1.00	1.00	0.20	1.00
1371609_at	homolog of zebrafish ES1	RGD1303003	294326	0.86	0.20	5.1E-01	1.6E-03	1.00	1.00	1.00	1.00
1387957_a_at	SH3-domain kinase binding protein 1	Sh3kbp1	84357	1.02	0.20	9.7E-01	1.2E-03	1.00	1.00	1.00	1.00
1388782_at	transcription factor 21	Tcf21	252856	1.19	0.20	7.0E-01	2.7E-02	1.00	1.00	0.20	1.00
1374454_at	protein-L-isoaspartate (D-aspartate) O-methyltransferase domain containing 2 (predicted)	Pcmtd2_predicted	311726	1.12	0.20	7.9E-01	2.3E-04	1.00	1.00	1.00	1.00
1368098_a_at	small nuclear ribonucleoprotein N	Snrpn	81781	0.72	0.20	3.6E-01	3.7E-03	1.00	1.00	0.00	1.00
1370930_at	Active BCR-related gene (predicted)	Abr_predicted	287537	0.99	0.20	9.6E-01	9.9E-04	1.00	1.00	1.00	1.00
1368894_at	CAP, adenylate cyclase-associated protein, 2 (yeast)	Cap2	116653	1.07	0.20	8.4E-01	1.1E-03	1.00	1.00	0.60	1.00
1369625_at	aquaporin 1	Aqp1	25240	0.95	0.20	9.1E-01	1.1E-03	1.00	1.00	1.00	1.00
1376537_at	Protein tyrosine phosphatase, non-receptor type 3	Ptpn3	362524	0.38	0.20	1.2E-02	5.3E-04	0.80	1.00	0.40	1.00
1373684_at	Transcribed locus			1.22	0.20	4.2E-01	2.3E-03	1.00	1.00	0.80	1.00
1388697_at	inositol polyphosphate-5-phosphatase A (predicted)	Inpp5a_predicted	365382	0.91	0.20	7.0E-01	7.9E-04	1.00	1.00	1.00	1.00
1398248_s_at	myosin, heavy polypeptide 6, cardiac muscle, alpha	Myh6	29556	1.13	0.20	6.7E-01	1.4E-02	1.00	1.00	1.00	1.00
1386951_at	NADH dehydrogenase (ubiquinone) 1 alpha subcomplex 5	Ndufa5	25488	0.59	0.20	2.9E-01	1.1E-03	1.00	1.00	1.00	1.00
1371963_at	similar to Propionyl-CoA carboxylase alpha chain, mitochondrial precursor	LOC687008	687008	0.84	0.20	4.7E-01	8.2E-04	1.00	1.00	0.80	1.00
1371358_at	glycoprotein, synaptic 2	Gpsn2	191576	1.10	0.20	7.2E-01	1.6E-03	1.00	1.00	1.00	1.00
1389757_at	Metadherin	Mtdh	170910	0.77	0.20	4.4E-01	2.7E-02	0.70	1.00	0.00	1.00
1370550_at	limbic system-associated membrane protein	Lsamp	29561	0.74	0.20	3.4E-01	6.9E-03	0.70	1.00	0.00	0.75
1370209_at	Kruppel-like factor 9	Klf9	117560	0.78	0.20	4.4E-01	3.9E-04	1.00	1.00	1.00	1.00
1388583_at	chemokine (C-X-C motif) ligand 12	Cxcl12	24772	1.14	0.20	5.9E-01	1.7E-03	1.00	1.00	1.00	1.00
1371577_at	NADH dehydrogenase (ubiquinone) Fe-S protein 1	Ndufs1	301458	1.04	0.20	7.9E-01	2.8E-04	1.00	1.00	1.00	1.00
1371483_at	nicotinamide nucleotide transhydrogenase (mapped)	Nnt	310378	0.78	0.20	2.5E-01	1.2E-03	1.00	1.00	1.00	1.00
1387401_at	calsequestrin 2	Casq2	29209	0.93	0.20	7.6E-01	5.8E-04	1.00	1.00	1.00	1.00
1371625_at	brain glycogen phosphorylase	Pygb	25739	0.94	0.19	9.0E-01	4.0E-04	1.00	1.00	1.00	1.00

				Fold Change Strain Effect	Fold Change Rejection Effect	False Discovery Rate		Average Present Call			
Probe Set ID	Gene Title	Gene Symbol	Entrez Gene ID	Native BN / DA	Allograft / Isograft	Native BN / DA	Allograft / Isograft	Native BN	Native DA	Allograft	Isograft
1388718_at	tropomodulin 1	Tmod1	25566	1.23	0.19	4.0E-01	4.5E-03	1.00	1.00	1.00	1.00
1368272_at	glutamate oxaloacetate transaminase 1	Got1	24401	1.16	0.19	6.8E-01	4.8E-04	1.00	1.00	1.00	1.00
1389772_at	Transcribed locus			0.86	0.19	3.6E-01	2.8E-02	1.00	1.00	0.00	1.00
1368322_at	superoxide dismutase 3, extracellular	Sod3	25352	1.15	0.19	6.0E-01	4.8E-04	1.00	1.00	0.30	1.00
1376222_at	similar to HCDI protein (predicted)	RGD1309307_predicted	361044	0.83	0.19	5.8E-01	1.3E-03	1.00	1.00	0.00	0.88
1388358_at	electron-transfer-flavoprotein, beta polypeptide	Etfb	292845	0.88	0.19	7.0E-01	6.2E-04	1.00	1.00	1.00	1.00
1367589_at	aconitase 2, mitochondrial	Aco2	79250	0.97	0.19	9.4E-01	4.5E-04	1.00	1.00	1.00	1.00
1370390_at	coronin, actin binding protein 6	Coro6	245982	1.14	0.19	5.4E-01	1.2E-03	1.00	1.00	1.00	1.00
1369085_s_at	small nuclear ribonucleoprotein N	Snrpn	113938	1.03	0.19	9.6E-01	7.9E-04	1.00	1.00	1.00	1.00
1367777_at	2,4-dienoyl CoA reductase 1, mitochondrial	Decr1	117543	0.83	0.19	4.8E-01	1.0E-03	1.00	1.00	1.00	1.00
1373604_at	hypothetical protein LOC680199	LOC680199	680199	0.79	0.19	2.3E-01	1.0E-02	1.00	1.00	0.00	1.00
1370237_at	L-3-hydroxyacyl-Coenzyme A dehydrogenase, short chain	Hadhsc	113965	1.01	0.19	9.7E-01	5.6E-04	1.00	1.00	1.00	1.00
1367763_at	acetyl-coenzyme A acetyltransferase 1	Acat1	25014	1.01	0.19	9.8E-01	3.7E-04	1.00	1.00	1.00	1.00
1376927_at	similar to LOC432779 protein (predicted)	RGD1564978_predicted	502225	1.02	0.19	9.6E-01	1.0E-03	1.00	1.00	1.00	1.00
1388604_at	similar to Calsequestrin-1 precursor (Calsequestrin, skeletal muscle isoform)	LOC679341	679341	0.89	0.19	7.8E-01	3.3E-04	1.00	1.00	1.00	1.00
1388961_at	integrin beta 1 binding protein 2 (predicted)	Itgb1bp2_predicted	317258	0.90	0.19	6.6E-01	2.4E-03	1.00	1.00	0.60	1.00
1371389_at	hypothetical LOC306766	LOC306766	306766	0.43	0.19	4.4E-02	7.6E-04	1.00	1.00	1.00	1.00
1373911_at	periostin, osteoblast specific factor (predicted)	Postn_predicted	361945	0.63	0.19	7.3E-01	9.2E-02	0.00	0.00	1.00	1.00
1374088_at	myopalladin (predicted)	Mypn_predicted	309760	1.03	0.19	9.4E-01	1.7E-02	1.00	1.00	0.60	1.00
1371942_at	similar to Glutathione S-transferase, theta 3 (predicted)	RGD1562732_predicted	499422	0.90	0.19	6.6E-01	5.3E-03	0.70	1.00	0.00	1.00
1372641_at	Alpha-kinase 3 (predicted)	Alpk3_predicted	365298	1.00	0.19	9.9E-01	5.1E-04	1.00	1.00	1.00	1.00
1375349_at	similar to sorbin and SH3 domain containing 1 isoform 3	LOC678826	678826	1.11	0.19	7.6E-01	3.1E-03	1.00	1.00	1.00	1.00
1372103_at	similar to DnaJ (Hsp40) homolog, subfamily A, member 4	LOC498996	498996	0.86	0.19	4.2E-01	3.8E-04	1.00	1.00	1.00	1.00
1375476_at	muscle glycogen phosphorylase	Pygm	24701	1.52	0.19	2.4E-01	3.8E-04	1.00	1.00	0.00	1.00
1368038_at	synaptojanin 2 binding protein	Synj2bp	64531	0.78	0.19	3.8E-01	7.4E-04	1.00	1.00	1.00	1.00
1372240_at	sarcoglycan, alpha (dystrophin-associated glycoprotein) (predicted)	Sgca_predicted	303468	0.85	0.19	5.5E-01	6.7E-04	1.00	1.00	1.00	1.00
1368566_a_at	NADH dehydrogenase (ubiquinone) flavoprotein 3-like	Ndufv3l	64539	0.74	0.19	3.7E-01	3.1E-03	1.00	1.00	1.00	1.00
1376852_at	methylcrotonoyl-Coenzyme A carboxylase 1 (alpha)	Mccc1	294972	1.13	0.19	7.9E-01	3.1E-03	1.00	1.00	0.80	1.00
1368716_at	protein phosphatase 1, regulatory (inhibitor) subunit 14c	Ppp1r14c	171010	1.05	0.19	9.3E-01	3.2E-03	1.00	1.00	0.00	1.00
1370956_at	decorin	Dcn	29139	1.18	0.19	5.8E-01	2.5E-02	1.00	1.00	1.00	1.00
1371421_at	similar to 3-oxoacid CoA transferase 1	LOC678860	678860	1.12	0.18	6.0E-01	3.0E-03	1.00	1.00	1.00	1.00
1377256_at	Transcribed locus			0.09	0.18	2.3E-02	7.9E-04	0.00	0.38	0.00	1.00
1389411_at	Transcribed locus			1.53	0.18	8.4E-02	4.5E-03	1.00	1.00	0.60	1.00
1374017_at	Similar to tripartite motif protein 50 (predicted)	RGD1562778_predicted	365377	1.76	0.18	4.9E-02	8.8E-04	1.00	1.00	1.00	1.00
1367829_at	enoyl Coenzyme A hydratase, short chain, 1, mitochondrial	Echs1	140547	1.14	0.18	6.4E-01	1.2E-03	1.00	1.00	0.60	1.00
1376495_at	Transcribed locus			1.19	0.18	7.5E-01	2.4E-03	1.00	1.00	0.00	1.00
1371794_at	similar to SH3-domain binding protein 3	LOC688018	688018	1.01	0.18	9.8E-01	4.8E-04	1.00	1.00	1.00	1.00
1370854_at	nexilin	Nexn	246172	1.09	0.18	6.6E-01	4.1E-03	1.00	1.00	1.00	1.00
1371519_at	electron-transferring-flavoprotein dehydrogenase	Etfdh	295143	1.10	0.18	7.6E-01	3.4E-04	1.00	1.00	1.00	1.00
1369948_at	nerve growth factor receptor (TNFRSF16) associated protein 1	Ngfrap1	117089	1.06	0.18	9.1E-01	4.1E-03	1.00	1.00	0.40	1.00
1387893_at	complement component 1, s subcomponent	C1s	192262	1.70	0.18	2.3E-01	7.0E-03	1.00	1.00	1.00	1.00
1370697_a_at	nexilin	Nexn	246172	1.28	0.18	1.9E-01	7.6E-03	1.00	1.00	1.00	1.00
1372157_at	bolA-like 1 (E. coli) (predicted)	Bola1_predicted	365875	0.61	0.18	1.5E-01	1.4E-03	1.00	1.00	0.50	1.00
1389460_at	Transcribed locus			1.61	0.18	2.2E-01	5.5E-04	1.00	1.00	0.40	1.00
1373188_at	sodium channel, voltage-gated, type IV, beta	Scn4b	315611	0.79	0.18	1.2E-01	1.2E-03	1.00	1.00	1.00	1.00
1372158_at	LRP16 protein	Lrp16	246233	0.81	0.18	5.8E-01	1.3E-03	1.00	1.00	0.30	1.00
1373674_at	microfibrillar associated protein 5 (predicted)	Mfap5_predicted	362429	0.58	0.18	2.9E-01	1.8E-02	0.20	1.00	0.80	1.00
1375518_at	titin	Ttn	84015	1.17	0.18	5.2E-01	1.7E-03	1.00	1.00	1.00	1.00
1388211_s_at	cytosolic acyl-CoA thioesterase 1	Cte1	192272	1.06	0.18	8.9E-01	6.3E-02	0.90	0.50	0.00	1.00
1374496_at	similar to RIKEN cDNA 1200009O22; EST AI316813	RGD1310827	362364	0.81	0.18	5.6E-01	5.5E-04	1.00	1.00	0.30	1.00
1389774_at	Transcribed locus			0.78	0.18	1.8E-01	1.8E-02	0.60	1.00	0.30	1.00
1388570_at	similar to RIKEN cDNA 2310005O14	LOC498909	498909	0.91	0.18	7.4E-01	6.7E-04	1.00	1.00	1.00	1.00
1390033_at				1.18	0.18	7.5E-01	1.1E-02	0.80	0.25	0.00	0.75
1372848_at	Transcribed locus			0.94	0.18	8.6E-01	1.0E-03	1.00	1.00	0.00	1.00
1370036_at	sulfite oxidase	Suox	81805	1.03	0.18	9.5E-01	6.0E-03	1.00	1.00	0.00	1.00
1367799_at	eukaryotic translation elongation factor 1 alpha 2	Eef1a2	24799	1.11	0.18	7.3E-01	1.5E-04	1.00	1.00	1.00	1.00
1376128_at	Transcribed locus			1.49	0.18	2.9E-01	4.7E-03	1.00	1.00	0.00	1.00
1373953_at	solute carrier family 4 (anion exchanger), member 1, adaptor protein (predicted)	Slc4a1ap_predicted	298805	1.01	0.18	9.8E-01	1.6E-03	1.00	1.00	1.00	1.00
1388916_at	Similar to novel protein (predicted)	RGD1564074_predicted	500514	1.04	0.17	9.2E-01	1.3E-03	1.00	1.00	1.00	1.00
1385704_at	tensin	Tns	29136	1.69	0.17	3.3E-01	2.4E-03	1.00	0.63	0.00	1.00
1372475_at	PTEN induced putative kinase 1 (predicted)	Pink1_predicted	298575	0.92	0.17	8.1E-01	1.2E-03	1.00	1.00	1.00	1.00
1368988_at	calsequestrin 2	Casq2	29209	0.84	0.17	5.7E-01	8.6E-04	1.00	1.00	1.00	1.00
1374307_at	Transcribed locus			0.93	0.17	8.6E-01	6.4E-03	1.00	1.00	0.40	1.00
1389449_at	similar to CG10671-like (predicted)	RGD1306911_predicted	290223	0.79	0.17	5.2E-01	1.4E-04	1.00	1.00	0.80	1.00
1374796_at	similar to Beta-sarcoglycan (Beta-SG) (43 kDa dystrophin-associated glycoprotein) (43DAG)	LOC687025	687025	0.81	0.17	4.5E-01	9.9E-04	1.00	1.00	1.00	1.00
1390227_at	hypothetical protein LOC678810	LOC678810	678810	0.49	0.17	7.6E-02	5.0E-02	0.80	1.00	0.00	1.00
1369698_at	ATP-binding cassette, sub-family C (CFTR/MRP), member 3	Abcc3	140668	0.92	0.17	7.9E-01	4.1E-03	0.50	0.75	0.00	0.50
1372400_at	similar to cullin 4A (predicted)	RGD1563853_predicted	361181	0.98	0.17	9.8E-01	4.0E-04	1.00	1.00	1.00	1.00
1392633_at	Transcribed locus			0.85	0.17	7.5E-01	4.8E-04	1.00	1.00	0.00	1.00
1367749_at	lumican	Lum	81682	0.84	0.17	6.2E-01	5.1E-02	1.00	1.00	1.00	1.00
1376175_at	similar to NipSnap2 protein (Glioblastoma amplified sequence)	LOC498174	498174	0.74	0.17	6.5E-01	1.9E-04	1.00	1.00	1.00	1.00
1367885_at	peroxisomal membrane protein 2	Pxmp2	29533	1.00	0.17	1.0E+00	2.5E-03	1.00	1.00	1.00	1.00
1373011_at	hypothetical protein LOC619558	LOC619558	619558	1.01	0.17	9.8E-01	1.0E-03	1.00	1.00	1.00	1.00
1379747_at	Protease, serine, 35	Prss35	315866	0.77	0.17	9.0E-01	2.6E-03	0.00	0.00	0.20	1.00
1370232_at	isovaleryl coenzyme A dehydrogenase	Ivd	24513	1.16	0.17	5.1E-01	1.9E-03	1.00	1.00	1.00	1.00
1373920_at	Similar to CG10084-PA	RGD1308297	306137	0.71	0.17	2.8E-01	2.9E-03	1.00	1.00	1.00	1.00
1368924_at	growth hormone receptor	Ghr	25235	1.77	0.17	3.0E-01	1.5E-03	1.00	1.00	0.60	1.00

				Fold Change		False Discovery Rate		Average Present Call			
				Strain Effect	Rejection Effect						
Probe Set ID	Gene Title	Gene Symbol	Entrez Gene ID	Native BN / DA	Allograft / Isograft	Native BN / DA	Allograft / Isograft	Native BN	Native DA	Allograft	Isograft
1370648_a_at	SH3 domain binding protein CR16	Cr16	259242	0.80	0.17	5.3E-01	6.5E-03	1.00	1.00	0.60	1.00
1376619_at	similar to protein tyrosine phosphatase, receptor type, D (predicted)	RGD1561090_predicted	313278	1.70	0.17	7.3E-01	4.6E-04	0.60	0.25	0.00	1.00
1387373_at	phosphodiesterase 4D interacting protein (myomegalin)	Pde4dip	64183	1.44	0.17	3.5E-01	3.0E-04	1.00	1.00	1.00	1.00
1372595_at	actinin alpha 2 (predicted)	Actn2_predicted	291245	0.96	0.17	9.1E-01	4.5E-03	1.00	1.00	1.00	1.00
1370259_a_at	parathyroid hormone receptor 1	Pthr1	56813	1.07	0.17	9.0E-01	4.9E-02	0.00	0.25	0.00	1.00
1372423_at	PERP, TP53 apoptosis effector (predicted)	Perp_predicted	292949	1.21	0.17	7.6E-01	9.9E-04	1.00	1.00	0.30	1.00
1374648_at	Transcribed locus			0.41	0.17	8.4E-02	6.9E-04	0.80	1.00	0.00	1.00
1389694_at	Transcribed locus			0.82	0.17	8.0E-01	1.8E-02	1.00	1.00	0.00	1.00
1372457_at	mitochondrial tumor suppressor 1	Mtus1	306487	0.93	0.16	7.2E-01	2.0E-04	1.00	1.00	1.00	1.00
1375849_at	RGM domain family, member A (predicted)	Rgma_predicted	308739	0.76	0.16	6.6E-01	3.3E-03	1.00	1.00	0.20	1.00
1367702_at	acetyl-Coenzyme A dehydrogenase, medium chain	Acadm	24158	0.99	0.16	9.9E-01	9.5E-04	1.00	1.00	1.00	1.00
1370952_at	glutathione S-transferase, mu 2	Gstm2	24424	1.44	0.16	4.5E-01	4.3E-04	1.00	1.00	1.00	1.00
1372452_at	Transcribed locus			0.54	0.16	1.7E-01	4.8E-04	1.00	1.00	0.80	1.00
1388456_at	S100 calcium binding protein A1	S100a1	295214	1.10	0.16	8.4E-01	1.2E-02	1.00	1.00	0.00	1.00
1375182_at	Solute carrier family 3, member 1	Slc3a1	29484	1.29	0.16	5.1E-01	3.4E-04	1.00	1.00	1.00	1.00
1373082_at	Transcribed locus			0.72	0.16	1.1E-01	1.6E-03	1.00	1.00	1.00	1.00
1377133_at	Hypothetical protein LOC680687	LOC680687	680687	0.14	0.16	1.3E-01	4.0E-03	0.00	1.00	0.00	1.00
1387625_at	insulin-like growth factor binding protein 6	Igfbp6	25641	0.38	0.16	6.9E-02	1.9E-03	0.00	1.00	0.20	1.00
1374935_at	Transcribed locus			1.18	0.16	5.7E-01	4.8E-04	1.00	1.00	1.00	1.00
1370019_at	sulfotransferase family 1A, phenol-preferring, member 1	Sult1a1	83783	0.59	0.16	3.6E-01	6.0E-04	1.00	1.00	0.60	1.00
1374335_at	GATA binding protein 6	Gata6	29300	0.88	0.16	8.1E-01	4.4E-05	1.00	1.00	1.00	1.00
1370061_at	RAB3B, member RAS oncogene family	Rab3b	81755	0.94	0.16	8.6E-01	2.4E-03	1.00	1.00	0.00	1.00
1373915_at	dystrophia myotonica-protein kinase (predicted)	Dmpk_predicted	308405	0.85	0.16	6.2E-01	6.2E-04	1.00	1.00	1.00	1.00
1386965_at	lipoprotein lipase	Lpl	24539	1.15	0.16	4.0E-01	1.0E-03	1.00	1.00	1.00	1.00
1367659_s_at	dodecenoyl-coenzyme A delta isomerase	Dci	29740	0.70	0.16	2.1E-01	6.1E-04	1.00	1.00	1.00	1.00
1383169_at	Transcribed locus			1.33	0.16	4.6E-01	1.3E-03	1.00	1.00	1.00	1.00
1367739_at	Cytochrom c oxidase subunit VIII-H (heart/muscle)	Cox8h	25250	0.80	0.16	5.4E-01	1.1E-02	1.00	1.00	1.00	1.00
1369957_at	regulator of G-protein signaling 5	Rgs5	54294	1.23	0.15	6.3E-01	1.9E-03	1.00	1.00	0.60	1.00
1372820_at	Transcribed locus			1.03	0.15	9.4E-01	5.2E-02	1.00	1.00	0.40	1.00
1375638_at	serum deprivation response protein	Sdpr	316384	1.07	0.15	9.0E-01	3.2E-04	1.00	1.00	1.00	1.00
1384178_at	leucine rich repeat containing 4B (predicted)	Lrrc4b_predicted	308571	0.72	0.15	3.8E-01	2.0E-04	1.00	1.00	1.00	1.00
1388300_at	microsomal glutathione S-transferase 3 (predicted)	Mgst3_predicted	289197	0.98	0.15	9.8E-01	7.3E-04	1.00	1.00	1.00	1.00
1373427_at	Ras-related GTP binding D (predicted)	Rragd_predicted	297960	0.60	0.15	1.3E-01	8.1E-03	1.00	1.00	0.00	1.00
1374172_at	Transcribed locus			1.54	0.15	2.9E-01	5.5E-04	0.60	0.00	0.20	1.00
1390766_at	similar to Tubulin alpha-8 chain (Alpha-tubulin 8)	LOC500377	500377	0.88	0.15	4.8E-01	2.7E-03	1.00	1.00	0.70	1.00
1372646_at	similar to RIKEN cDNA 1500015O10 (predicted)	RGD1305645_predicted	363225	0.33	0.15	2.9E-01	3.7E-03	0.00	0.00	0.00	0.75
1374989_at	ankyrin repeat and SOCS box-containing protein 12	Asb12	503446	0.78	0.15	4.0E-01	1.4E-02	1.00	1.00	0.00	1.00
1376575_at	Transcribed locus			0.74	0.15	4.9E-01	4.5E-04	1.00	1.00	0.80	1.00
1376105_at	procollagen, type XIV, alpha 1 (predicted)	Col14a1_predicted	314981	1.09	0.15	8.6E-01	7.6E-03	1.00	1.00	0.50	1.00
1367892_at	pyruvate dehydrogenase kinase, isoenzyme 2	Pdk2	81530	0.92	0.15	8.1E-01	2.7E-04	1.00	1.00	0.60	1.00
1374029_at				0.78	0.15	3.1E-01	2.5E-02	1.00	1.00	0.00	1.00
1388497_at	thioesterase superfamily member 2 (predicted)	Them2_predicted	291135	0.65	0.15	1.6E-01	7.7E-04	1.00	1.00	1.00	1.00
1392939_at	solute carrier family 41, member 3	Slc41a3	641603	1.34	0.15	3.8E-01	4.1E-04	1.00	1.00	1.00	1.00
1389583_at	similar to Hypothetical protein MGC37938 (predicted)	RGD1566169_predicted	298605	0.73	0.15	2.7E-01	2.2E-03	1.00	1.00	0.20	1.00
1383058_at	Transcribed locus			0.95	0.15	8.7E-01	2.8E-04	1.00	1.00	1.00	1.00
1387004_at	neuroblastoma, suppression of tumorigenicity 1	Nbl1	50594	0.79	0.15	5.4E-01	6.0E-03	0.10	0.00	0.00	1.00
1370973_at	sodium channel, voltage-gated, type VII, alpha	Scn7a	64155	1.07	0.15	9.0E-01	2.5E-02	1.00	1.00	0.50	1.00
1388506_at	desmoplakin	Dsp	306871	1.46	0.15	1.2E-01	5.5E-04	1.00	1.00	1.00	1.00
1370906_at	branched chain keto acid dehydrogenase E1, beta polypeptide	Bckdhb	29711	1.04	0.15	9.4E-01	1.0E-03	1.00	1.00	0.00	1.00
1372390_at	Transcribed locus			0.96	0.14	9.7E-01	3.7E-04	1.00	1.00	0.00	1.00
1377275_at	ADP-ribosyltransferase 1 (predicted)	Art1_predicted	308873	2.64	0.14	6.4E-02	2.5E-02	1.00	0.50	0.00	0.50
1389171_at	transmembrane protein 38a (predicted)	Tmem38a_predicted	306327	0.94	0.14	7.7E-01	1.2E-03	1.00	1.00	1.00	1.00
1371677_at	similar to Esophagus cancer-related gene-2 protein precursor (ECRG-2) (predicted)	RGD1307150_predicted	301016	0.58	0.14	1.3E-01	1.7E-03	1.00	1.00	1.00	1.00
1371356_at	tensin like C1 domain containing phosphatase (predicted)	Tenc1_predicted	315326	0.94	0.14	9.4E-01	1.6E-02	1.00	0.50	0.00	1.00
1373803_a_at	growth hormone receptor	Ghr	25235	1.18	0.14	6.4E-01	3.2E-04	1.00	1.00	0.40	1.00
1386927_at	carnitine palmitoyltransferase 2	Cpt2	25413	0.98	0.14	9.7E-01	7.3E-04	1.00	1.00	0.00	1.00
1386931_at	troponin I type 3 (cardiac)	Tnni3	29248	0.84	0.14	7.0E-01	6.7E-03	1.00	1.00	1.00	1.00
1372297_at	glutathione S-transferase, alpha 4	Gsta4	300850	0.53	0.14	1.1E-01	7.9E-04	1.00	1.00	1.00	1.00
1386962_at	phospholipase C, beta 4	Plcb4	25031	1.02	0.14	9.6E-01	2.9E-04	1.00	1.00	1.00	1.00
1370229_at	N-myc downstream regulated gene 4	Ndrg4	64457	1.23	0.14	3.4E-01	3.7E-04	1.00	1.00	1.00	1.00
1374038_at	Transcribed locus			1.11	0.14	8.5E-01	2.1E-04	1.00	1.00	1.00	1.00
1374444_at	plexin B1 (predicted)	Plxnb1_predicted	316009	2.10	0.14	4.9E-02	7.8E-03	1.00	1.00	0.00	1.00
1371951_at	Transcribed locus			1.00	0.14	9.9E-01	1.3E-03	1.00	1.00	1.00	1.00
1387344_at	aldehyde dehydrogenase family 6, subfamily A1	Aldh6a1	81708	1.23	0.14	6.2E-01	4.6E-04	1.00	1.00	1.00	1.00
1388432_at	optineurin	Optn	246294	1.29	0.14	4.3E-01	7.0E-04	1.00	1.00	0.70	1.00
1371672_at	chromobox homolog 7	Cbx7	362962	0.91	0.14	9.2E-01	3.0E-03	0.80	0.38	0.00	0.50
1370157_at	Similar to RIKEN cDNA 2600005C20 (predicted)	RGD1305633_predicted	309673	0.89	0.14	5.5E-01	3.2E-04	1.00	1.00	1.00	1.00
1367673_at	selenium binding protein 2	Selenbp1	140927	1.04	0.14	9.5E-01	1.5E-04	1.00	1.00	0.00	1.00
1388422_at	LIM and senescent cell antigen like domains 2	Lims2	361303	1.28	0.14	5.8E-01	4.5E-03	1.00	1.00	0.80	1.00
1374056_at	similar to RNA binding motif protein 24	LOC681858	681858	1.03	0.13	9.5E-01	1.4E-03	1.00	1.00	1.00	1.00
1374166_at	Transcribed locus			0.90	0.13	7.6E-01	4.9E-03	1.00	1.00	0.60	1.00
1369968_at	pleiotrophin	Ptn	24924	1.59	0.13	3.1E-01	3.3E-02	1.00	0.88	0.60	1.00
1372613_at	3-hydroxybutyrate dehydrogenase, type 2 (predicted)	Bdh2_predicted	295458	0.61	0.13	2.8E-01	1.8E-02	1.00	1.00	0.00	1.00
1372536_at	chaperone, ABC1 activity of bc1 complex like (S. pombe)	Cabc1	360887	0.69	0.13	2.1E-01	1.2E-03	1.00	1.00	1.00	1.00
1387703_a_at	ubiquitin specific peptidase 2	Usp2	115771	0.85	0.13	6.2E-01	9.6E-04	1.00	1.00	0.80	1.00
1377271_at	unc-45 homolog B (C. elegans) (predicted)	Unc45b_predicted	303373	1.19	0.13	5.8E-01	1.4E-04	1.00	1.00	0.20	1.00
1367864_at	phosphofructokinase, muscle	Pfkm	65152	0.90	0.13	6.2E-01	1.2E-04	1.00	1.00	1.00	1.00

Probe Set ID	Gene Title	Gene Symbol	Entrez Gene ID	Fold Change – Strain Effect: Native BN / DA	Fold Change – Rejection Effect: Allograft / Isograft	False Discovery Rate: Native BN / DA	False Discovery Rate: Allograft / Isograft	Average Present Call: Native BN	Average Present Call: Native DA	Average Present Call: Allograft	Average Present Call: Isograft
1372527_at	reticulon 2 (Z-band associated protein)	Rtn2	308410	0.72	0.13	2.4E-01	6.8E-04	1.00	1.00	0.00	1.00
1370897_at	branched chain ketoacid dehydrogenase E1, alpha polypeptide	Bckdha	25244	0.95	0.13	8.9E-01	5.2E-04	1.00	1.00	0.40	1.00
1376789_at	Similar to Myosin light chain kinase 2, skeletal/cardiac muscle (MLCK2) (predicted)	RGD1305801_predicted	291926	0.76	0.13	2.5E-01	1.0E-03	1.00	1.00	1.00	1.00
1388527_at	Transcribed locus			0.84	0.13	8.8E-01	1.0E-03	0.60	0.63	0.40	1.00
1371500_at	latent transforming growth factor beta binding protein 4	Ltbp4	292734	1.06	0.13	8.8E-01	4.1E-03	1.00	1.00	0.80	1.00
1373947_at	dermatopontin (predicted)	Dpt_predicted	289178	2.04	0.13	7.2E-02	9.5E-04	1.00	1.00	1.00	1.00
1390404_at	laminin, alpha 2 (predicted)	Lama2_predicted	309368	0.83	0.13	5.0E-01	2.9E-04	1.00	1.00	0.20	1.00
1380883_at	Transcribed locus, strongly similar to XP_001061572.1 hypothetical protein [Rattus norvegicus]			1.40	0.13	4.5E-01	3.8E-03	1.00	0.50	0.00	1.00
1388597_at	myosin binding protein C, cardiac (predicted)	Mybpc3_predicted	295929	1.31	0.13	2.7E-01	9.1E-04	1.00	1.00	1.00	1.00
1376892_at	CDNA clone IMAGE:7313785			0.88	0.13	7.7E-01	1.7E-03	1.00	1.00	0.60	1.00
1376590_at	Transcribed locus			0.81	0.13	5.5E-01	7.4E-04	1.00	1.00	0.00	1.00
1387047_at	heat shock 27kDa protein 3	Hspb3	78951	0.80	0.13	4.9E-01	5.8E-04	1.00	1.00	0.30	1.00
1383188_at	Transcribed locus			1.13	0.12	7.1E-01	8.7E-03	1.00	1.00	0.00	1.00
1384202_at	similar to Tescalcin (predicted)	RGD1566317_predicted	288689	0.82	0.12	6.3E-01	3.5E-03	1.00	1.00	1.00	1.00
1385924_at	Transcribed locus			0.37	0.12	4.7E-01	2.8E-02	0.00	0.88	0.00	0.63
1376905_at	Transcribed locus			0.70	0.12	8.3E-01	6.5E-03	0.00	0.00	0.00	0.75
1386885_at	enoyl coenzyme A hydratase 1, peroxisomal	Ech1	64526	0.72	0.12	3.6E-01	1.8E-04	1.00	1.00	1.00	1.00
1389618_at	Transcribed locus			1.14	0.12	7.8E-01	1.4E-04	1.00	1.00	0.60	1.00
1371566_at	similar to F-box protein FBL2 (predicted)	RGD1311830_predicted	363083	1.11	0.12	8.2E-01	1.5E-02	1.00	1.00	0.60	1.00
1387804_at	tripartite motif protein 63	Trim63	140939	1.04	0.12	8.9E-01	4.8E-04	1.00	1.00	0.60	1.00
1370161_at	steroid sensitive gene 1	Ssg1	64387	1.38	0.12	5.0E-01	3.5E-03	1.00	1.00	1.00	1.00
1388689_at	similar to Acylphosphatase, muscle type isozyme (Acylphosphate phosphohydrolase)	LOC682245	682245	1.13	0.12	8.5E-01	4.7E-04	1.00	1.00	1.00	1.00
1390112_at	epidermal growth factor-containing fibulin-like extracellular matrix protein 1	Efemp1	305604	1.62	0.12	2.8E-01	2.5E-02	1.00	0.75	0.20	1.00
1388866_at	Transcribed locus			0.71	0.12	2.1E-01	2.8E-04	1.00	1.00	1.00	1.00
1388349_at				0.97	0.12	9.3E-01	3.6E-03	1.00	1.00	1.00	1.00
1387121_a_at	N-myc downstream regulated gene 2	Ndrg2	171114	1.09	0.12	6.8E-01	1.6E-03	1.00	1.00	1.00	1.00
1374667_at	CDNA clone IMAGE:7302289			0.99	0.12	9.9E-01	6.3E-04	1.00	1.00	0.60	1.00
1370941_at	platelet derived growth factor receptor, alpha polypeptide	Pdgfra	25267	1.12	0.12	8.3E-01	6.2E-02	1.00	1.00	0.40	1.00
1373130_at	Transcribed locus			1.23	0.12	4.9E-01	2.2E-03	1.00	1.00	1.00	1.00
1371933_at	Glycine-, glutamate-, thienylcyclohexylpiperidine-binding protein	LOC246295	246295	1.09	0.12	7.5E-01	4.2E-04	1.00	1.00	1.00	1.00
1389727_at	leucine-rich repeat-containing 10 (predicted)	Lrrc10_predicted	314848	1.28	0.11	2.0E-01	7.2E-04	1.00	1.00	1.00	1.00
1383072_at	muscle glycogen phosphorylase	Pygm	24701	1.61	0.11	2.5E-01	2.0E-04	1.00	1.00	1.00	1.00
1398445_at	Transcribed locus			3.64	0.11	1.6E-01	1.9E-02	1.00	0.25	0.00	0.88
1387022_at	aldehyde dehydrogenase family 1, member A1	Aldh1a1	24188	0.70	0.11	2.5E-01	5.6E-04	1.00	1.00	0.20	1.00
1374311_at	similar to tripartite motif-containing 56	LOC691246	691246	0.96	0.11	9.3E-01	2.5E-02	1.00	1.00	0.20	1.00
1373648_at	similar to Protein C6orf142 homolog	LOC681849	681849	0.75	0.11	2.4E-01	9.9E-04	1.00	1.00	1.00	1.00
1372383_at	G-protein signalling modulator 1 (AGS3-like, C. elegans)	Gpsm1	246254	0.97	0.11	9.4E-01	3.7E-04	1.00	1.00	1.00	1.00
1375990_a_at	Transcribed locus			0.91	0.11	7.3E-01	3.7E-04	1.00	1.00	1.00	1.00
1374099_at	nebulin-related anchoring protein (predicted)	Nrap_predicted	307982	1.63	0.11	1.8E-01	1.5E-03	1.00	1.00	1.00	1.00
1372790_at	Malate dehydrogenase 1, NAD (soluble)	Mdh1	24551	0.86	0.11	5.8E-01	1.9E-04	1.00	1.00	1.00	1.00
1372162_at	acyl-CoA synthetase short-chain family member 1 (predicted)	Acss1_predicted	296259	1.22	0.11	6.5E-01	2.7E-03	1.00	1.00	0.70	1.00
1367742_at	carnitine palmitoyltransferase 1b, muscle	Cpt1b	25756	0.96	0.11	9.5E-01	2.1E-04	1.00	1.00	1.00	1.00
1375031_at	Transcribed locus, moderately similar to XP_342453.2 titin [Rattus norvegicus]			1.08	0.11	8.1E-01	4.6E-04	1.00	1.00	0.70	1.00
1387271_at	phytanoyl-CoA hydroxylase	Phyh	114209	0.69	0.10	2.9E-01	4.9E-04	1.00	1.00	1.00	1.00
1369313_at	four and a half LIM domains 2	Fhl2	63839	1.16	0.10	6.2E-01	2.9E-04	1.00	1.00	1.00	1.00
1369126_at	prostaglandin F receptor	Ptgfr	25652	0.50	0.10	6.5E-01	2.6E-02	0.00	0.25	0.00	0.50
1370982_at	muscle glycogen phosphorylase	Pygm	24701	1.15	0.10	5.9E-01	8.1E-04	1.00	1.00	0.60	1.00
1383060_at	G kinase anchoring protein 1	Gkap1	361202	1.22	0.10	6.2E-01	9.2E-03	0.40	0.00	0.00	0.75
1374148_at	Microtubule-associated protein tau	Mapt	29477	0.61	0.10	1.4E-01	4.8E-04	0.60	1.00	0.00	0.63
1387071_a_at	microtubule-associated protein tau	Mapt	29477	1.11	0.10	8.0E-01	1.9E-02	0.90	0.88	0.20	1.00
1374190_at	citrate lyase beta like	Clybl	306198	0.93	0.10	8.2E-01	8.9E-03	1.00	1.00	0.00	1.00
1373882_at				0.96	0.10	9.5E-01	1.6E-02	0.10	0.25	0.40	1.00
1388153_at	acyl-CoA synthetase long-chain family member 1	Acsl1	25288	0.87	0.10	6.6E-01	3.9E-04	1.00	1.00	0.80	1.00
1386911_at	ATPase, Na+/K+ transporting, alpha 2 polypeptide	Atp1a2	24212	0.81	0.10	3.6E-01	5.6E-04	1.00	1.00	1.00	1.00
1390365_at	leukocyte receptor cluster (LRC) member 1 (predicted)	Leng1_predicted	292535	0.64	0.10	4.8E-01	2.9E-04	0.20	0.50	0.00	0.75
1374816_at	similar to hypothetical protein FLJ30973	LOC363091	363091	1.07	0.10	8.4E-01	1.1E-04	1.00	1.00	1.00	1.00
1368249_at	Kruppel-like factor 15	Klf15	85497	0.87	0.10	6.3E-01	1.1E-03	1.00	1.00	0.50	1.00
1388876_at	Transcribed locus			0.83	0.10	5.0E-01	1.0E-03	1.00	1.00	1.00	1.00
1368542_at	zinc finger protein 423	Zfp423	94188	0.96	0.09	9.4E-01	1.9E-02	0.90	1.00	0.00	1.00
1374616_at	platelet-derived growth factor receptor-like	Pdgfrl	290771	0.79	0.09	8.4E-01	4.0E-02	0.00	0.00	0.00	0.63
1374895_at	myomesin 1 (skelemin) 185kDa	Myom1	316740	1.09	0.09	8.7E-01	1.2E-03	1.00	1.00	1.00	1.00
1367626_at	creatine kinase, muscle	Ckm	24265	1.00	0.09	9.9E-01	4.1E-04	1.00	1.00	1.00	1.00
1386907_at	Carboxypeptidase D	Cpd	25306	1.29	0.09	6.2E-01	9.3E-04	1.00	1.00	1.00	1.00
1367880_at	laminin, beta 2	Lamb2	25473	1.06	0.09	8.8E-01	3.5E-04	1.00	1.00	1.00	1.00
1371801_at	myozenin 2 (predicted)	Myoz2_predicted	295426	0.87	0.09	6.1E-01	1.1E-03	1.00	1.00	1.00	1.00
1367660_at	fatty acid binding protein 3	Fabp3	79131	0.68	0.09	2.5E-01	1.0E-03	1.00	1.00	1.00	1.00
1386961_at	phosphofructokinase, muscle	Pfkm	65152	1.42	0.09	3.9E-01	1.6E-04	1.00	1.00	0.00	1.00
1389532_at	Nebulette (predicted)	Nebl_predicted	307189	1.32	0.09	3.7E-01	1.1E-03	1.00	1.00	0.00	1.00
1370739_x_at	triadin	Trdn	59299	1.26	0.09	3.7E-01	6.5E-04	1.00	1.00	0.80	1.00
1391428_at	Transcribed locus			0.52	0.09	3.4E-01	1.7E-05	1.00	1.00	0.70	1.00
1389488_at				1.06	0.09	9.0E-01	2.7E-04	1.00	1.00	1.00	1.00
1389548_at	alcohol dehydrogenase, iron containing, 1	Adhfe1	362474	0.86	0.09	6.6E-01	3.8E-03	1.00	1.00	0.00	0.75
1388712_at	pleckstrin homology, Sec7 and coiled-coil domains 3	Pscd3	116693	0.71	0.09	8.5E-01	2.4E-03	0.40	0.25	0.00	1.00
1387023_at	glutathione S-transferase, mu type 3	Gstm3	81869	0.82	0.09	6.7E-01	1.8E-03	1.00	1.00	0.20	1.00
1369210_at	sodium channel, voltage-gated, type I, alpha	Scn1a	81574	1.10	0.09	8.5E-01	9.1E-03	1.00	0.50	0.00	1.00
1370939_at	acyl-CoA synthetase long-chain family member 1	Acsl1	25288	0.88	0.09	5.9E-01	2.0E-04	1.00	1.00	1.00	1.00
1371746_at	Transcribed locus			0.77	0.09	2.7E-01	8.7E-04	1.00	1.00	1.00	1.00

				Fold Change		False Discovery Rate		Average Present Call			
				Strain Effect	Rejection Effect						
Probe Set ID	Gene Title	Gene Symbol	Entrez Gene ID	Native BN / DA	Allograft / Isograft	Native BN / DA	Allograft / Isograft	Native BN	Native DA	Allograft	Isograft
1367989_at	solute carrier family 2 (facilitated glucose transporter), member 4	Slc2a4	25139	0.85	0.09	5.1E-01	8.8E-04	1.00	1.00	1.00	1.00
1376716_at	Transcribed locus			0.64	0.09	3.0E-01	8.1E-04	0.40	0.75	0.00	1.00
1387138_at	tachykinin 2	Tac2	29191	0.36	0.09	1.9E-01	9.2E-03	0.00	0.00	0.00	0.75
1372565_at	HtrA serine peptidase 3 (predicted)	Htra3_predicted	360959	1.27	0.09	5.4E-01	1.9E-03	1.00	1.00	0.20	1.00
1382954_at	Corin	Corin	289596	1.59	0.09	2.1E-01	3.0E-04	1.00	1.00	0.60	1.00
1367949_at	proenkephalin 1	Penk1	29237	0.61	0.09	2.9E-01	1.5E-03	1.00	1.00	0.60	1.00
1390716_at	Transcribed locus			0.85	0.09	7.2E-01	5.2E-04	1.00	1.00	0.20	1.00
1388802_at	brain expressed X-linked 1	Bex1	501625	2.00	0.09	2.2E-01	6.4E-04	1.00	1.00	0.10	1.00
1369326_at	A kinase (PRKA) anchor protein 6	Akap6	64553	1.63	0.08	4.8E-01	9.4E-04	1.00	0.00	0.00	1.00
1389095_at	biregional cell adhesion molecule-related/down-regulated by oncogenes (Cdon) binding protein (predicted)	Boc_predicted	360715	0.80	0.08	8.5E-01	7.8E-04	0.20	0.00	0.00	1.00
1381504_at	similar to asporin precursor	LOC306805	306805	0.78	0.08	5.4E-01	1.7E-02	0.40	0.25	0.10	1.00
1398458_at	similar to protein kinase, lysine deficient 1; kinase deficient protein (predicted)	RGD1307284_predicted	306811	1.05	0.08	9.0E-01	3.0E-03	1.00	1.00	0.00	1.00
1386943_at	plasma membrane proteolipid	Pllp	64364	0.38	0.08	9.8E-02	5.8E-04	0.80	1.00	0.00	1.00
1374217_at	similar to chromosome 16 open reading frame 5	RGD1310686	360480	0.77	0.08	6.5E-01	1.3E-03	1.00	1.00	0.00	1.00
1379884_at	Aspartate-beta-hydroxylase (predicted)	Asph_predicted	312981	0.94	0.08	8.7E-01	4.4E-04	1.00	1.00	0.80	1.00
1389066_at	Down syndrome critical region gene 1-like 1	Dscr1l1	140666	0.93	0.08	8.0E-01	7.4E-04	1.00	1.00	1.00	1.00
1388778_at	Transcribed locus			0.66	0.08	1.2E-01	2.0E-03	1.00	1.00	1.00	1.00
1373755_at	similar to leucine rich repeat containing 39 isoform 2	LOC686192	686192	0.83	0.08	5.5E-01	7.0E-04	1.00	1.00	1.00	1.00
1370310_at	3-hydroxy-3-methylglutaryl-Coenzyme A synthase 2	Hmgcs2	24450	0.73	0.08	7.9E-01	1.1E-04	0.00	0.00	0.00	0.75
1373696_at	Bicaudal C homolog 1 (Drosophila) (predicted)	Bicc1_predicted	361832	0.73	0.08	8.4E-02	3.6E-04	1.00	1.00	0.00	1.00
1389362_at	protein tyrosine phosphatase, non-receptor type 3	Ptpn3	362524	0.68	0.08	1.4E-01	3.6E-04	1.00	1.00	1.00	1.00
1386865_at	SPARC-like 1 (mast9, hevin)	Sparcl1	25434	1.60	0.08	1.5E-01	1.9E-04	1.00	1.00	1.00	1.00
1372372_at	similar to Ab2-225	RGD1306952	310201	0.60	0.07	1.8E-01	4.8E-04	1.00	1.00	0.60	1.00
1376746_at	lactate dehydrogenase D	Ldhd	307858	0.84	0.07	7.9E-01	1.8E-03	1.00	1.00	0.00	1.00
1372615_at	amine oxidase, copper containing 3	Aoc3	29473	1.28	0.07	5.2E-01	6.5E-04	1.00	1.00	0.00	1.00
1374677_at	similar to Adenylosuccinate synthetase isozyme 1	LOC684425	684425	0.76	0.07	4.7E-01	2.1E-04	1.00	1.00	1.00	1.00
1370738_a_at	triadin	Trdn	59299	0.78	0.07	7.2E-01	7.0E-03	1.00	1.00	0.00	1.00
1389908_at	Transcribed locus			1.43	0.07	6.3E-01	3.4E-03	0.80	0.38	0.00	1.00
1369654_at	protein kinase, AMP-activated, alpha 2 catalytic subunit	Prkaa2	78975	1.74	0.07	6.2E-02	3.6E-02	1.00	1.00	0.20	1.00
1371472_at	Transcribed locus			0.96	0.07	8.8E-01	1.8E-04	1.00	1.00	1.00	1.00
1389398_at	ankyrin 1, erythroid	Ank1	306570	0.85	0.07	5.6E-01	1.3E-04	1.00	1.00	0.10	1.00
1373778_at	Transcribed locus			1.06	0.07	9.4E-01	4.9E-03	1.00	1.00	0.00	1.00
1372280_at	ankyrin repeat and SOCS box-containing protein 2	Asb2	299266	1.07	0.07	8.6E-01	1.7E-04	1.00	1.00	0.80	1.00
1373436_at	similar to RIKEN cDNA 1700040L02 (predicted)	RGD1306739_predicted	361834	0.64	0.06	2.8E-01	5.6E-03	1.00	1.00	0.00	1.00
1374814_at				0.57	0.06	6.7E-02	9.5E-04	1.00	1.00	0.70	1.00
1388741_at	similar to cardiomyopathy associated 5	LOC683555	683555	1.72	0.06	3.3E-01	1.0E-03	1.00	1.00	0.20	1.00
1382775_at	ryanodine receptor 2, cardiac	Ryr2	498783	1.19	0.06	7.7E-01	1.4E-02	1.00	1.00	0.40	1.00
1375983_at	MAM domain containing 2	Mamdc2	309410	0.67	0.06	7.0E-01	2.9E-04	0.20	0.25	0.00	1.00
1372737_at	Similar to mKIAA0613 protein (predicted)	RGD1564875_predicted	498587	1.42	0.06	3.1E-01	6.4E-04	1.00	1.00	0.00	1.00
1377499_a_at	histidine rich calcium binding protein	Hrc	292905	0.95	0.06	8.4E-01	8.6E-04	1.00	1.00	0.80	1.00
1390461_at	Transcribed locus			0.34	0.06	2.0E-01	1.1E-04	0.00	0.00	0.00	1.00
1368380_at	vitronectin	Vtn	29169	0.82	0.06	5.2E-01	1.7E-03	1.00	1.00	0.00	1.00
1371984_at	similar to mKIAA0613 protein (predicted)	RGD1564875_predicted	498587	1.10	0.06	5.9E-01	5.7E-04	1.00	1.00	1.00	1.00
1374672_at	cardiac ankyrin repeat kinase	Cark	295531	1.15	0.06	6.3E-01	1.6E-04	1.00	1.00	0.20	1.00
1389707_at	Transcribed locus			3.94	0.06	1.5E-01	2.3E-04	0.20	0.00	0.00	0.75
1388557_at	complement component 7	C7	117517	1.25	0.06	5.4E-01	2.9E-03	1.00	1.00	1.00	1.00
1390473_at	potassium voltage-gated channel, subfamily G, member 2	Kcng2	307234	0.83	0.05	6.6E-01	7.6E-03	1.00	1.00	0.00	1.00
1371732_at	dermatopontin (predicted)	Dpt_predicted	289178	0.90	0.05	5.4E-01	8.6E-04	1.00	1.00	1.00	1.00
1373108_at	protein phosphatase 1, regulatory (inhibitor) subunit 3C	Ppp1r3c	309513	1.43	0.05	2.1E-01	5.4E-03	1.00	1.00	0.00	1.00
1373987_at	Kv channel-interacting protein 2	Kcnip2	56817	1.30	0.05	4.9E-01	1.8E-03	1.00	1.00	0.70	1.00
1377065_at	Transcribed locus, strongly similar to XP_573855.1 similar to chromosome 10 open reading frame 71			0.90	0.05	6.5E-01	1.5E-03	1.00	1.00	0.00	1.00
1389808_at	hypothetical protein LOC688274	LOC688274	688274	0.53	0.05	2.4E-01	1.5E-03	1.00	1.00	0.00	1.00
1389306_at	Transcribed locus, weakly similar to XP_001073995.1 similar to Prostatic steroid-binding protein C1 chain precursor	Cd151	64315	1.13	0.05	9.4E-01	2.8E-04	0.00	0.13	0.00	1.00
1376884_a_at	ribosomal protein L3-like (predicted)	Rpl3l_predicted	287122	0.93	0.05	7.9E-01	6.2E-04	1.00	1.00	0.60	1.00
1389079_at	dehydrogenase/reductase (SDR family) member 7C (predicted)	Dhrs7c_predicted	287411	0.64	0.05	2.9E-01	2.5E-03	1.00	1.00	0.50	1.00
1370363_at	carboxylesterase 3	Ces3	113902	0.83	0.05	6.4E-01	3.8E-03	1.00	1.00	0.00	1.00
1392652_at	Transcribed locus			0.85	0.05	7.9E-01	4.4E-05	0.80	1.00	0.00	1.00
1389905_at				1.58	0.05	1.7E-01	2.4E-03	1.00	1.00	0.00	1.00
1390450_a_at	osteoglycin (predicted)	Ogn_predicted	291015	1.12	0.04	8.6E-01	3.1E-03	1.00	1.00	0.40	1.00
1375303_at	similar to mKIAA0613 protein (predicted)	RGD1564875_predicted	498587	0.96	0.04	9.3E-01	1.9E-04	1.00	1.00	1.00	1.00
1374168_at	Transcribed locus			0.41	0.04	1.4E-02	1.3E-03	1.00	1.00	0.00	1.00
1385248_a_at	osteoglycin (predicted)	Ogn_predicted	291015	1.98	0.04	2.8E-01	2.9E-03	1.00	1.00	0.00	1.00
1389448_at	septin 4	Sept4	287606	0.71	0.04	5.0E-01	6.7E-04	1.00	1.00	0.00	1.00
1375982_at	similar to mKIAA0613 protein (predicted)	RGD1564875_predicted	498587	0.83	0.04	5.6E-01	8.4E-04	1.00	1.00	0.60	1.00
1371700_at	microfibrillar-associated protein 4	Mfap4	287382	0.55	0.03	3.2E-01	4.9E-03	0.90	1.00	0.40	1.00
1368093_at	myosin, heavy polypeptide 6, cardiac muscle, alpha	Myh6	29556	0.60	0.03	1.8E-01	3.1E-03	1.00	1.00	1.00	1.00
1391007_s_at	potassium inwardly rectifying channel, subfamily J, member 11	Kcnj11	83535	0.79	0.02	5.5E-01	1.3E-04	1.00	1.00	0.00	1.00
1389464_at	ligand of numb-protein X 1 (predicted)	Lnx1_predicted	360926	0.71	0.02	1.2E-01	4.4E-05	1.00	1.00	0.00	1.00
1383263_at	osteoglycin (predicted)	Ogn_predicted	291015	0.46	0.01	9.8E-02	9.5E-04	0.50	1.00	0.00	1.00
1367951_at	phosphoglycerate mutase 2	Pgam2	24959	0.61	0.01	3.3E-01	1.1E-03	1.00	1.00	0.00	1.00
1387049_at	Sideroflexin 2	Sfxn2	294011	1.24	0.01	7.0E-01	4.4E-05	1.00	1.00	0.00	1.00

C. Overlap

1370428_x_at	RT1 class Ib, locus Aw2	RT1-Aw2	24737	42.93	57.55	3.1E-03	4.4E-05	1.00	0.25	1.00	0.75
1367928_at	myosin, heavy polypeptide 7, cardiac muscle, beta	Myh7	29557	9.20	0.17	2.6E-02	1.3E-02	1.00	1.00	1.00	1.00
1371237_a_at	metallothionein 1a	Mt1a	24567	4.65	6.04	3.9E-02	4.1E-04	1.00	1.00	1.00	1.00
1370773_a_at	Kv channel-interacting protein 2	Kcnip2	56817	3.82	0.10	4.4E-02	2.5E-03	1.00	1.00	0.00	1.00

Probe Set ID	Gene Title	Gene Symbol	Entrez Gene ID	Fold Change<br>Strain Effect<br>Native BN / DA	Rejection Effect<br>Allograft / Isograft	False Discovery Rate<br>Native BN / DA	Allograft / Isograft	Average Present Call<br>Native BN	Native DA	Allograft	Isograft
1369050_at	phosphatidylinositol 3-kinase, C2 domain containing, gamma polypeptide	Pik3c2g	116720	0.30	0.23	2.5E-03	5.1E-03	0.00	1.00	0.20	1.00
1368304_at	flavin containing monooxygenase 3	Fmo3	84493	0.26	0.06	8.5E-02	1.1E-03	0.00	1.00	0.00	1.00
1376749_at	osteoglycin (predicted)	Ogn_predicted	291015	0.23	0.03	8.4E-03	1.4E-04	1.00	1.00	0.80	1.00
1370176_at	trafficking protein, kinesin binding 2	Trak2	171086	0.22	0.22	4.4E-04	2.3E-04	1.00	1.00	1.00	1.00
1375861_at	similar to nucleosome assembly protein 1-like 5	LOC682844	682844	0.17	0.17	3.8E-02	1.3E-03	0.30	1.00	0.00	1.00
1371245_a_at	beta-globin	LOC689064	689064	0.08	0.16	9.1E-03	1.3E-02	1.00	1.00	1.00	1.00
1374527_at	enoyl Coenzyme A hydratase domain containing 2 (predicted)	Echdc2_predicted	298381	0.01	0.15	9.3E-06	2.1E-03	0.00	1.00	0.00	1.00
1368637_at	caspase recruitment domain family, member 9	Card9	64171	0.02	0.09	1.0E-02	2.9E-03	0.00	1.00	0.00	1.00
1393130_at	Regulator of chromosome condensation (RCC1) and BTB (POZ) domain containing protein 1	Rcbtb1	361050	0.02	0.07	4.4E-04	1.4E-04	0.00	1.00	0.20	1.00
