# Supplementary material for: Impact of animal strain on gene expression in a rat model of acute cardiac rejection
Source: BMC Genomics. 2009 Jun 24;10:280. doi: 10.1186/1471-2164-10-280 (PMC2711118; doi:10.1186/1471-2164-10-280)
Supplement: Additional file 2 — Peripheral Blood Mononuclear Cells: Alterations in Gene Expression due to Strain Effect and Strain + Rejection Effect (color coded based on Venn diagram, Figure 3B). This table lists probesets manifesting differential expression attributable to strain in untransplanted animals, strain plus rejection in transplanted animals or both for peripheral blood mononuclear cells (PBMC). Differences in PBMC gene expression were assessed by ANOVA. A false discovery rate of 10%, a present call of at least 50% in either of the two groups being compared, and a fold change of at least 3 was required to declare a probeset as differentially expressed. [file 1471-2164-10-280-S2.pdf]

Additional File 2. Peripheral Blood Mononuclear Cells: Alterations in Gene Expression due to Strain Effect and Strain + Rejection Effect (color coded based on Venn diagram, Figure 3B)

				Fold Change		False Discovery Rate		Average Present Call			
				Strain Effect	Strain+Rejection Effect						
Probe Set ID	Gene Title	Gene Symbol	Entrez Gene ID	Native BN / DA	Allograft / Isograft	Native BN / DA	Allograft / Isograft	Native BN	Native DA	Allograft	Isograft
**A. Strain Effect**											
1368053_at	par-3 (partitioning defective 3) homolog (C. elegans)	Pard3	81918	24.70	13.34	3.0E-02	8.7E-05	1.00	0.00	0.20	0.00
1375497_at	Transcribed locus			21.16	2.99	2.8E-02	2.6E-02	0.50	0.00	0.10	0.00
1394841_at	Translocase of inner mitochondrial membrane 9 homolog (yeast)	Timm9	171139	17.91	2.20	4.8E-02	1.5E-01	0.67	0.00	0.00	0.00
1371621_at	Transcribed locus			10.39	2.18	4.6E-02	2.8E-01	1.00	0.00	0.60	0.00
1375842_at	similar to Glycophorin	LOC688972	688972	10.07	0.55	7.1E-02	4.2E-01	1.00	0.33	1.00	1.00
1378865_at	Transcribed locus			9.24	0.80	4.1E-02	6.2E-01	0.50	0.00	0.00	0.00
1371527_at	epithelial membrane protein 1	Emp1	25314	6.72	3.00	2.5E-02	2.2E-04	1.00	1.00	1.00	1.00
1381532_at	Lymphoid enhancer binding factor 1	Lef1	161452	6.25	7.42	7.8E-02	1.6E-02	1.00	0.00	0.00	0.00
1398365_at	similar to RIKEN cDNA 2700055K07	RGD1305061	291966	5.80	2.34	3.9E-02	1.4E-03	1.00	0.00	1.00	0.11
1369555_at	chemokine (C-C motif) receptor 4	Ccr4	171054	5.56	3.33	5.1E-02	1.8E-03	1.00	0.00	0.00	0.00
1371947_at	necdin	Ndn	308690	5.00	1.71	6.1E-02	3.7E-01	0.67	0.00	0.00	0.00
1386420_at	Transcribed locus			5.00	1.99	3.3E-02	3.0E-01	1.00	0.00	1.00	0.50
1390119_at	secreted frizzled-related protein 2	Sfrp2	310552	4.99	2.06	3.8E-02	3.4E-02	1.00	0.67	1.00	0.00
1395357_at	microtubule-associated protein 1b	Map1b	29456	4.99	2.26	5.3E-02	1.7E-01	0.50	0.00	0.40	0.00
1378419_at	pre-eosinophil-associated ribonuclease-2	LOC474169	474169	4.82	1.15	3.2E-02	6.5E-01	1.00	1.00	1.00	0.89
1383365_at	procollagen, type XVII, alpha 1 (predicted)	Col17a1_predicted	294027	4.60	2.68	3.6E-02	3.4E-05	1.00	0.67	1.00	0.00
1374519_at	Gamma-glutamyl carboxylase	Ggcx	81716	4.58	2.10	6.8E-02	2.2E-04	1.00	0.17	1.00	0.00
1382153_at	C-type (calcium dependent, carbohydrate recognition domain) lectin, superfamily member 6	Clecsf6	474143	4.58	2.10	4.0E-02	4.8E-02	1.00	0.50	1.00	0.78
1368491_at	deoxyribonuclease II beta	Dnase2b	59296	4.24	1.54	7.2E-02	2.2E-01	1.00	0.17	1.00	0.56
1385465_at	Sialic acid binding Ig-like lectin 5 (predicted)	Siglec5_predicted	292843	4.23	2.99	6.9E-02	1.6E-02	1.00	0.67	1.00	0.67
1392648_at	mannose receptor, C type 1 (predicted)	Mrc1_predicted	291327	4.18	2.38	9.7E-02	9.8E-03	1.00	0.67	1.00	0.44
1369086_a_at	calcium channel, voltage-dependent, L type, alpha 1D subunit	Cacna1d	29716	4.08	1.96	6.9E-02	1.9E-02	1.00	0.00	0.40	0.06
1396713_at	Similar to RIKEN cDNA 2610110G12	RGD1303066	361789	4.02	2.38	3.3E-02	1.7E-02	1.00	0.00	1.00	0.17
1386009_at	Colony stimulating factor 3 receptor (granulocyte) (predicted)	Csf3r_predicted	298518	3.88	1.93	6.9E-02	5.4E-03	1.00	1.00	1.00	1.00
1393227_at				3.87	2.74	5.0E-02	1.2E-03	1.00	1.00	1.00	1.00
1369736_at	epithelial membrane protein 1	Emp1	25314	3.78	4.67	7.9E-02	1.9E-02	1.00	0.00	0.40	0.00
1370613_s_at	UDP glycosyltransferase 1 family, polypeptide A1	Ugt1a1	113992	3.78	2.70	4.6E-02	1.0E-02	1.00	1.00	1.00	0.89
1396672_at				3.66	1.30	9.7E-02	1.7E-01	1.00	1.00	1.00	0.78
1388728_at	lysosomal-associated protein transmembrane 4B	Laptm4b	315047	3.66	2.40	5.5E-02	5.3E-05	1.00	1.00	1.00	1.00
1377671_at	Transcribed locus			3.55	2.43	2.7E-02	3.8E-03	1.00	1.00	1.00	1.00
1387947_at	v-maf musculoaponeurotic fibrosarcoma oncogene family, protein B (avian)	Mafb	54264	3.53	1.83	6.3E-02	9.6E-03	1.00	1.00	1.00	1.00
1388416_at	low density lipoprotein receptor-related protein 1	Lrp1	299858	3.52	1.85	5.0E-02	2.1E-03	1.00	1.00	1.00	1.00
1368000_at	complement component 3	C3	24232	3.51	1.73	2.7E-02	3.6E-02	1.00	1.00	1.00	1.00
1375055_at	similar to JP-45 protein	LOC686460	686460	3.43	4.86	9.3E-02	4.8E-02	1.00	0.00	0.00	0.00
1382201_at	unc-5 homolog C (C. elegans)	Unc5c	362049	3.42	1.11	9.5E-02	7.4E-01	0.83	0.00	1.00	0.89
1383936_at	Elastin microfibril interfacer 2 (predicted)	Emilin2_predicted	316736	3.40	2.05	3.7E-02	1.3E-03	1.00	1.00	1.00	1.00
1395301_x_at	Transcribed locus, strongly similar to XP_580064.1 hypothetical protein XP_580064 [Rattus norvegicus]	Fbxl4_predicted	313101	3.38	2.19	3.3E-02	7.9E-04	1.00	1.00	1.00	1.00
1395788_at	Transcribed locus			3.35	2.31	6.3E-02	3.2E-01	1.00	0.00	0.00	0.00
1394611_at				3.30	1.66	6.3E-02	2.7E-02	1.00	0.00	1.00	0.61
1372691_at	uridine phosphorylase 1	Upp1	289801	3.29	2.36	3.8E-02	4.5E-03	1.00	0.50	1.00	1.00
1376771_at	Transcribed locus			3.27	1.35	4.8E-02	6.3E-01	1.00	0.00	0.60	0.22
1369375_a_at	calpain 3	Capn3	29155	3.26	1.55	5.8E-02	1.1E-01	1.00	1.00	1.00	0.67
1388272_at	immunoglobulin heavy chain 1a (serum IgG2a)	Igh-1a	299352	3.21	0.46	4.1E-02	1.7E-01	1.00	1.00	1.00	1.00
1382905_at	Collagen-binding factor Endo180	Endo180	498011	3.21	1.28	2.8E-02	4.0E-01	1.00	1.00	1.00	1.00
1378285_at	natural cytotoxicity triggering receptor 3	Ncr3	294251	3.20	1.97	3.6E-02	5.5E-03	1.00	1.00	1.00	1.00
1387029_at	complement component factor H	Cfh	155012	3.19	2.32	7.8E-02	1.2E-03	1.00	1.00	1.00	1.00
1368393_at	complement component 1, q subcomponent, receptor 1	C1qr1	84398	3.17	2.25	6.0E-02	1.0E-02	1.00	1.00	1.00	1.00
1390102_at	disrupted in renal carcinoma 2 homolog (human)	Dirc2	303902	3.15	2.44	3.3E-02	9.4E-05	1.00	1.00	1.00	1.00
1377064_at	dual specificity phosphatase 6	Dusp6	116663	3.12	1.36	6.1E-02	2.7E-02	1.00	1.00	1.00	1.00
1374794_at	kinesin family member 15	Kif15	353302	3.12	2.93	3.0E-02	3.9E-04	1.00	1.00	1.00	0.78
1373204_at	hypothetical LOC297077	RGD1310725	297077	3.06	2.34	4.6E-02	6.4E-04	1.00	1.00	1.00	1.00
1378418_at	similar to MGC37193 protein	RGD1311681	364674	3.06	2.54	6.3E-02	4.9E-04	1.00	1.00	1.00	1.00
1379625_at	Transcribed locus			3.06	1.50	3.8E-02	2.1E-01	1.00	0.00	0.80	0.72
1370491_a_at	histidine decarboxylase	Hdc	24443	3.04	2.30	8.3E-02	1.2E-02	1.00	0.00	0.50	0.00
1369089_at	protein kinase C, gamma	Prkcc	24681	3.04	1.19	9.1E-02	5.0E-01	0.83	0.00	0.00	0.00
1385129_at	similar to mitochondrial glycerol 3-phosphate acyltransferase (predicted)	RGD1304904_predicted	296130	3.02	2.30	3.8E-02	1.2E-01	1.00	0.50	0.80	0.17
1379642_at	Transcribed locus			3.02	2.28	7.7E-02	4.2E-04	1.00	1.00	1.00	1.00
1373082_at	Transcribed locus			0.33	0.36	6.8E-02	4.2E-04	0.00	0.67	0.00	1.00
1384180_at	interferon-induced protein with tetratricopeptide repeats 2	Ifit2	294091	0.33	0.66	8.9E-02	8.7E-02	1.00	1.00	1.00	1.00
1371951_at	Transcribed locus			0.33	0.57	3.4E-02	2.7E-02	1.00	1.00	1.00	1.00
1391495_at	Similar to RIKEN cDNA 2010012O05 (predicted)	RGD1311783_predicted	294012	0.33	0.78	1.9E-02	1.5E-01	1.00	1.00	0.40	1.00
1393887_at	choline phosphotransferase 1	Chpt1	362866	0.33	0.43	8.1E-02	2.5E-03	0.00	1.00	0.50	0.61
1387831_at	Inhibitor of Bruton agammaglobulinemia tyrosine kinase (predicted)	Ibtk_predicted	315858	0.32	0.34	2.9E-02	3.3E-03	1.00	1.00	1.00	1.00
1394154_at	Transcribed locus			0.32	0.87	6.8E-02	6.2E-01	0.17	1.00	0.30	0.44
1389832_at	glutathione S-transferase omega 1	Gsto1	114846	0.32	0.47	3.0E-02	1.8E-04	1.00	1.00	1.00	1.00
1383503_at	Transcribed locus			0.32	0.67	5.5E-02	3.7E-02	0.33	1.00	0.20	1.00
1377697_at	similar to amyloid beta (A4) precursor protein-binding, family B, member 2 (predicted)	RGD1562438_predicted	305338	0.32	0.36	2.5E-02	1.0E-03	1.00	1.00	1.00	1.00
1379285_at	receptor transporter protein 4 (predicted)	Rtp4_predicted	360733	0.31	0.64	3.5E-02	1.1E-01	1.00	1.00	1.00	1.00
1383393_at	Transcribed locus			0.31	0.62	4.4E-02	1.5E-02	1.00	1.00	1.00	1.00
1398664_at	GRAM domain containing 3	Gramd3	307288	0.31	0.34	7.8E-02	1.2E-03	1.00	1.00	0.80	1.00
1385643_at	Transcribed locus			0.31	0.47	7.7E-02	2.6E-02	1.00	1.00	0.20	1.00
1397907_at	Hypothetical protein LOC501546	LOC501546	501546	0.31	0.42	6.4E-02	1.2E-02	1.00	1.00	0.80	1.00
1377133_at	Hypothetical protein LOC680687	LOC680687	680687	0.31	0.43	3.2E-02	2.1E-03	1.00	1.00	0.80	1.00
1370628_at	granzyme B	Gzmb	171528	0.30	0.75	6.1E-02	1.8E-01	1.00	1.00	1.00	1.00

Probe Set ID	Gene Title	Gene Symbol	Entrez Gene ID	Fold Change: Strain Effect Native BN / DA	Fold Change: Strain+Rejection Effect Allograft / Isograft	False Discovery Rate Native BN / DA	False Discovery Rate Allograft / Isograft	Average Present Call Native BN	Average Present Call Native DA	Average Present Call Allograft	Average Present Call Isograft
1369857_a_at	solute carrier family 14 (urea transporter), member 1	Slc14a1	54301	0.30	0.62	8.2E-02	1.1E-01	1.00	1.00	0.10	0.67
1384933_at	Transcribed locus			0.30	0.35	8.3E-02	9.1E-03	0.17	1.00	0.30	1.00
1383564_at	interferon regulatory factor 7	Irf7	293624	0.30	0.35	5.1E-02	7.6E-05	1.00	1.00	1.00	1.00
1394500_at	Transcribed locus			0.29	0.62	7.6E-02	5.4E-03	0.33	1.00	1.00	1.00
1392745_at	similar to RIKEN cDNA 3110040N11	RGD1305713	293059	0.29	0.49	2.6E-02	2.7E-02	1.00	1.00	0.00	1.00
1380415_at	RT1 class Ib, locus Aw2	RT1-Aw2	24737	0.29	0.41	4.2E-02	1.1E-05	1.00	1.00	1.00	1.00
1395116_at	Procollagen, type IX, alpha 1	Col9a1	305104	0.29	0.52	4.0E-02	2.1E-04	1.00	1.00	1.00	1.00
1373028_at	receptor-like tyrosine kinase	Ryk	140585	0.29	0.40	3.3E-02	2.3E-04	0.00	1.00	0.00	1.00
1397300_at	Transcribed locus			0.29	0.54	2.5E-02	1.3E-02	1.00	1.00	1.00	1.00
1383097_at	similar to RIKEN cDNA 2010012O05 (predicted)	RGD1311783_predicted	294012	0.29	0.51	3.5E-02	6.1E-03	1.00	1.00	1.00	1.00
1395274_at	dystonin (predicted)	Dst_predicted	316313	0.28	0.38	4.0E-02	5.9E-02	1.00	1.00	0.40	0.78
1371152_a_at	2',5'-oligoadenylate synthetase 1, 40/46kDa	Oas1	192281	0.28	1.33	4.5E-02	2.0E-01	1.00	1.00	1.00	1.00
1384067_at	FCH domain only 2 (predicted)	Fcho2_predicted	309129	0.28	0.34	5.0E-02	2.2E-04	0.67	1.00	0.90	1.00
1371101_at	receptor-like tyrosine kinase	Ryk	140585	0.28	0.61	7.3E-02	8.1E-02	1.00	1.00	0.90	1.00
1380888_at	similar to CG31613-PA	LOC684841	684841	0.28	0.36	9.8E-02	4.3E-03	0.33	1.00	0.00	1.00
1370013_at	cyclic nucleotide gated channel alpha 1	Cnga1	85259	0.28	0.39	6.6E-02	4.9E-04	0.17	1.00	0.00	1.00
1368762_at	ubiquitin D	Ubd	29168	0.27	0.40	4.3E-02	1.3E-05	0.33	1.00	0.40	1.00
1387995_a_at	interferon induced transmembrane protein 3	Ifitm3	361673	0.27	0.89	7.7E-02	5.9E-01	1.00	1.00	1.00	1.00
1380094_a_at	Zinc finger protein 212	Zfp212	297066	0.27	0.50	2.6E-02	2.3E-03	0.83	1.00	0.40	1.00
1382314_at	interferon, alpha-inducible protein (clone IFI-15K) (predicted)	G1p2_predicted	298693	0.27	0.39	6.0E-02	5.5E-04	1.00	1.00	0.90	1.00
1368409_at	glutathione S-transferase, theta 2	Gstt2	29487	0.27	0.29	2.7E-02	2.2E-04	0.00	1.00	0.00	0.22
1371017_at	Glycerol-3-phosphate acyltransferase, mitochondrial	Gpam	29653	0.27	0.35	1.4E-02	1.4E-04	1.00	1.00	1.00	1.00
1389470_at	complement factor B	Cfb	294257	0.27	0.91	6.9E-02	7.3E-01	0.00	1.00	1.00	1.00
1373833_at	similar to RIKEN cDNA 3110040N11	RGD1305713	293059	0.26	0.38	3.8E-02	5.4E-05	1.00	1.00	1.00	1.00
1387739_at	CD8 antigen, beta chain	Cd8b	24931	0.26	0.55	1.4E-02	1.4E-03	1.00	1.00	1.00	1.00
1369877_at	CD8 antigen, alpha chain	Cd8a	24930	0.26	0.41	2.5E-02	3.0E-04	1.00	1.00	1.00	1.00
1375724_at	similar to testhymin (predicted)	RGD1563612_predicted	500026	0.26	0.34	2.6E-02	1.4E-04	1.00	1.00	1.00	1.00
1385414_at	CD8 antigen, alpha chain	Cd8a	24930	0.25	0.39	2.5E-02	4.0E-04	1.00	1.00	1.00	1.00
1387283_at	myxovirus (influenza virus) resistance 2	Mx2	286918	0.25	0.46	7.3E-02	1.2E-03	1.00	1.00	1.00	1.00
1385314_at	Transcribed locus			0.25	0.81	5.0E-02	1.6E-01	1.00	1.00	1.00	1.00
1398706_at				0.24	0.49	2.6E-02	1.5E-03	1.00	1.00	1.00	1.00
1387366_at	interleukin enhancer binding factor 3	Ilf3	84472	0.24	0.60	2.5E-02	7.4E-03	1.00	1.00	1.00	1.00
1394027_at	similar to Nucleoporin 62 (predicted)	RGD1564353_predicted	300923	0.24	0.60	1.9E-02	2.2E-02	1.00	1.00	1.00	1.00
1397538_at	similar to CG3996-PA (predicted)	RGD1305255_predicted	299824	0.24	0.26	3.9E-02	4.7E-02	0.00	0.83	0.00	0.06
1396771_at	Transcribed locus			0.23	0.47	6.1E-02	7.0E-02	1.00	1.00	0.80	1.00
1379293_at	granzyme A	Gzma	266708	0.23	0.70	3.0E-02	1.9E-01	1.00	1.00	1.00	1.00
1367899_at	coagulation factor II (thrombin) receptor	F2r	25439	0.23	0.41	2.6E-02	5.9E-04	1.00	1.00	1.00	1.00
1393245_at	Phytanoyl-CoA hydroxylase	Phyh	114209	0.23	0.36	2.3E-02	9.9E-06	1.00	1.00	1.00	1.00
1380638_at	nuclear receptor coactivator 3	Ncoa3	84584	0.23	0.67	4.7E-02	9.9E-03	1.00	1.00	1.00	1.00
1382155_at	COX15 homolog, cytochrome c oxidase assembly protein (yeast)	Cox15	309391	0.23	0.87	2.9E-02	6.0E-01	1.00	1.00	0.80	1.00
1372002_at	gap junction membrane channel protein alpha 1	Gja1	24392	0.22	0.46	2.5E-02	1.2E-03	0.33	1.00	0.10	1.00
1382024_at	Similar to RIKEN cDNA 2010315L10 (predicted)	RGD1306660_predicted	290627	0.22	0.61	3.2E-02	3.9E-02	1.00	1.00	1.00	1.00
1392716_at	Transcribed locus			0.22	0.41	3.3E-02	1.6E-03	0.00	1.00	0.60	1.00
1379794_at	granzyme B	Gzmb	171528	0.21	0.64	4.5E-02	4.4E-02	1.00	1.00	1.00	1.00
1393657_at	prolylcarboxypeptidase (angiotensinase C) (predicted)	Prcp_predicted	293118	0.21	1.02	3.2E-02	9.7E-01	0.00	1.00	1.00	1.00
1381042_at	Transcribed locus			0.20	0.36	8.9E-02	3.4E-04	1.00	1.00	0.60	1.00
1379436_a_at	RT1 class Ib, locus Aw2	RT1-Aw2	24737	0.20	0.40	3.2E-02	1.4E-04	1.00	1.00	0.70	1.00
1368598_at	somatostatin receptor 3	Sstr3	171044	0.17	0.39	7.9E-02	4.8E-04	1.00	1.00	1.00	1.00
1374708_at	similar to Rho guanine nucleotide exchange factor (GEF) 10 (predicted)	RGD1565043_predicted	306618	0.17	0.39	4.8E-02	1.9E-06	1.00	1.00	1.00	1.00
1368042_a_at	high mobility group box 1	Hmgb1	25459	0.17	0.68	2.7E-02	9.4E-03	1.00	1.00	1.00	1.00
1374015_at	similar to potassium voltage-gated channel, Isk-related family, member 1-like	LOC681190	681190	0.17	0.35	2.6E-02	3.5E-04	1.00	1.00	1.00	1.00
1370822_at	RT1 class II, locus Ba	RT1-Ba	309621	0.17	0.60	3.2E-02	8.8E-02	1.00	1.00	1.00	1.00
1380084_at	Similar to solute carrier family 25, member 36	LOC501039	501039	0.16	0.33	7.0E-02	2.1E-04	0.67	1.00	0.80	1.00
1378233_at	Transcribed locus			0.15	0.16	9.5E-02	1.5E-04	0.00	1.00	0.00	0.17
1397146_at				0.12	0.65	2.1E-02	3.9E-01	0.00	0.67	0.00	0.00
1392707_at	Transcribed locus, weakly similar to XP_917161.1 hypothetical protein XP_912068 [Mus musculus]			0.12	0.58	7.3E-02	3.1E-01	0.00	0.50	0.00	0.00
1396551_at	Similar to CG33154-PB (predicted)	RGD1310270_predicted	314798	0.11	0.37	5.9E-02	4.0E-02	0.00	1.00	0.00	0.22
1398468_at	Transcribed locus			0.11	0.41	2.5E-02	2.7E-02	0.00	0.67	0.00	0.00
1394277_at				0.11	0.72	8.3E-02	6.7E-01	0.00	0.67	0.00	0.00
1376908_at	interferon-induced protein with tetratricopeptide repeats 3	Ifit3	309526	0.11	0.35	3.6E-02	4.0E-03	1.00	1.00	1.00	1.00
1380119_at	Transcribed locus			0.10	0.34	3.1E-02	1.3E-01	0.00	0.67	0.00	0.00
1397473_at	distrobrevin binding protein 1	Dtnbp1	641528	0.10	0.75	9.7E-02	3.6E-01	0.00	1.00	0.00	0.00
1393397_at	carboxypeptidase A2 (pancreatic) (predicted)	Cpa2_predicted	296959	0.09	0.15	4.3E-02	1.5E-04	0.00	0.83	0.00	0.00
1379846_at	Transcribed locus			0.07	0.23	9.8E-02	1.2E-02	0.00	1.00	0.00	0.00
1375030_at	Transcribed locus			0.07	0.17	7.3E-02	1.5E-03	0.00	1.00	0.00	0.17
1395942_at				0.07	0.69	5.0E-02	9.3E-02	0.00	1.00	0.00	0.72
1376892_at	CDNA clone IMAGE:7313785			0.05	0.42	3.2E-02	6.1E-04	0.00	1.00	0.30	1.00
1395888_at	Thyroid hormone receptor associated protein 6 (predicted)	Thrap6_predicted	299905	0.04	0.27	2.6E-02	1.5E-02	0.00	0.67	0.00	0.00
1390911_at	RT1 class Ib, locus Aw2	RT1-Aw2	24737	0.03	0.03	2.6E-02	4.5E-06	0.00	1.00	0.00	0.00
1378193_at	membrane-spanning 4-domains, subfamily A, member 7 (predicted)	Ms4a7_predicted	293744	0.03	0.91	2.9E-02	9.3E-01	0.00	1.00	0.80	0.83
1380651_at	ring finger protein (C3H2C3 type) 6 (predicted)	Rnf6_predicted	304271	0.01	0.40	9.6E-03	1.1E-03	0.00	1.00	1.00	1.00

B. Strain+Rejection Effect

Probe Set ID	Gene Title	Gene Symbol	Entrez Gene ID	Native BN / DA	Allograft / Isograft	FDR Native BN / DA	FDR Allograft / Isograft	Native BN	Native DA	Allograft	Isograft
1371081_at	Rap guanine nucleotide exchange factor (GEF) 4	Rapgef4	252857	9.34	21.28	2.5E-01	8.6E-04	1.00	0.00	1.00	0.00
1391018_at	Transcribed locus			5.52	15.12	2.2E-01	3.5E-03	0.67	0.00	1.00	0.00
1382788_at	Major histocompatibility complex, class II, DM beta	Hla-dmb	294273	4.29	9.91	1.6E-01	8.3E-03	0.17	0.00	0.60	0.00
1370215_at	complement component 1, q subcomponent, beta polypeptide	C1qb	29687	0.39	9.82	6.9E-01	3.6E-02	0.00	0.00	0.80	0.00

Probe Set ID	Gene Title	Gene Symbol	Entrez Gene ID	Fold Change: Strain Effect: Native BN / DA	Fold Change: Strain+Rejection Effect: Allograft / Isograft	False Discovery Rate: Native BN / DA	False Discovery Rate: Allograft / Isograft	Average Present Call: Native BN	Average Present Call: Native DA	Average Present Call: Allograft	Average Present Call: Isograft
1394673_at	similar to Myeloid cell surface antigen CD33 precursor (Siglec-3)	LOC687856	687856	2.25	9.76	3.8E-02	1.4E-02	1.00	0.33	1.00	0.00
1387835_at	interleukin 1 receptor antagonist	Il1rn	60582	2.40	8.15	6.2E-01	3.8E-02	0.67	0.33	1.00	0.33
1378733_at	Transcribed locus			5.56	7.46	3.9E-01	9.0E-03	1.00	0.00	1.00	0.00
1385205_at	Transcribed locus			6.87	7.02	1.4E-01	1.4E-04	1.00	0.00	1.00	0.00
1396420_at	Bromodomain adjacent to zinc finger domain protein 1B	Baz1b	368002	15.86	6.15	1.1E-01	3.0E-02	1.00	0.33	0.80	0.11
1376652_at	complement component 1, q subcomponent, alpha polypeptide	C1qa	298566	0.44	5.59	5.7E-01	6.1E-02	0.00	0.00	0.80	0.06
1376100_at	tubulin, beta 6	Tubb6	307351	5.50	5.45	2.2E-01	2.0E-02	0.00	0.00	0.50	0.00
1387742_at	chemokine (C-C motif) receptor 2	Ccr2	60463	4.47	5.42	1.3E-01	2.8E-03	1.00	1.00	1.00	0.78
1372060_at				16.09	5.04	1.2E-01	2.6E-02	1.00	0.33	1.00	0.00
1368266_at	arginase 1	Arg1	29221	1.59	4.64	7.7E-01	7.6E-04	0.00	0.00	0.80	0.00
1382601_at	Similar to scavenger receptor type A SR-A (predicted)	RGD1564316_predicted	498638	1.63	4.60	5.7E-01	3.0E-02	0.33	0.00	1.00	0.33
1396133_at	Integrin, beta 5	Itgb5	257645	9.10	4.59	1.7E-01	1.0E-02	0.67	0.00	0.80	0.00
1387314_at	sulfotransferase family 1B, member 1	Sult1b1	64305	2.22	4.48	1.2E-01	2.3E-02	0.00	0.00	0.80	0.00
1393252_at	Transcribed locus			2.57	4.45	2.6E-01	1.6E-02	1.00	0.33	1.00	0.33
1384466_at	notch1-induced protein	LOC493574	493574	5.53	4.36	3.4E-01	1.4E-02	0.67	0.00	0.80	0.11
1397730_at	similar to RIKEN cDNA 2310035C23 (predicted)	RGD1307235_predicted	309053	1.63	4.23	7.2E-01	1.7E-02	1.00	0.67	1.00	0.22
1367860_a_at	matrix metallopeptidase 14 (membrane-inserted)	Mmp14	81707	0.59	4.22	6.4E-01	1.5E-02	0.00	0.00	0.90	0.00
1388142_at	chondroitin sulfate proteoglycan 2	Cspg2	114122	1.86	4.18	3.1E-01	3.4E-04	1.00	1.00	1.00	1.00
1373025_at	complement component 1, q subcomponent, gamma polypeptide	C1qg	362634	0.46	4.07	6.2E-01	1.9E-02	0.00	0.00	0.80	0.00
1393682_at	G-protein-coupled receptor GPR34 (predicted)	GPR34_predicted	554353	3.01	4.07	1.0E-01	5.0E-04	0.67	0.50	1.00	1.00
1369665_a_at	interleukin 18	Il18	29197	2.77	4.04	3.8E-02	3.4E-05	1.00	1.00	1.00	1.00
1369290_at	chemokine (C-C motif) receptor 5	Ccr5	117029	1.44	3.96	6.1E-01	5.3E-03	1.00	1.00	1.00	0.78
1388054_a_at	chondroitin sulfate proteoglycan 2	Cspg2	114122	2.30	3.95	2.2E-01	1.2E-03	1.00	0.33	1.00	0.78
1370728_at	interleukin 13 receptor, alpha 1	Il13ra1	252963	2.62	3.92	1.9E-01	4.1E-02	1.00	0.67	1.00	0.33
1394630_at	LIM domain only 4	Lmo4	362051	1.27	3.90	7.4E-01	8.1E-02	1.00	0.00	0.50	0.00
1368494_at	S100 calcium binding protein A8 (calgranulin A)	S100a8	116547	1.78	3.90	2.1E-01	1.9E-02	1.00	1.00	1.00	1.00
1371146_at	transcription factor EC	Tfec	26296	1.72	3.73	4.8E-01	7.5E-03	1.00	0.33	1.00	0.22
1390364_at	Transcribed locus			1.10	3.70	9.1E-01	5.9E-04	1.00	1.00	1.00	1.00
1383105_at	hypothetical protein LOC681292	LOC681292	681292	2.54	3.68	7.4E-02	2.1E-04	1.00	1.00	1.00	0.67
1378428_at	Transcribed locus			1.62	3.66	3.6E-01	9.2E-03	1.00	1.00	1.00	1.00
1383743_at	leucine rich repeat containing 16 (predicted)	Lrrc16_predicted	306941	6.08	3.58	2.1E-01	2.0E-03	1.00	0.33	1.00	0.22
1381613_at	Similar to farnesyl diphosphate synthetase	LOC501976	501976	2.27	3.51	7.7E-02	4.0E-02	1.00	0.00	1.00	0.00
1372031_at	Transcribed locus			1.07	3.51	8.4E-01	4.6E-04	0.00	0.00	1.00	0.00
1371232_a_at	chondroitin sulfate proteoglycan 2	Cspg2	114122	1.62	3.47	1.2E-01	1.4E-03	1.00	1.00	1.00	1.00
1391774_at	Transcribed locus			2.89	3.41	1.1E-01	1.5E-05	1.00	1.00	1.00	1.00
1368597_at	SNF1-like kinase	Snf1lk	59329	18.46	3.31	1.6E-01	1.2E-03	1.00	0.00	1.00	0.00
1394490_at	ATP-binding cassette, sub-family A (ABC1), member 1	Abca1	313210	1.73	3.31	1.8E-01	1.5E-04	1.00	0.67	1.00	1.00
1368187_at	glycoprotein (transmembrane) nmb	Gpnmb	113955	2.04	3.30	2.1E-01	2.6E-04	1.00	1.00	1.00	1.00
1397222_at	Tripartite motif protein 39	Trim39	309591	2.87	3.29	1.3E-01	6.4E-02	1.00	0.67	0.80	0.22
1377698_at	tumor necrosis factor receptor superfamily, member 5	Tnfrsf5	171369	2.67	3.25	3.9E-02	2.4E-04	1.00	1.00	1.00	0.56
1394861_at	POU domain, class 6, transcription factor 1	Pou6f1	116545	3.61	3.23	1.3E-01	4.0E-02	0.83	0.33	1.00	0.00
1392043_at	Insulin receptor	Insr	24954	2.21	3.21	4.0E-01	3.1E-03	1.00	0.00	1.00	0.56
1375003_at	serine (or cysteine) peptidase inhibitor, clade B, member 6a	Serpinb6a	291085	2.94	3.20	7.8E-02	1.4E-04	1.00	1.00	1.00	1.00
1394451_at	annexin A1	Anxa1	25380	2.84	3.19	3.6E-02	1.3E-05	1.00	0.33	1.00	0.00
1385892_at	Transcribed locus			1.00	3.14	9.9E-01	7.5E-02	1.00	0.83	0.90	0.17
1384479_at	UDP-N-acetyl-alpha-D-galactosamine:polypeptide N-acetylgalactosaminyltransferase 3	Galnt3	366061	2.69	3.13	1.5E-01	8.9E-02	1.00	0.00	1.00	0.00
1382129_at	Guanine nucleotide binding protein, alpha stimulating, olfactory type	Gnal	24611	2.24	3.11	3.8E-01	5.1E-02	1.00	0.67	1.00	0.61
1383506_at	B-cell CLL/lymphoma 11A (zinc finger protein)	Bcl11a	305589	2.74	3.11	9.8E-02	4.5E-02	1.00	1.00	1.00	0.06
1370382_at	RT1 class II, locus Bb	RT1-Bb	309622	1.99	3.08	5.1E-01	8.2E-03	1.00	0.67	1.00	1.00
1385759_at	serine (or cysteine) peptidase inhibitor, clade B (ovalbumin), member 10	Serpinb10	266775	2.39	3.08	1.8E-01	4.9E-04	1.00	1.00	1.00	1.00
1385871_at	DEAH (Asp-Glu-Ala-His) box polypeptide 36 (predicted)	Dhx36_predicted	310461	1.48	3.07	5.8E-01	7.1E-03	1.00	1.00	1.00	1.00
1391219_at	Transcribed locus			2.36	3.05	3.0E-01	3.3E-05	1.00	0.33	1.00	0.56
1390412_at	Pre-B lymphocyte gene 3 (predicted)	Vpreb3_predicted	365550	0.45	0.33	2.2E-01	7.6E-05	1.00	1.00	1.00	1.00
1387112_at	proteolipid protein	Plp	24943	0.57	0.33	3.6E-01	9.0E-05	1.00	1.00	1.00	1.00
1378738_at	potassium voltage-gated channel, shaker-related subfamily, beta member 1	Kcnab1	29737	0.41	0.33	1.4E-01	3.0E-05	1.00	1.00	1.00	1.00
1390807_at	carbonic anhydrase VB, mitochondrial	Ca5b	302669	0.34	0.33	4.7E-02	1.2E-05	1.00	1.00	1.00	1.00
1380831_at	Transcribed locus			0.45	0.33	1.4E-01	2.6E-05	1.00	1.00	1.00	1.00
1369097_s_at	guanylate cyclase 1, soluble, beta 3	Gucy1b3	25202	0.57	0.32	3.4E-01	3.3E-04	1.00	1.00	1.00	1.00
1382853_at	ring finger protein 11 (predicted)	Rnf11_predicted	316552	0.53	0.32	8.3E-02	5.6E-05	1.00	1.00	1.00	1.00
1384045_at				0.50	0.32	2.2E-01	8.4E-02	0.33	1.00	0.40	0.56
1395967_at	Transcribed locus			0.70	0.32	1.6E-01	8.4E-04	1.00	1.00	0.00	0.94
1384273_at	carbohydrate kinase-like	Carkl	287479	0.66	0.32	5.2E-01	3.3E-04	1.00	1.00	0.60	1.00
1371541_at	myosin, light polypeptide kinase (predicted)	Mylk_predicted	288057	0.51	0.32	1.7E-01	1.4E-04	1.00	1.00	1.00	1.00
1386275_at	Transcribed locus			0.39	0.32	6.8E-02	8.8E-05	1.00	1.00	1.00	1.00
1392817_at	Transcribed locus			0.28	0.32	5.1E-01	2.6E-02	0.00	0.00	0.40	0.94
1370418_s_at	brain and kidney protein	Bk	192189	0.39	0.32	7.2E-02	1.6E-05	0.00	0.00	0.00	1.00
1392801_at	similar to novel protein (predicted)	RGD1561145_predicted	498580	0.37	0.32	7.2E-02	1.1E-04	1.00	1.00	1.00	1.00
1377309_at	Transcribed locus			0.34	0.32	1.9E-01	3.7E-05	1.00	1.00	0.90	1.00
1376546_at	similar to hypothetical protein (predicted)	RGD1565432_predicted	499157	0.72	0.32	3.6E-01	1.7E-02	1.00	1.00	0.80	1.00
1387130_at	solute carrier family 39 (iron-regulated transporter), member 1	Slc40a1	170840	0.51	0.32	3.6E-01	7.1E-05	1.00	1.00	1.00	1.00
1394386_s_at	vacuolar protein sorting 52 (yeast)	Vps52	25218	0.52	0.32	5.6E-02	1.5E-02	1.00	1.00	0.00	0.67
1383240_at	integrin, alpha 6	Itga6	114517	0.44	0.32	6.1E-02	1.2E-04	1.00	1.00	1.00	1.00
1380600_at	similar to Integrin alpha-IIb precursor (Platelet membrane glycoprotein IIb) (GPalpha IIb) (GPIIb) (CD41 antigen)	LOC685269	685269	0.48	0.31	2.0E-01	1.9E-04	1.00	1.00	1.00	1.00
1368348_at	solute carrier family 6 (neurotransmitter transporter, serotonin), member 4	Slc6a4	25553	0.79	0.30	6.3E-01	1.4E-04	1.00	1.00	1.00	1.00
1388432_at	optineurin	Optn	246294	0.44	0.30	3.4E-02	3.8E-04	1.00	1.00	1.00	1.00
1388804_at	Transcribed locus			0.52	0.30	2.0E-01	2.3E-02	1.00	1.00	0.00	0.61
1369732_a_at	ST3 beta-galactoside alpha-2,3-sialyltransferase 2	St3gal2	64442	0.76	0.30	5.2E-01	3.4E-05	1.00	1.00	1.00	1.00

				Fold Change		False Discovery Rate		Average Present Call			
				Strain Effect	Strain+Rejection Effect						
Probe Set ID	Gene Title	Gene Symbol	Entrez Gene ID	Native BN / DA	Allograft / Isograft	Native BN / DA	Allograft / Isograft	Native BN	Native DA	Allograft	Isograft
1370548_at	solute carrier family 16 (monocarboxylic acid transporters), member 10	Slc16a10	170566	0.55	0.30	1.7E-01	7.8E-03	1.00	1.00	0.40	1.00
1398420_at	SMAD specific E3 ubiquitin protein ligase 2 (predicted)	Smurf2_predicted	303614	0.54	0.30	1.8E-01	1.1E-03	1.00	1.00	1.00	1.00
1370518_a_at	syntaxin binding protein 1	Stxbp1	25558	0.21	0.30	1.2E-01	3.3E-05	0.00	1.00	0.10	1.00
1374529_at	Thrombospondin 1	Thbs1	445442	0.54	0.30	1.4E-01	9.6E-06	1.00	1.00	1.00	1.00
1377029_at	RAR-related orphan receptor alpha (predicted)	Rora_predicted	300807	1.03	0.30	9.0E-01	8.5E-02	0.00	0.17	0.00	0.56
1377646_at	Transcribed locus			0.70	0.29	7.4E-01	3.7E-02	0.00	0.00	0.10	0.67
1370913_at	Best5 protein	Best5	65190	0.39	0.29	3.6E-01	1.7E-02	1.00	1.00	1.00	1.00
1381780_at	Transcribed locus			1.09	0.29	8.6E-01	2.1E-02	1.00	1.00	1.00	1.00
1379325_at	Fatty acid amide hydrolase	Faah	29347	0.52	0.29	2.0E-01	5.6E-04	1.00	1.00	1.00	1.00
1374139_at	cerebellar degeneration-related 2	Cdr2	308958	1.15	0.29	6.5E-01	2.6E-02	1.00	1.00	1.00	1.00
1379496_at	RT1 class Ib, locus Aw2	RT1-Aw2	24737	0.34	0.29	3.3E-02	3.4E-04	1.00	1.00	1.00	1.00
1394621_at	Similar to genetic suppressor element 1 (predicted)	RGD1562686_predicted	307913	0.36	0.29	3.6E-02	2.2E-06	1.00	1.00	1.00	1.00
1370138_at	lymphoid enhancer binding factor 1	Lef1	161452	0.61	0.29	3.6E-02	1.3E-04	1.00	1.00	1.00	1.00
1370163_at	ornithine decarboxylase 1	Odc1	24609	0.42	0.29	7.7E-02	2.6E-06	1.00	1.00	1.00	1.00
1383933_at	Similar to KIAA0564 protein (predicted)	RGD1308772_predicted	290381	0.60	0.29	2.1E-01	8.8E-06	1.00	1.00	1.00	1.00
1380100_at	similar to Traf2 and NCK interacting kinase, splice variant 4 (predicted)	RGD1561817_predicted	294917	0.46	0.28	6.5E-02	8.9E-06	1.00	1.00	0.10	1.00
1389654_at	Plastin 1 (I isoform) (predicted)	Pls1_predicted	315926	0.53	0.28	3.9E-01	1.2E-05	1.00	1.00	0.20	1.00
1390579_at	similar to RIKEN cDNA 1810029B16 (predicted)	RGD1305222_predicted	290686	0.58	0.28	5.5E-02	8.1E-05	1.00	1.00	0.60	1.00
1370152_at	glycoprotein 5, platelet	Gp5	25259	0.80	0.28	6.7E-01	3.2E-04	1.00	1.00	1.00	1.00
1372455_at	transmembrane 4 superfamily member 12	Tm4sf12	362326	0.57	0.28	3.1E-01	7.1E-05	1.00	1.00	1.00	1.00
1368755_at	C-type lectin domain family 4, member f	Clec4f	114598	0.37	0.28	2.1E-01	4.9E-04	1.00	1.00	0.50	1.00
1383399_at	synaptic vesicle glycoprotein 2a	Sv2a	117559	0.40	0.28	4.8E-02	2.1E-06	1.00	1.00	1.00	1.00
1373356_at				0.58	0.28	2.0E-01	1.0E-03	1.00	1.00	0.30	1.00
1370624_at	coagulation factor II (thrombin) receptor-like 2	F2rl2	29636	0.21	0.27	1.2E-01	1.3E-05	1.00	1.00	1.00	1.00
1387905_at	J domain protein 1	LOC619393	619393	0.41	0.27	6.3E-02	5.5E-06	1.00	1.00	1.00	1.00
1389398_at	ankyrin 1, erythroid	Ank1	306570	1.13	0.27	9.5E-01	6.8E-02	0.00	0.00	0.00	0.67
1378843_at	DnaJ (Hsp40) homolog, subfamily B, member 6	Dnajb6	362293	0.36	0.27	1.9E-01	3.1E-03	1.00	1.00	0.80	1.00
1388544_at	2,3-bisphosphoglycerate mutase	Bpgm	296973	1.04	0.27	8.8E-01	1.2E-02	1.00	1.00	1.00	1.00
1390388_at	ferrochelatase (predicted)	Fech_predicted	361338	1.06	0.27	8.2E-01	3.1E-02	1.00	1.00	1.00	1.00
1395222_at	FCH domain only 2 (predicted)	Fcho2_predicted	309129	0.19	0.27	2.8E-01	6.9E-05	0.00	1.00	0.00	1.00
1398622_at	cysteine-rich motor neuron 1 (predicted)	Crim1_predicted	298744	0.45	0.27	1.5E-01	8.3E-05	1.00	1.00	1.00	1.00
1386212_at	spectrin alpha 1	Spna1	289257	1.20	0.26	8.6E-01	6.1E-02	0.00	0.00	0.40	0.89
1383551_at	2,3-bisphosphoglycerate mutase	Bpgm	296973	1.00	0.26	1.0E+00	5.4E-02	1.00	1.00	0.80	1.00
1377423_at	Transcribed locus			1.37	0.26	8.8E-01	1.6E-02	0.00	0.00	0.00	0.61
1394109_at	Thrombospondin 1	Thbs1	445442	0.68	0.26	4.0E-01	2.3E-05	1.00	1.00	1.00	1.00
1391448_at	cysteine-rich motor neuron 1 (predicted)	Crim1_predicted	298744	0.44	0.26	2.0E-01	6.9E-05	1.00	1.00	1.00	1.00
1379203_at	transcription factor Dp 2 (predicted)	Tcfdp2_predicted	300947	0.80	0.25	5.2E-01	4.9E-02	1.00	1.00	0.60	1.00
1370176_at	trafficking protein, kinesin binding 2	Trak2	171086	0.45	0.25	9.8E-02	1.4E-03	1.00	1.00	0.90	1.00
1386463_at	Transcribed locus			0.15	0.25	1.7E-01	7.4E-05	0.00	1.00	0.20	1.00
1383853_at	dual-specificity tyrosine-(Y)-phosphorylation regulated kinase 3	Dyrk3	304775	0.60	0.25	7.6E-01	4.2E-02	0.00	0.00	0.00	0.61
1392359_at	Cytoskeleton associated protein 5	Ckap5	311191	0.29	0.25	5.7E-01	2.2E-02	0.00	0.00	0.00	0.89
1384805_at	defensin beta 24	Defb24	641632	0.24	0.24	1.1E-01	8.6E-05	0.00	0.33	0.00	0.50
1382106_at	Chemokine (C-C motif) ligand 6	Ccl6	287910	0.29	0.24	2.2E-01	7.8E-05	0.17	1.00	0.80	1.00
1377114_at				0.34	0.24	2.7E-02	1.1E-07	1.00	1.00	1.00	1.00
1368338_at	CD52 antigen	Cd52	117054	0.71	0.24	4.0E-01	7.2E-02	0.00	0.00	0.40	0.94
1373093_at	ERBB receptor feedback inhibitor 1	Errfi1	313729	0.62	0.23	1.3E-01	3.4E-02	1.00	1.00	0.20	0.56
1387756_s_at	hemogen	Hemgn	113882	0.73	0.23	4.9E-01	3.0E-02	1.00	0.67	1.00	1.00
1390991_at	hemoglobin, theta 1 (predicted)	Hbq1_predicted	303007	0.95	0.23	9.6E-01	3.2E-02	0.33	0.33	1.00	1.00
1368682_at	synaptic vesicle glycoprotein 2a	Sv2a	117559	0.47	0.23	9.1E-02	8.7E-03	1.00	1.00	0.20	0.94
1389353_at	sema domain, transmembrane domain (TM), and cytoplasmic domain, (semaphorin) 6D (predicted)	Sema6d_predicted	311384	0.39	0.23	1.2E-01	3.8E-04	1.00	1.00	0.80	1.00
1386922_at	carbonic anhydrase 2	Ca2	54231	0.72	0.23	1.1E-01	1.1E-02	1.00	1.00	1.00	1.00
1392405_at	transcription factor Dp 2 (predicted)	Tcfdp2_predicted	300947	1.94	0.22	9.5E-02	2.5E-02	1.00	1.00	1.00	1.00
1367912_at	latent transforming growth factor beta binding protein 1	Ltbp1	59107	0.49	0.22	1.9E-01	1.4E-05	1.00	1.00	1.00	1.00
1387656_at	solute carrier family 4, member 1	Slc4a1	24779	1.28	0.22	8.5E-01	3.6E-02	0.67	1.00	1.00	1.00
1378520_at	B-cell leukemia/lymphoma 11B (predicted)	Bcl11b_predicted	314423	0.62	0.22	2.3E-01	1.0E-02	1.00	1.00	0.10	0.89
1382472_at				0.43	0.22	9.2E-02	5.3E-05	1.00	1.00	1.00	1.00
1374330_at	similar to Kell protein (predicted)	RGD1565470_predicted	297025	1.13	0.22	7.3E-01	2.2E-02	0.00	0.00	0.40	1.00
1389160_at	erythroid associated factor (predicted)	Eraf_predicted	293522	3.65	0.21	6.6E-01	5.2E-02	0.00	0.00	1.00	1.00
1398577_at	similar to RIKEN cDNA 1300017J02	RGD1310507	315963	0.39	0.20	2.5E-01	8.1E-03	0.00	0.00	0.30	1.00
1383557_at	Transcribed locus			0.38	0.20	5.7E-02	1.9E-06	1.00	1.00	1.00	1.00
1367673_at	selenium binding protein 2	Selenbp1	140927	1.24	0.20	8.6E-01	7.6E-03	0.00	0.00	0.60	1.00
1390800_a_at	caveolin 2	Cav2	363425	0.08	0.19	2.1E-01	7.6E-03	0.33	1.00	0.40	1.00
1389783_s_at	similar to Fc fragment of IgG binding protein; IgG Fc binding protein	RGD1311906	292746	0.68	0.18	6.0E-01	2.6E-02	0.67	1.00	0.00	0.50
1368052_at	hydroxysteroid 11-beta dehydrogenase 1	Hsd11b1 /// Tspan8	171048	1.67	0.18	7.1E-01	5.5E-02	0.00	0.00	0.80	1.00
1391735_at	Transcribed locus			0.34	0.18	6.4E-02	2.1E-05	1.00	1.00	0.20	1.00
1369716_s_at	lectin, galactose binding, soluble 5	Lgals5 /// Lgals9	25475	1.36	0.18	1.9E-01	2.8E-03	1.00	1.00	1.00	1.00
1368751_at	potassium voltage-gated channel, delayed-rectifier, subfamily S, member 3	Kcns3	83588	0.37	0.17	5.0E-02	1.4E-05	1.00	1.00	1.00	1.00
1381160_at	Transcribed locus			0.16	0.15	1.3E-01	2.2E-06	0.00	1.00	0.00	1.00
1381487_at	angiopoietin 1	Angpt1	89807	0.50	0.14	6.5E-01	1.1E-02	0.33	0.67	0.00	0.50
1368753_at	calcium/calmodulin-dependent protein kinase kinase 2, beta	Camkk2	83506	0.59	0.14	8.3E-01	1.1E-04	0.00	0.33	0.00	1.00
1382618_at	erythrocyte protein band 4.2 (predicted)	Epb4.2_predicted	362202	0.71	0.14	7.7E-01	1.8E-02	0.00	0.00	0.10	0.89
1372374_at	carbonic anhydrase 1 (predicted)	Car1_predicted	310218	0.20	0.12	3.4E-01	2.6E-02	0.00	0.33	0.00	0.72
1395198_at	Zinc finger protein 313	Zfp313	362277	0.30	0.11	3.1E-01	5.6E-05	0.00	0.67	0.00	0.78
1392361_at	Transcribed locus			0.12	0.11	1.4E-02	5.9E-04	0.00	0.00	0.00	0.78
1374160_at	similar to RIKEN cDNA 1110063G11 (predicted)	RGD1311960_predicted	305095	0.97	0.09	9.6E-01	2.1E-04	0.83	0.33	1.00	1.00
1380183_at	pancreatic trypsin 1	Prss1	24691	0.91	0.08	9.2E-01	3.5E-04	0.00	0.00	0.00	0.78
1390216_a_at	Transcribed locus			0.15	0.05	8.4E-02	5.4E-05	0.00	0.00	0.00	0.78

				Fold Change		False Discovery Rate		Average Present Call			
				Strain Effect	Strain+Rejection Effect						
Probe Set ID	Gene Title	Gene Symbol	Entrez Gene ID	Native BN / DA	Allograft / Isograft	Native BN / DA	Allograft / Isograft	Native BN	Native DA	Allograft	Isograft
1369142_at	bone gamma-carboxyglutamate protein 2	Bglap2	25295	0.05	0.02	1.3E-01	6.5E-09	0.00	1.00	0.00	1.00

C. Overlap

Probe Set ID	Gene Title	Gene Symbol	Entrez Gene ID	Native BN / DA	Allograft / Isograft	Native BN / DA	Allograft / Isograft	Native BN	Native DA	Allograft	Isograft
1370428_x_at	RT1 class Ib, locus Aw2	RT1-Aw2	24737	116.65	97.43	1.4E-02	9.6E-08	1.00	1.00	1.00	0.78
1381593_x_at	RT1 class II, locus Ba	RT1-Ba	309621	88.70	45.16	2.5E-02	5.4E-07	1.00	0.00	1.00	0.00
1374959_at	NAD(P)H dehydrogenase, quinone 2	Nqo2	291084	21.08	53.10	3.3E-02	9.1E-07	1.00	0.00	1.00	0.00
1392334_at	RT1 class II, locus Ba	RT1-Ba	309621	51.80	27.39	1.4E-02	6.5E-09	1.00	0.00	1.00	0.00
1387992_at	leukocyte immunoglobulin-like receptor, subfamily B (with TM and ITIM domains), member 3	Lilrb3	361493	41.82	27.88	2.5E-02	1.6E-04	1.00	0.00	1.00	0.00
1374924_at	UPF3 regulator of nonsense transcripts homolog B (yeast) (predicted)	Upf3b_predicted	313449	39.15	21.02	8.9E-02	1.4E-04	1.00	0.00	1.00	0.00
1392172_at	chemokine (C-C motif) ligand 9	Ccl9	360579	19.87	6.65	7.8E-02	6.0E-04	1.00	0.00	1.00	0.00
1387839_at	Cullin 2 (predicted)	Cul2_predicted	361258	18.16	9.63	2.8E-02	2.7E-03	1.00	0.00	0.80	0.00
1377655_at	Similar to zinc finger protein 40	LOC690661	690661	17.37	9.77	2.7E-02	1.2E-03	1.00	0.00	0.50	0.00
1370964_at	argininosuccinate synthetase	Ass	25698	13.75	15.39	3.2E-02	2.8E-05	1.00	0.00	1.00	0.00
1367974_at	annexin A3	Anxa3	25291	15.04	13.64	1.5E-02	4.0E-07	1.00	0.17	1.00	0.72
1395366_at	Transcribed locus			12.55	7.53	9.0E-03	1.4E-03	1.00	0.33	1.00	0.00
1380948_at	Transcribed locus			12.51	9.15	2.3E-02	5.2E-03	1.00	1.00	1.00	0.44
1394846_at	similar to killer activatory receptor-like protein p91D (predicted)	RGD1562625_predicted	502291	4.63	12.02	9.3E-02	3.7E-05	1.00	0.00	0.80	0.00
1382257_at	Uridine monophosphate synthetase	Umps	288051	11.60	7.34	2.3E-02	1.9E-06	1.00	1.00	1.00	0.94
1387799_at	FXYD domain-containing ion transport regulator 2	Fxyd2	29639	8.85	9.88	3.8E-02	9.3E-07	1.00	1.00	1.00	1.00
1370493_a_at	leukocyte immunoglobulin-like receptor, subfamily B (with TM and ITIM domains), member 3	Lilrb3	361493	8.79	9.34	6.8E-02	5.7E-05	1.00	1.00	1.00	1.00
1374298_at	Transcribed locus			9.21	7.27	3.2E-02	5.9E-04	1.00	0.00	1.00	0.67
1383331_at	Transcribed locus			9.10	4.68	3.0E-02	2.4E-03	1.00	0.33	1.00	0.00
1370682_at	leukocyte immunoglobulin-like receptor, subfamily B (with TM and ITIM domains), member 3	Lilrb3	361493	9.02	8.52	3.8E-03	1.1E-07	1.00	1.00	1.00	1.00
1377539_at	Immunoglobulin superfamily, member 4B (predicted)	Igsf4b_predicted	360882	8.14	4.53	9.4E-02	1.6E-02	1.00	0.00	1.00	0.06
1388102_at	leukotriene B4 12-hydroxydehydrogenase	Ltb4dh	192227	3.80	8.07	5.6E-02	2.0E-04	1.00	0.00	1.00	0.00
1380346_at	serine (or cysteine) proteinase inhibitor, clade B, member 1a	Serpinb1a	291091	3.19	7.81	5.6E-02	1.9E-04	1.00	1.00	1.00	1.00
1372293_at	Transcribed locus			7.74	4.88	3.3E-02	3.8E-05	1.00	1.00	1.00	1.00
1381336_at	Transcribed locus			7.66	3.14	7.7E-02	1.7E-03	1.00	0.33	1.00	0.61
1398358_a_at	similar to integrin beta-5 (predicted)	RGD1563276_predicted	498091	3.44	7.36	7.2E-02	1.2E-03	1.00	0.00	0.70	0.00
1385397_at	Ab1-219	LOC499991	499991	7.35	6.50	2.6E-02	1.5E-03	1.00	0.67	1.00	1.00
1372069_at	ankyrin repeat domain 15	Ankrd15	309429	7.19	3.53	1.8E-02	6.1E-05	1.00	0.50	1.00	0.89
1377851_at	RT1 class Ib, locus Aw2	RT1-Aw2	24737	3.51	6.84	4.6E-02	1.6E-03	1.00	0.00	1.00	0.00
1368700_at	phospholipase C-like 1	Plcl1	84587	6.67	3.08	9.8E-02	8.1E-02	1.00	0.00	0.60	0.00
1385842_at	interleukin 1 family, member 8 (predicted)	Il1f8_predicted	362076	6.13	5.63	3.4E-02	7.4E-05	1.00	0.33	1.00	1.00
1391399_at	Transcribed locus			3.09	5.62	3.0E-02	5.2E-05	1.00	1.00	1.00	1.00
1379223_x_at	Transcribed locus			3.32	5.24	2.9E-02	7.8E-06	1.00	1.00	1.00	1.00
1367627_at	glycine amidinotransferase (L-arginine:glycine amidinotransferase)	Gatm	81660	3.65	5.23	5.7E-02	6.7E-06	1.00	1.00	1.00	1.00
1383561_at	ligase IV, DNA, ATP-dependent (predicted)	Lig4_predicted	290907	5.19	4.32	3.4E-02	7.6E-05	1.00	1.00	1.00	1.00
1385047_x_at	Complement component 6	C6	24237	5.02	3.92	4.9E-02	3.0E-07	1.00	1.00	1.00	1.00
1375080_at	similar to mitochondrial hepatocellular carcinoma-downregulated carrier protein	LOC684556	684556	4.98	4.73	3.2E-02	1.7E-05	1.00	0.67	1.00	0.44
1380546_at	similar to hypothetical protein FLJ10986	LOC298250	298250	3.05	4.86	2.5E-02	1.0E-05	1.00	0.50	1.00	0.06
1386938_at	alanyl (membrane) aminopeptidase	Anpep	81641	4.57	4.04	3.1E-02	1.2E-05	1.00	1.00	1.00	1.00
1383117_at	peroxisomal membrane protein 4	Pxmp4	282634	4.57	4.48	2.6E-02	1.9E-06	1.00	1.00	1.00	0.94
1393688_at	Complement component 6	C6	24237	4.55	3.63	5.8E-02	3.7E-06	1.00	1.00	1.00	1.00
1382926_s_at	Transcribed locus	Pum2	298874	3.31	4.34	8.3E-02	6.9E-05	1.00	1.00	1.00	1.00
1386064_at	Transcribed locus			4.34	3.12	3.3E-02	1.3E-04	1.00	1.00	1.00	1.00
1379306_at	Retinoblastoma-like 2	Rbl2	81758	4.30	3.60	3.9E-02	5.2E-05	0.67	0.17	1.00	0.39
1377034_at	serine (or cysteine) proteinase inhibitor, clade B, member 1a	Serpinb1a	291091	3.62	4.01	5.1E-02	1.6E-04	1.00	1.00	1.00	1.00
1367614_at	annexin A1	Anxa1	25380	3.13	3.81	6.7E-02	3.0E-05	1.00	1.00	1.00	1.00
1378347_at	Transcribed locus			3.77	3.45	9.4E-02	2.7E-02	1.00	0.33	1.00	0.44
1387125_at	S100 calcium binding protein A9 (calgranulin B)	S100a9	94195	0.32	3.55	6.1E-02	7.1E-02	1.00	1.00	1.00	1.00
1370904_at	major histocompatibility complex, class II, DM alpha	Hla-dma	294274	3.20	3.46	5.6E-02	3.5E-05	1.00	1.00	1.00	1.00
1380768_at	Transcribed locus			3.44	3.04	3.5E-02	3.7E-05	1.00	1.00	1.00	1.00
1374626_at	leucine-rich alpha-2-glycoprotein 1	Lrg1	367455	3.23	3.20	6.7E-02	1.9E-03	1.00	0.67	1.00	1.00
1373270_at	WD repeat domain, phosphoinositide interacting 1 (predicted)	Wipi1_predicted	303630	3.06	3.04	7.8E-02	7.6E-05	1.00	0.00	1.00	0.78
1392778_at	Transcribed locus, weakly similar to XP_001056676.1 similar to Discs large homolog 5			3.06	3.01	2.7E-02	6.5E-05	1.00	1.00	1.00	1.00
1385551_at				0.33	0.25	4.1E-02	8.7E-05	0.17	1.00	0.00	1.00
1381646_at				0.21	0.33	1.8E-02	1.5E-04	0.67	1.00	1.00	1.00
1381893_at	ATPase, H transporting, lysosomal V1 subunit B2	Atp6v1b2	117596	0.28	0.33	6.0E-02	2.4E-02	0.00	1.00	0.00	0.89
1392702_at	Transcribed locus, weakly similar to XP_573972.1 similar to RIKEN cDNA 8430426H19 [Rattus norvegicus]			0.25	0.33	3.3E-02	4.0E-06	1.00	1.00	1.00	1.00
1380740_at	Asparagine-linked glycosylation 12 homolog (yeast, alpha-1,6-mannosyltransferase) (predicted)	Alg12_predicted	315212	0.25	0.33	7.3E-02	1.1E-03	0.00	1.00	0.20	1.00
1374280_at	cerebellin 2 precursor protein	Cbln2	291388	0.25	0.33	5.0E-02	2.2E-04	1.00	1.00	0.60	1.00
1384743_at	Transcribed locus			0.32	0.17	7.0E-02	3.7E-06	1.00	1.00	1.00	1.00
1375573_at	Myeloid/lymphoid or mixed-lineage leukemia 5 (trithorax homolog, Drosophila)	Mll5	311968	0.32	0.30	7.7E-02	1.1E-04	1.00	1.00	1.00	1.00
1395067_at	Transcribed locus			0.29	0.32	4.3E-02	1.6E-04	1.00	1.00	0.60	1.00
1374429_at	Transcribed locus			0.18	0.32	9.8E-03	1.1E-04	1.00	1.00	1.00	1.00
1370464_at	ATP-binding cassette, sub-family B (MDR/TAP), member 1A	Abcb1a	170913	0.20	0.30	5.0E-02	1.7E-04	0.33	1.00	0.00	1.00
1380531_at	Synaptobrevin-like 1	Sybl1	85491	0.24	0.30	2.8E-02	8.0E-05	0.83	1.00	0.40	1.00
1397589_at	Transcribed locus			0.23	0.30	2.7E-02	3.5E-04	1.00	1.00	1.00	1.00
1369926_at	glutathione peroxidase 3	Gpx3	64317	0.19	0.30	5.0E-02	1.2E-04	1.00	1.00	0.60	1.00
1386679_at	Transcribed locus			0.29	0.25	3.9E-02	1.2E-05	1.00	1.00	1.00	1.00
1367857_at	fatty acid desaturase 1	Fads1	84575	0.29	0.18	7.5E-02	1.3E-05	1.00	1.00	1.00	1.00
1376071_at	Transcribed locus			0.20	0.29	2.9E-02	1.1E-04	1.00	1.00	1.00	1.00
1371171_at	RT1 class Ib, locus Aw2	RT1-Aw2	24737	0.14	0.29	2.5E-02	1.4E-05	1.00	1.00	1.00	1.00
1371078_at	RT1 class Ib, locus Aw2	RT1-Aw2	24737	0.28	0.24	3.5E-02	1.2E-04	1.00	1.00	0.60	1.00
1398330_at	syntaxin binding protein 1	Stxbp1	25558	0.28	0.27	3.6E-02	5.5E-06	1.00	1.00	1.00	1.00
1395436_at	Protein phosphatase 3, catalytic subunit, alpha isoform	Ppp3ca	24674	0.18	0.28	3.9E-02	1.4E-04	0.00	1.00	0.20	0.94

				Fold Change		False Discovery Rate		Average Present Call			
				Strain Effect	Strain+Rejection Effect						
Probe Set ID	Gene Title	Gene Symbol	Entrez Gene ID	Native BN / DA	Allograft / Isograft	Native BN / DA	Allograft / Isograft	Native BN	Native DA	Allograft	Isograft
1379150_at	Cytoplasmic FMR1 interacting protein 2 (predicted)	Cyfip2_predicted	303073	0.27	0.23	3.6E-02	3.5E-04	1.00	1.00	0.30	1.00
1394805_at	Exportin 6	Xpo6	293476	0.22	0.27	3.0E-02	3.5E-04	0.00	1.00	0.00	0.61
1395166_at	EH-domain containing 2	Ehd2	361512	0.11	0.27	6.3E-02	3.0E-04	0.17	1.00	0.00	1.00
1385166_at	Transcribed locus			0.26	0.25	3.0E-02	5.4E-05	1.00	1.00	0.30	1.00
1384165_at	Coagulation factor II (thrombin) receptor-like 2	F2rl2	29636	0.26	0.21	9.4E-02	1.4E-06	1.00	1.00	1.00	1.00
1372923_at	peroxisomal biogenesis factor 11b	Pex11b	310682	0.22	0.26	2.5E-02	1.7E-07	1.00	1.00	1.00	1.00
1378887_at	Transcribed locus			0.23	0.25	9.7E-02	5.4E-05	0.33	1.00	0.00	1.00
1374557_at	CG6210-like	LOC362065	362065	0.25	0.24	7.6E-02	2.3E-04	0.00	1.00	0.00	0.56
1373267_at	Sh3 domain YSC-like 1 (predicted)	Sh3yl1_predicted	362724	0.12	0.24	2.8E-02	1.3E-04	1.00	1.00	1.00	1.00
1398598_at	dystonin (predicted)	Dst_predicted	316313	0.24	0.23	7.2E-02	7.8E-05	1.00	1.00	1.00	1.00
1377479_at	Transcribed locus			0.22	0.19	2.8E-02	1.5E-04	1.00	1.00	1.00	1.00
1369836_at	interferon-induced protein with tetratricopeptide repeats 1	Ifit1	56824	0.15	0.21	3.5E-02	8.5E-04	0.00	1.00	0.10	1.00
1392972_at	triple functional domain (PTPRF interacting)	Trio	310192	0.11	0.21	4.4E-03	8.7E-06	1.00	1.00	0.80	1.00
1388276_at	histone cluster 1, H4a (predicted)	Hist1h4a_predicted	364716	0.19	0.17	3.3E-02	2.7E-05	1.00	1.00	0.70	1.00
1383977_a_at				0.19	0.17	9.8E-03	1.1E-03	0.00	1.00	0.00	1.00
1373810_at	phospholipase A2, group XIIA (predicted)	Pla2g12a_predicted	362039	0.19	0.11	1.9E-02	1.9E-06	0.33	1.00	0.00	1.00
1376193_at	Kohjirin	Chrdl1	363455	0.11	0.18	3.0E-02	5.5E-06	1.00	1.00	1.00	1.00
1381036_at	Transcribed locus			0.14	0.18	7.2E-03	5.2E-03	0.83	1.00	0.00	1.00
1377334_at	RT1 class II, locus Ba	RT1-Ba	309621	0.05	0.18	3.2E-02	1.6E-05	0.67	1.00	0.60	1.00
1383272_at	ankyrin repeat and SOCS box-containing protein 11 (predicted)	Asb11_predicted	302666	0.16	0.08	6.3E-02	8.3E-04	0.00	1.00	0.00	0.78
1391791_at	N-acylsphingosine amidohydrolase 3-like (predicted)	Asah3l_predicted	313339	0.06	0.16	2.3E-02	1.1E-07	0.00	1.00	0.10	1.00
1377642_at	caveolin 2	Cav2	363425	0.06	0.13	2.6E-02	2.1E-05	0.33	1.00	0.80	1.00
1392860_at				0.09	0.13	4.3E-02	2.7E-07	0.33	1.00	0.00	1.00
1368150_at	solute carrier family 27 (fatty acid transporter), member 2	Slc27a2	65192	0.11	0.13	3.3E-02	6.6E-06	0.83	1.00	0.80	1.00
1388955_at	EH-domain containing 2	Ehd2	361512	0.08	0.13	3.2E-02	2.7E-06	0.83	1.00	0.40	1.00
1373302_at	Transcribed locus, strongly similar to XP_580072.1 hypothetical protein XP_580072 [Rattus norvegicus]			0.07	0.12	3.4E-02	1.1E-07	0.33	1.00	0.40	1.00
1384380_at	Zinc finger, CCHC domain containing 7 (predicted)	Zcchc7_predicted	298086	0.10	0.08	3.3E-02	7.2E-06	0.33	1.00	0.00	1.00
1388176_at	camello-like 5	Cml5	114020	0.10	0.07	8.3E-02	1.8E-04	0.00	1.00	0.00	1.00
1384191_at	Cdc42 GTPase-activating protein (predicted)	Cdgap_predicted	288093	0.10	0.10	2.6E-02	2.7E-07	0.67	1.00	1.00	1.00
1372809_at	hypothetical gene supported by AF152002	LOC290595	290595	0.05	0.10	6.5E-02	4.6E-07	0.33	1.00	0.80	1.00
1369202_at	myxovirus (influenza virus) resistance 2	Mx2	286918	0.02	0.09	2.5E-02	5.2E-06	0.33	1.00	1.00	1.00
1387770_at	putative ISG12(a) protein	isg12(a)	360415	0.04	0.09	3.8E-03	3.4E-07	1.00	1.00	1.00	1.00
1396304_at	RT1 class Ib, locus Aw2	RT1-Aw2	24737	0.04	0.08	4.0E-02	2.7E-07	1.00	1.00	1.00	1.00
1387522_at	Rhesus blood group-associated A glycoprotein	Rhag	65207	0.02	0.08	7.9E-02	3.9E-04	0.00	1.00	0.00	1.00
1392954_at	RT1 class Ib, locus Aw2	RT1-Aw2	24737	0.08	0.06	3.1E-02	6.5E-05	0.00	1.00	0.00	1.00
1394620_at				0.08	0.06	7.9E-02	3.5E-05	0.00	1.00	0.00	0.78
1369110_x_at	RT1 class Ib, locus Aw2	RT1-Aw2	24737	0.05	0.06	9.0E-03	6.4E-08	1.00	1.00	1.00	1.00
1374915_at	GH regulated TBC protein 1	Grtp1	361180	0.04	0.06	1.8E-02	8.5E-08	0.00	1.00	0.00	1.00
1382437_at	RT1 class Ib, locus Aw2	RT1-Aw2	24737	0.03	0.05	6.3E-02	4.4E-05	0.00	1.00	0.00	0.78
1370429_at	RT1 class Ib, locus Aw2	RT1-Aw2	24737	0.02	0.04	4.4E-03	1.1E-07	0.33	1.00	0.50	1.00
1379435_at	deoxyguanosine kinase (predicted)	Dguok_predicted	297389	0.03	0.04	2.6E-02	4.0E-08	0.00	1.00	0.00	0.89
1398692_at	Transcribed locus			0.01	0.04	3.8E-02	1.8E-07	0.00	1.00	0.10	1.00
1398576_at				0.03	0.01	2.8E-02	6.5E-09	0.00	1.00	0.00	1.00
1392714_at	serine (or cysteine) peptidase inhibitor, clade B (ovalbumin), member 11 (predicted)	Serpinb11_predicted	304689	0.02	0.01	1.8E-02	6.6E-06	0.00	1.00	0.00	1.00
1397859_x_at	RT1 class I, A3	RT1-A3	309627	0.01	0.02	4.4E-03	6.5E-09	1.00	1.00	1.00	1.00
1393347_at	integrin alpha L	Itgal	308995	0.01	0.01	3.8E-03	6.5E-09	0.83	1.00	0.80	1.00
